# Inhibition of protein glycosylation is a novel pro-angiogenic strategy that acts via activation of stress pathways

**DOI:** 10.1038/s41467-020-20108-0

**Published:** 2020-12-10

**Authors:** Cuiling Zhong, Pin Li, Sulabha Argade, Lixian Liu, Anastasia Chilla’, Wei Liang, Hong Xin, Brian Eliceiri, Biswa Choudhury, Napoleone Ferrara

**Affiliations:** 1 Department of Pathology, University of California, San Diego, La Jolla, CA 92093 USA; 2 Department of GlycoAnalytics Core, University of California, San Diego, La Jolla, CA 92093 USA; 3 Department of Surgery, University of California, San Diego, La Jolla, CA 92093 USA

**Keywords:** Biochemistry, Glycosylation, Drug discovery, Cardiovascular biology

## Abstract

Endothelial cell (EC) metabolism is thought to be one of the driving forces for angiogenesis. Here we report the identification of the hexosamine D-mannosamine (ManN) as an EC mitogen and survival factor for bovine and human microvascular EC, with an additivity with VEGF. ManN inhibits glycosylation in ECs and induces significant changes in N-glycan and O-glycan profiles. We further demonstrate that ManN and two N-glycosylation inhibitors stimulate EC proliferation via both JNK activation and the unfolded protein response caused by ER stress. ManN results in enhanced angiogenesis in a mouse skin injury model. ManN also promotes angiogenesis in a mouse hindlimb ischemia model, with accelerated limb blood flow recovery compared to controls. In addition, intraocular injection of ManN induces retinal neovascularization. Therefore, activation of stress pathways following inhibition of protein glycosylation can promote EC proliferation and angiogenesis and may represent a therapeutic strategy for treatment of ischemic disorders.

## Abstract (web summary)

Therapeutic angiogenesis has the potential of inducing and maintaining new blood vessels and thus improving outcomes in patients with ischemic disorders. Mannosamine functions as an endothelial cell mitogen/survival factor through activation of stress pathways and might be useful to protect and regenerate the vascular endothelium in a variety of disorders.

## Introduction

Angiogenesis is a complex process involving the growth of new blood vessels from the existing vasculature and occurs in both physiological and pathological circumstances. In tumors, angiogenesis facilitates rapid growth and metastasis through delivery of nutrients and oxygen and removal of metabolic wastes^1^. Development of the vasculature requires the coordinated activation of multiple signaling pathways, including VEGF/VEGFR, angiopoietin (Ang)/Tie2, Notch, Ephrin/Eph, and PDGF/PDGFR^2,3^. Stimulating angiogenesis has the potential of facilitating treatment of a number of conditions characterized by reduced perfusion, including diabetic ulcers, myocardial, and limb ischemia^4,5^. Conversely, blocking angiogenesis is a clinically validated strategy to treat malignant tumors and intraocular neovascular disorders^1,6^.

EC metabolism is thought to play a key role in the regulation of angiogenesis in normal and pathological circumstances. Metabolic switches in ECs, such as fatty acid, glucose, and glutamine metabolism, have been reported to trigger angiogenesis^7,8^. ECs in the tumor vasculature are known to rely on glycolysis for ATP production, for instance through enhanced expression of glucose transporter GLUT1. Lowering glycolysis in tumor ECs arrests their proliferation^9^. In addition, aberrant glycosylation patterns have been documented during oncogenic transformation and progression of cancer and it has been proposed that inhibiting glycosylation may result in suppression of key angiogenesis pathways, including VEGF/VEGFR2 and Notch^10^. Evidence that has emerged in recent years points to glycans as novel angiogenesis regulators due to changes in protein glycosylation^11^. For example, the glycan-binding protein Galectin1 has been reported to interact with VEGFR2, leading to ligand-independent receptor activation, which may contribute to tumor resistance to anti-VEGF therapy^11^. Therefore, EC metabolism has been identified as a new target for anti-angiogenic therapy, particularly through inhibition of energy metabolism and glycosylation.

We hypothesized that testing the effects of metabolites on ECs may be a strategy to identify pro- and/or anti-angiogenic targets. Such approach might result in better understanding of the role of metabolism in angiogenesis and potentially also in resistance mechanism(s) toward anti-angiogenic and other therapies for cancer. Here we report that the hexosamine mannosamine (2-Amino-2-deoxy-D-mannose or ManN hereafter) inhibits protein glycosylation and yet stimulates EC proliferation in vitro. We further examine its biological effects in other in vitro and in vivo models, as well as its possible mechanisms of action.

## Results

### ManN promotes EC proliferation

We screened a library of 619 highly purified metabolites encompassing a broad spectrum of chemical entities for their ability to affect growth of bovine choroidal microvascular EC (BCEC), in the presence or in the absence of VEGF. This and similar assays have been previously employed to identify and characterize angiogenesis stimulators and inhibitors^12–14^. Under the conditions tested, little or no proliferation was detected in the absence of VEGF.

**Fig. 1:**
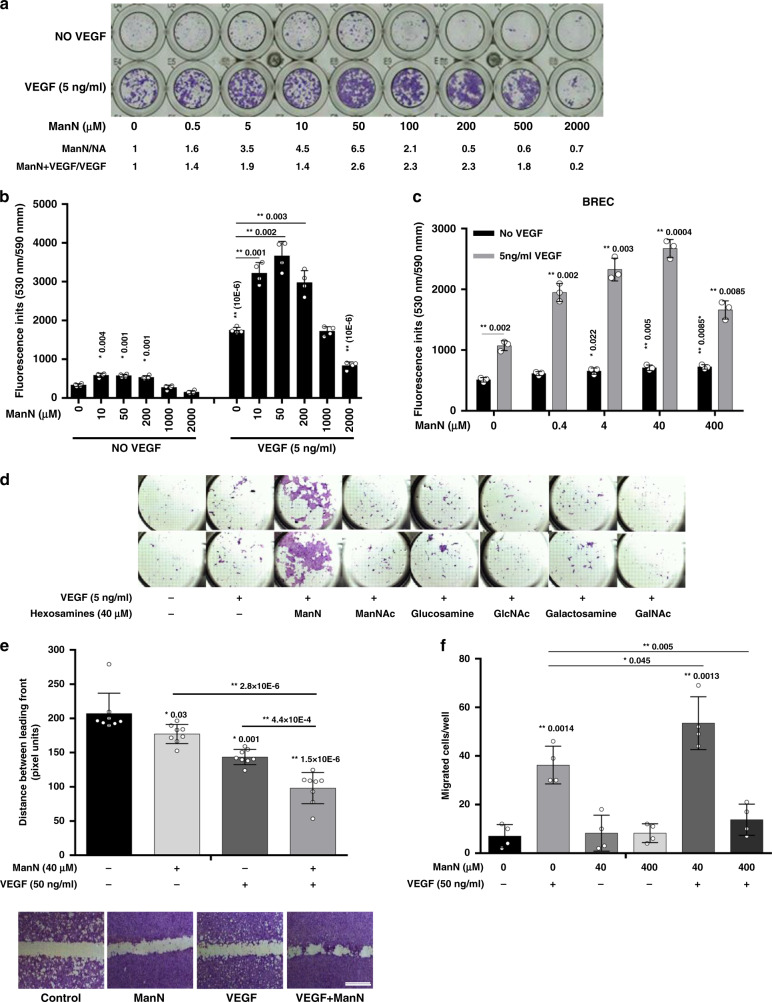
ManN is a mitogen/survival factor for eye-derived microvascular endothelial cells. **a** Bell-shaped effects of Mannosamine (ManN) on bovine choroidal microvascular endothelial cells (BCEC) proliferation. BCECs were treated with various concentrations of ManN ranging from 0.5 μM to 1 mM for 5–6 days, with or without 5 ng/ml VEGF. At the end of the experiment, cells were fixed and stained with crystal violet. Cell-covered areas in various treatment groups were quantified by ImageJ software. **b** Cell numbers were quantified by AlamarBlue® assay and fluorescence was measured at 530 nm/590 nm. *n* = 3 independent samples. **c** Effect of ManN on bovine retinal microvascular endothelial cells (BREC) proliferation. *n* = 3 biologically independent samples. **d** Effects of hexosamines other than ManN on BCEC proliferation. Each treatment group was tested in duplicate. **e** BCEC confluent monolayers were scratched with 1 ml pipet tip, washed and then incubated for 40 h in low-glucose DMEM containing 1% FBS. *n* = 3 independent samples. Scale bar = 400 µm. Images were taken and gaps between leading wound front were quantified using AxioVision LE Rel.4.4 software. Representative images from crystal violet staining are shown. **f** Effects of ManN in BCEC transwell migration assay. *n* = 4 independent samples. Asterisks indicate a significant difference compared with control. When statistical analysis was done using a different control, a line was used between specific groups. A representative experiment is shown in two independent studies. Data are means +/− SD, Statistical analysis was done by two-tailed, two-sample unequal variance *t* test. *p < 0.05, **p < 0.01. Data are provided as a Source data file.

Our initial screening was done testing each compound at the concentrations of ~1 and 10 μM (assuming a molecular mass of 100 Da for each compound), with or without 5 ng/ml VEGF, which could induce ~4–5-fold increase in cell proliferation. Six compounds of various chemical nature showed some inhibitory or stimulatory activity. We focused our analysis on one of these, ManN, a hexosamine originally identified as a component of bacterial cell wall^15^ because it showed the most potent and consistent effects. ManN had significant stimulatory effects in the 5–500 μM dose range and was also additive with VEGF in promoting BCEC proliferation. Dose-dependent effects of ManN on BCEC proliferation, in the absence or in the presence of VEGF, are shown in Fig. 1a, b. A maximal ~6.5-fold increase in EC-covered surface by ManN at 50 μM alone (Fig. 1a) or ~2.5–3-fold increase in fluorescence units upon AlamarBlue® addition (Fig. 1b) was obtained when cells were treated with 50 μM ManN and 5 ng/ml VEGF, compared to VEGF alone. AlamarBlue® detects mitochondria activity as an indication of cell viability which correlates with cell number at certain ranges^16^. The effects of ManN had a bell-shaped dose–response curve, with inhibition at higher concentrations (Fig. 1a, b). Additive effects of ManN in promoting BCEC proliferation were observed also with bFGF (Supplementary Fig. 1a) and in bovine retinal EC (BRECs) (Fig. 1c). As expected, the effects of ManN on BCEC proliferation were dependent on cellular glycolysis. The additivity between ManN and VEGF was abolished when glucose-free media was used. On the other hand, the activity of VEGF was not dependent on the glycolysis pathway (Supplementary Fig. 1b). However, there was a significant cytotoxicity with as little as 4 μM ManN, even in the presence of VEGF, in glucose-free media.

We tested various hexosamines (galactosamine, glucosamine, and their N-acetyl derivatives) alongside ManN in the BCEC proliferation assay. However, none of these hexosamines had significant stimulatory effects (Fig. 1d). We also tested several structurally related molecules such as D-isoglucosamine (fructosamine), meglumine, muramic acid, N-Acetyl-neuraminic acid (sialic acid present in all mammalian cells), glucose, and mannose. None of these molecules stimulated BCEC proliferation, with or without VEGF (Supplementary Figs. 1c and 8b).

ManN entered and accumulated inside the cells in a concentration-dependent manner. 0.66 nmol of ManN were detected in 1 mg cell lysate when BCECs were treated with 400 μM ManN for 2 h (Supplementary Table I). Following entry into the cells, ManN, but not mannose, is quickly converted to ManN-6-phosphate (ManN-6p)^17^ (Supplementary Fig. 2a, b). No incorporation of ManN was detected in N-glycans (Supplementary Fig. 2c). Although we cannot rule out the possibility that there may be differences in the degree of cell entry among hexosamines, efficient uptake of ManNAc, and mannose has been reported^18,19^.

To further characterize the effects of ManN on EC survival, proliferation, and migration, confluent BCEC monolayers were mechanically wounded. Figure 1e shows that 40 μM ManN or 50 ng/ml of VEGF significantly accelerated BCEC migration and/or proliferation as reflected by more complete closure of the “scratched” area, compared to the control group after 48 h. Similar to the proliferation assays, additivity was observed when cells were treated with both ManN and VEGF (Fig. 1e). In addition, ManN at 40 μM showed a significant additivity with VEGF in promoting BCEC migration (Fig. 1f).

We extended our observations to human retinal microvascular EC (hRMECs), HUVEC, and dermal microvascular endothelial cells (hDMVECs). ManN by itself stimulated HUVEC and hDMVECs growth (Supplementary Fig. 3a, b, f). In addition, there was a dose-dependent additivity with VEGF in all EC types tested (Supplementary Fig. 3a, b, e), with minimal toxicity even at 5 mM. Likewise, stimulation of migration and wound closure was observed in HUVEC treated with 40 μM ManN alone (migration assay) and/or in combination with 50 ng/ml VEGF (scratch assay) (Supplementary Fig. 3c, d).

### Activation of ERK, AKT, mTOR, CREB, AMPK, ACC, and eNOS is not unique to ManN

**Fig. 2:**
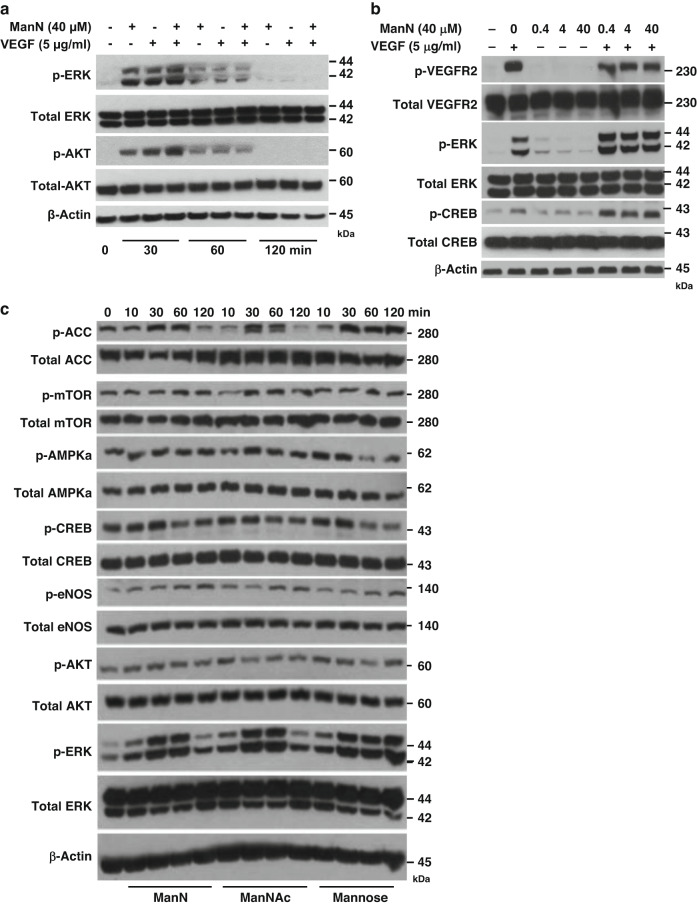
Activation of ERK, AKT, mTOR, AMPKα, CREB, ACC, and eNOS is not unique to ManN. Enhanced activation of ERK (Thr 202/Tyr 204), AKT (Ser 473), and CREB (Ser 133) in BCECs following treatment with ManN together with VEGF for various times (**a**) or following pre-treatment with ManN for 8 h, followed by VEGF stimulation for 15 min (**b**). **c** BCECs were treated with 40 μM ManN, ManNAc, or mannose for various times. Total mTOR, ACC, eNOS, AMPKα, ERK, AKT, CREB as well as phosphorylation of mTOR (Ser 2448), ACC (Ser 79), eNOS (Ser1177), AMPKα (Thr 172), ERK (Thr 202/Tyr 204), AKT (Ser 473), and CREB (Ser 133) were examined by western blot analysis. β-actin served as the loading control. Molecular weight (kDa) was labeled at the right. A representative experiment is shown from two independent studies. Data are provided as a Source data file.

It has been pointed out  that cross-talk between signaling and metabolic pathways in the vasculature, such as insulin signaling and glucose metabolism in ECs, involves AKT and STAT3 activation. Together, they affect glycolysis, EC sprouting, proliferation, and migration^20^. We examined whether ManN and/or VEGF could activate major signal transduction pathways known to promote proliferation, such as ERK, AKT, mTOR, and CREB (cAMP response element-binding protein) in BCECs. ManN activated ERK, AKT, mTOR, and CREB at 40 μM. Stimulation of ERK, AKT, and CREB was rapid and occurred within 10–30 min after adding ManN (Fig. 2a, b, c). Furthermore, we observed an enhancement in activation of ERK, AKT, and CREB when both ManN and VEGF were present compared to ManN or VEGF alone (Fig. 2a, b). We also explored whether ManN could activate the ACC (Acetyl-CoA carboxylase)/eNOS (endothelial nitric oxide synthase 3) pathway. Activation of the energy sensor AMPK (AMP-activated protein kinase) leads to eNOS activation and NO (nitric oxide) production; the latter exerts bell-shaped effects on EC proliferation^21^. Both eNOS and ACC were significantly activated by 40 μM ManN within 10–30 min (Fig. 2c). However, activation of ERK, AKT, mTOR, CREB, ACC, and eNOS was not unique to ManN. Indeed, other hexosamines such as ManNAc and mannose induced a similar activation of these signal transduction pathways (Fig. 2c). While activation of these common proliferation pathways likely contributed, we hypothesized that some unique mechanism(s) may be implicated in the EC mitogenic effects of ManN.

### ManN, but not other hexosamines tested, activates the JNK pathway

**Fig. 3:**
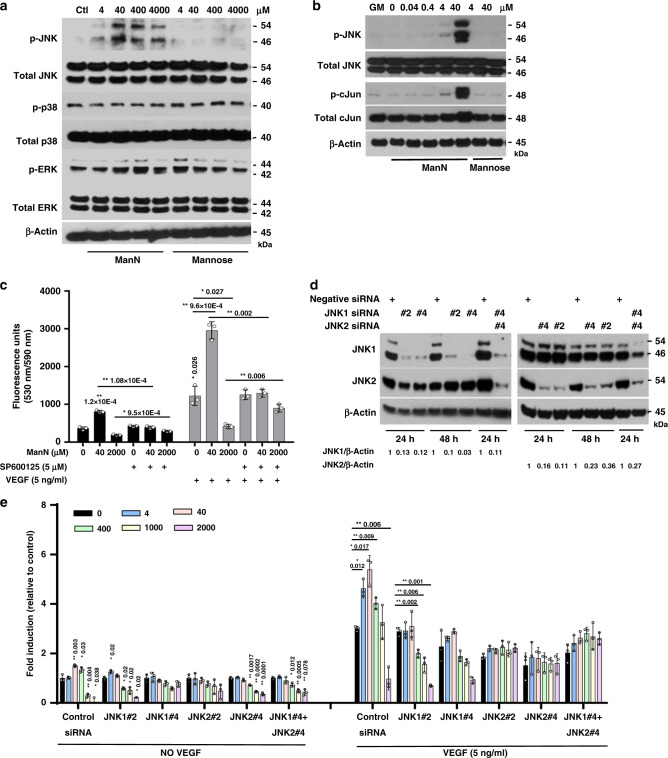
ManN specifically activates the JNK pathway in BCECs. **a** BCECs grown in growth media (GM: low-glucose DMEM containing 10% bovine calf serum (BCS), 10 ng/ml VEGF and 5 ng/ml bFGF) were switched to growth factor-free media, followed by treatment with ManN or Mannose at 4 μM–4 mM. Four hours later, cell lysates were collected and subjected to western blot analysis for phosphorylated JNK (Thr 183/Tyr 185), p38 (Thr 180/Tyr 182), and ERK (Thr 202/Tyr 204), as well as total JNK, p38, and ERK. **b** ManN, but not mannose, could activate JNK and its downstream c-Jun. β-actin served as the loading control. For each study, a representative experiment is shown from two to three independent studies. **c** BCECs plated in 96-well plates were attached, pre-treated with the specific JNK inhibitor SP600125 (5 μM) for 2 h, followed by ManN at either 40 μM or 2 mM, with or without 5 ng/ml VEGF. Six days later, cell proliferation was quantified after addition of AlamarBlue®. *n* = 3 independent samples. **d** Screening of siRNAs against JNK1 and JNK2. Twenty-four hours after siRNA transfection, BCECs were lysed and proteins were subjected to western blot analysis. β-actin served as the loading control. Quantification of target knockdown is shown. **e** A representative experiment shows that ~80% knockdown of JNK1 and/or JNK2 by two independent siRNAs was associated with a significant reduction in the stimulatory effects of ManN on BCEC proliferation. *n* = 3 independent samples. Data are means +/− SD, Asterisks indicated a significant difference compared with the control. When statistical analysis was done using a different control, a line was used between specific groups. Statistical analysis was done by two-tailed, two-sample unequal variance *t* test. *p < 0.05, **p < 0.01. Data are provided as a Source data file.

Using a series of specific pharmacological inhibitors, we identified JNK/c-jun as a signal transduction pathway uniquely activated by ManN, among hexosamines. Western blot analysis revealed that, among three MAPK family members (ERK, p38, JNK), JNK was specifically activated by ManN. When growing BCECs were switched to proliferation assay media without growth factors, JNK and its downstream c-Jun were significantly activated by ManN in a dose-dependent manner, but not by mannose (Fig. 3a, b). Treatment of BCECs with the JNK specific inhibitor SP600125 (5 μM) abolished the effects of ManN on BCEC proliferation (Fig. 3c).

Next, we tested the effects of ManN on BCECs following transfection with siRNA against JNKs (namely JNK1 and JNK2, as JNK3 is not expressed in BCECs). Knocking down ~80% of either JNK1 and/or JNK2 by two independent siRNAs against JNK1 or JNK2 abolished mitogenic effects of ManN on BCECs at μM concentrations (Fig. 3d, e), suggesting that both JNK1 and JNK2 are important in transducing stress signals.

### ManN affects protein glycosylation in endothelial cells

**Fig. 4:**
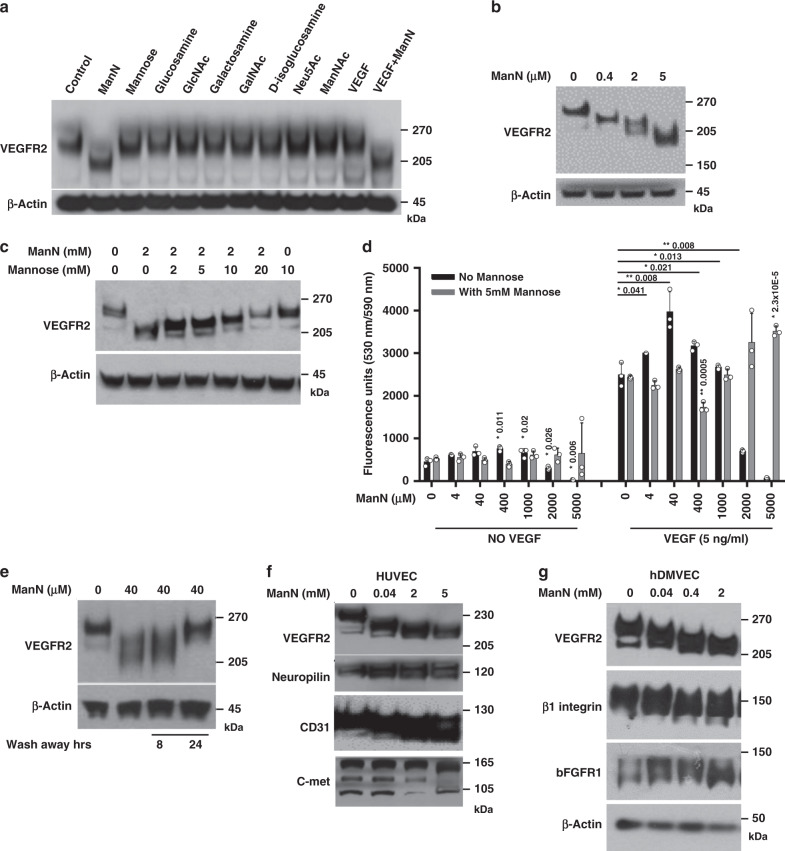
ManN affects protein glycosylation. **a** Reduction of VEGFR2 molecular mass following ManN treatment. BCECs were treated with various hexosamines, their derivatives, and monosaccharides at 40 μM or with VEGF at 5 ng/ml for 24 h. VEGFR2 western blot analysis was performed. **b** Dose-dependent effects of ManN on VEGFR2 molecular mass in BCECs. **c** Mannose could dose-dependently reverse the effect of 2 mM ManN on VEGFR2 molecular mass change, whereas mannose alone had no effect even at 10 mM. **d** 5 mM mannose could completely reverse the bell-shaped effects of ManN on BCEC proliferation with or without 5 ng/ml VEGF. BCECs plated in 96 wells were allowed to attach, followed by ManN addition. Two hours later, cells were treated with different concentrations of Mannose, with or without VEGF. Six days later, cell proliferation was quantified using AlamarBlue®. *n* = 3 independent samples. **e** Effects of ManN are reversible. BCECs, after treatment with 40 μM ManN for 24 h, were washed three times with low-glucose DMEM. Cells were kept in low-glucose DMEM for additional 8 or 24 h. VEGFR2 western blot analysis was performed. **f** Reduction of molecular mass of VEGFR2, Neuropilin-1, CD31, and c-met in HUVEC following ManN treatment at various concentrations. **g** Reduction of molecular mass of VEGFR2, β1 integrin, and bFGFR1 in hDMVECs by ManN at various concentrations. β-actin served as the loading control. Data are means +/− SD, asterisks denote a significant difference compared with the control. For each study, a representative experiment is shown from two to five independent studies. Statistical analysis was done by two-tailed, two-sample unequal variance *t* test. *p < 0.05, **p < 0.01. Data are provided as a Source data file.

**Fig. 5:**
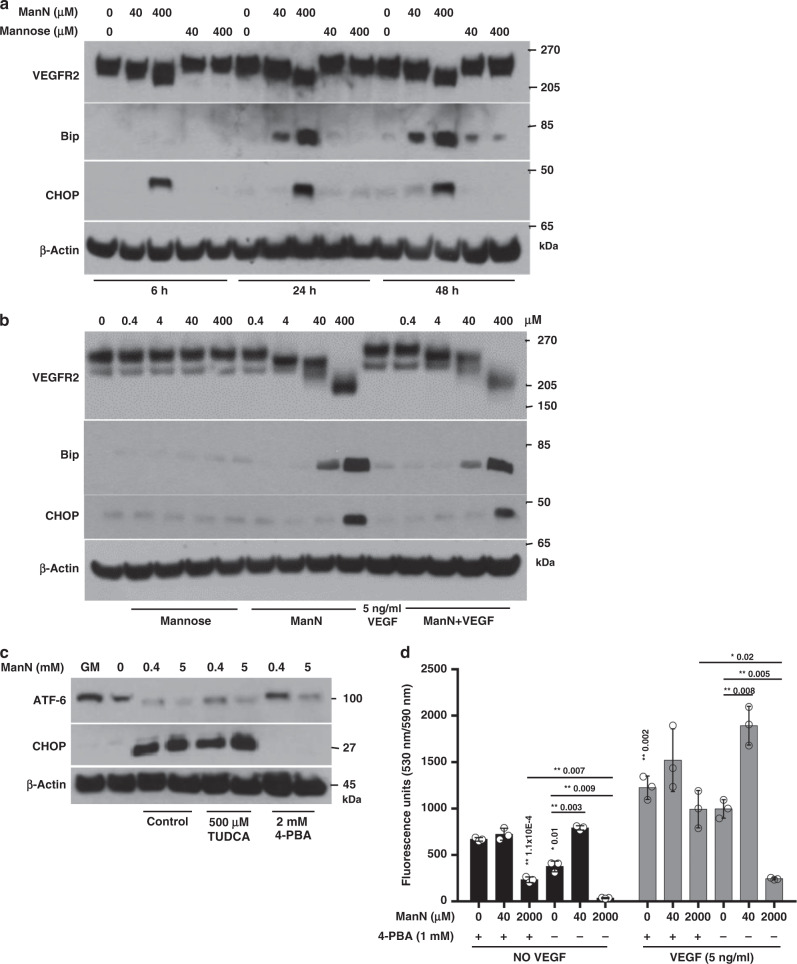
ManN specifically induces expression of unfolded protein response (UPR) responsive proteins. **a** BCECs were cultured in growth media (GM) until ~80% confluency. Media were changed to growth factor-free media containing 10% BCS in the presence or absence of 40 or 400 μM of ManN or mannose for various times. At the end of each incubation, cell lysates were collected, proteins were separated on 4–12% Bis-Tris gel for western blot analysis. **b** Cells were treated with various concentrations of ManN, mannose, 5 ng/ml VEGF, or a combination of ManN and VEGF for 24 h. Cell lysates were separated on NuPAGE 3–8% Tris-Acetate gel for western blot analysis. **c** 4-PBA, but not TUDCA, could effectively block the induction of CHOP in BCECs, accompanied by a restoration of expression of transcription factor ATF-6 upon 400 μM ManN treatment. BCECs were pre-treated with 2 mM 4-PBA or 500 μM TUDCA, two chemical chaperons. Sixteen hours later, cells were switched to growth factor-free media for 4 h in the presence of ManN. GM: growth media. **d** 4-PBA significantly blocked the bell-shaped effects of ManN on BCEC proliferation. Pre-treatment of cells with 1 mM 4-PBA for 8 h abrogated additive effects of 40 μM ManN and 5 ng/ml VEGF and protected cells from toxic effect induced by 2 mM ManN. *n* = 3 independent samples. For each study, a representative experiment is shown from two to three independent studies. Data are means +/− SD, asterisks indicate a significant difference compared with control. Statistical analysis was done by two-tailed, two-sample unequal variance *t* test. *p < 0.05, **p < 0.01. Data are provided as a Source data file.

The additivity of ManN with VEGF could potentially occur at transcriptional and/or translational level or through signal transduction pathways mediated by VEGF-VEGFR2. However, neither transcription of *VEGF*, *VEGFR2*, and *GLUT1 & 4* (Supplementary Fig. 4a) nor total VEGFR2 protein expression was significantly changed in BCECs (Figs. 4a–c, 5a, b, Supplementary Figs. 4b, 5a, 6a, 7a, 8a) when cells were treated with various concentrations of ManN for 4 h (for gene expression level) or 24 h (for protein expression level). Similar findings were obtained in BRECs (Supplementary Fig. 7b) and hRMVECs (Supplementary Fig. 7c). Biotinylation studies showed no changes in the amount of VEGFR2 on the cell surface (Supplementary Fig. 4b). However, VEGFR2 phosphorylation in response to VEGF was decreased in ManN pre-treated cells, suggesting that VEGFR2 activation was hampered, rather than enhanced in BCECs (Supplementary Fig. 4c). No ligand-independent VEGFR2 activation occurred after ManN addition (Supplementary Fig. 4c) in BCECs. The same was true also for HUVECs (Supplementary Fig. 10a) and hDMVECs (Supplementary Fig. 10b).

Interestingly, the apparent molecular mass of VEGFR2 shifted significantly following ManN treatment in both BCECs (Fig. 4a–c, e) and BRECs (Supplementary Fig. 7b), starting at 40 μM. New lower molecular weight bands (~170–200  kDa) appeared in BCECs treated with ManN in a dose-dependent manner, compared to the control (a major band at ~230 kDa and a minor band at ~210 kDa) (Figs. 4b, c, e, 5a, b; Supplementary Figs. 4b, 5a, 7a, 8a). This shift was unique to ManN among hexosamines and their derivatives (Fig. 4a). VEGF alone had no effect on molecular mass. Adding VEGF to ManN caused no additional shifts (Fig. 4a; Supplementary Fig. 6a). Lower molecular weight VEGFR2 bands are not likely degradation products since ManN removal completely reverse the effects of ManN on molecular mass after 24 h (Fig. 4e). However, based on PNGase F treatment, it appears that not all the glycosylation on VEGFR2 was abolished by ManN, at least at μM concentrations (Supplementary Fig. 7d). Experiments with the small molecule tyrosine kinase inhibitor axitinib, a potent VEGFR2 inhibitor^6^, illustrated in Supplementary Fig. 5a, b, indicate that decreases in VEGFR2 molecular mass and stimulation of BCEC proliferation by ManN are not dependent on VEGFR2 signaling.

A significant change in VEGFR2 protein mass was also observed in hRMVEC (Supplementary Fig. 7c), HUVEC (Fig. 4f), and hDMVECs (Fig. 4g) with 40 μM ManN, whereas the additive effect of ManN and VEGF on proliferation of these cells occurred at mM levels (Supplementary Fig. 3a, b, e, f).

To better understand how ManN may affect VEGFR2 post-translational modification, cells were treated with ManN in the presence of one of four monosaccharides (mannose, glucose, galactose, or fucose) at a maximum 1:10 molar ratio. These monosaccharides are known to be important in protein N-glycosylation. Our results suggest that mannose could dose-dependently block ManN effects on VEGFR2 molecular mass as well as on BCEC proliferation (Fig. 4c, d). The effects of mannose may not be limited to prevention of entry of ManN into cells via the same transporter(s) since the effects were seen when BCECs were first treated with ManN for 2 h to ensure its successful cellular uptake. Glucose, but not galactose or fucose, had similar effects as mannose (Supplementary Fig. 8a, b).

Decreases in protein mass following ManN administration in BCEC were not limited to VEGFR2. Other N-glycosylated growth factor receptors/co-receptors or adhesion molecules, including αv integrin, Neuropilin-1, VE-cadherin, and bFGFR1 (Supplementary Fig. 7a) were affected as well.

### ManN changes general protein glycosylation profiles

N-glycosylation is a complex process, dependent on multiple enzymes that act sequentially on glycoproteins to generate hybrid and high-mannose glycan structures as they transit through the secretory pathway, from ER to Golgi apparatus^22^. It plays an important role in the determination of the fate of newly synthesized glycoproteins in the ER, their correct folding, cellular destination, and proper function.

We examined several key enzymes involved in protein N-glycosylation in both ER and Golgi apparatus. α-mannosidase from Jack Bean is a broad-specificity exoglycosidase that catalyzes the hydrolysis of terminal, non-reducing α1–2, α1-3, and α1–6-linked mannose residues from oligosaccharides in both organelles, and controls conversion of high mannose to complex N-glycans, the final hydrolytic step in the N-glycan maturation pathway. This enzyme has been used to screen for potential N-glycosylation inhibitors^23^. ManN, but not other hexosamines or their derivatives, showed inhibitory activity at 400 μM (Supplementary Fig. 9a), which is considerably higher than the effective mitogenic concentrations in BCECs. No effect of ManN up to 2 mM on α- or β-glucosidases was detected (Supplementary Fig. 9b, c).

N-linked glycans from BCECs were isolated by enzymatic cleavage, followed by purification and characterization using MALDI-TOF-MS. Treatment with 40 μM ManN resulted in a significant time-dependent reduction in Man6GlcNAc2 (Man-6), Man-8, and Man-9 in total oligomannose N-glycan content compared to the untreated control, whereas a significant early phase accumulation of Man-5 and Man-7 was observed after ManN treatment (Supplementary Fig. 9d). ManN has been previously shown to inhibit lipid-linked oligosaccharide (LLO) synthesis, to change protein GPI biosynthesis and hybrid glycan production as well as to incorporate into the glycans in MDCK cells^24^. It remains to be determined whether reduction in Man-9 was partly due to LLO inhibition. Accumulation of Man-5 over time suggested that inhibition of mannosidase is unlikely the mechanism of pro-angiogenic activity in BCECs.

We next measured monosaccharide content to profile the composition of complex N-glycans. Results shown in Supplementary Fig. 9e, f indicated a significant decrease in fucose (8 h), mannose (12 h), galactose (24 h), and Neu5Ac (8 and 24 h) in ManN-treated cells compared to the untreated control cells, consistent with the inhibitory activity of ManN on overall protein N-glycosylation.

O-glycan modification is another form of post-translational modification of proteins, where a serine or threonine residue is covalently linked with a GalNAc residue^22^. The GalNAc residue can be further modified by several glycosyl-transferases acting in a sequential manner to extend the glycan chain, either branched or linearly, according to substrate specificity. The ppGalNAcT (polypeptidyl GalNAc transferase) catalyzes the transfer of a α-GalNAc from UDP-GalNAc to Ser or Thr residue of a glycoprotein, producing the Tn antigen. When the Tn antigen is generated, it can have three different fates: (i) it can be sialylated on C6 by the enzyme ST6GalNAcT; (ii) it can be substituted on C3 or C6 by a β-GlcNAc which gives rise to core-3 or core-6, respectively; or (iii) it can be galactosylated on C3 by the C1GalT1 in order to form core 1 which can also be sialylated to produce mono- or di-sialyl Core 1 O-glycan^22^.

We then performed O-glycan analysis in BCEC lysates by MALDI-Tof mass spectrometry. Due to unavailability of a unique enzyme that cleaves all different forms of O-glycan, currently, the best method is to perform reductive beta-elimination to have an understanding of the O-glycan backbone^25^. To protect from de-sialylation during mass spectral data acquisition, we performed permethylation prior to MALDI-Tof/Tof mass analysis^25^. We observed an overall decrease in O-glycosylaton following treatment with 40 μM ManN. In particular, we observed a trend of decrease toward ion intensity at *m*/*z* of 895 (Sialyl-Core 1, Galβ1-3GalNAc-), 1256 (di-sialylated Core 1), 983 [Core 2, GlcNAcβ1–6(Galβ1-3)-GalNAc-], and 1187 (di-galactosylated Core 2) (Supplementary Table I).

### ManN activates UPR by increasing Bip and CHOP expression

Asparagine-linked N-glycosylation is one of the most common modification reactions in eukaryotic cells, occurring in proteins that are co-translationally translocated across or integrated into the ER during biosynthesis^22^. After N-linked oligosaccharides are transferred to nascent proteins by the OST (oligosaccharyltransferase), ER-resident glucosidases, and mannosidases generate a series of glycan-trimming intermediates that are specifically recognized by ER-localized lectins to direct the nascent proteins into protein folding, degradation, or export pathways. One of the consequences of inhibition of protein glycosylation is compromised protein folding, leading to ER stress^26,27^. The physiological responses to the UPR are mediated by changes in gene expression, such as the regulation of ER Hsp70 chaperone BiP (also called glucose-regulated protein 78, binding of immunoglobulin protein) and another multifunctional transcription factor CHOP (CCAAT-enhancer-binding protein homologous protein)^28,29^. Impaired UPR function, for instance during aging, creates a permissive environment for protein aggregation, unresolved ER stress, and chronic inflammation^30^.

To investigate a possible ManN-mediated ER stress, we studied the expression of Bip and CHOP in ManN or mannose-treated cells by western blot analysis. Our data indicate that ManN, but not mannose or VEGF, can significantly turn on Bip expression in a concentration-dependent manner when growing cells are deprived of growth factor supply, with accumulation of Bip being evident at 24 h (Fig. 5a, b) and 48 h (Fig. 5a). CHOP induction appeared to be faster, at about 6 h in a dose-dependent manner (Fig. 5a). No synergy between ManN and VEGF in promoting Bip or CHOP expression was noted (Fig. 5b).

We tested two well-known chemical chaperons 4-PBA (4-phenylbutyric acid)^31^ and TUDCA (tauroursodeoxycholic acid)^32^ to alleviate ER stress in ManN-treated BCECs. Both were previously shown to mitigate Tunicamycin-induced PERK-eIF2α-ATF4-CHOP arm of UPR and Bip expression. We found that 2 mM 4-PBA, but not 500 μM TUDCA, could prevent the induction of CHOP expression by ManN at 400 μM and 5 mM and restore the expression of ATF-6 (Activating Transcription Factor-6) by ManN at 400 μM (Fig. 5c). Likewise, restoration of ATF-6 expression was much weaker by TUDCA compared to 4-PBA. As a transmembrane ER glycoprotein, ATF-6 is cleaved liberating a 50 kDa amino-terminal fragment that translocates to the nucleus which activates transcription of ER chaperones and ER-associated degradation components such as Bip and CHOP upon accumulation of improperly folded proteins in the ER^28^. Pre-treating cells with 1 mM 4-PBA for 4 h could effectively reverse the bell-shaped activity of ManN on BCEC proliferation in the absence or presence of VEGF. Additivity between ManN and VEGF was largely abolished (Fig. 5d).

### Effects of ManN on non-endothelial cells

**Fig. 6:**
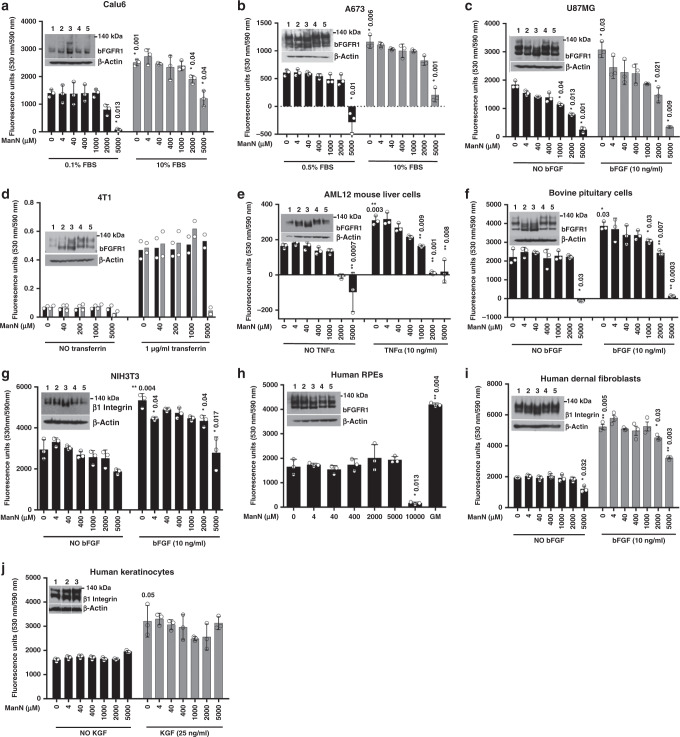
Effects of ManN on non-ECs. Effects of ManN on non-endothelial cells of bovine, mouse, or human origin. ManN did not promote growth of Calu6 (**a**), A673 (**b**), U87MG (**c**), and 4T1 (**d**) tumor cells. Ten-percent FBS was used as positive control for Calu6 and A673, whereas 10 ng/ml bFGF and 1 μg/ml human apo-transferrin were used as positive controls for U87MG and 4T1, respectively. Similarly, no increases in proliferation were induced by ManN on AML12 (**e**), bovine pituitary cells (**f**), NIH3T3 cells (**g**), human RPEs (**h**), human dermal fibroblasts (**i**), and human keratinocytes (**j**), alone or in combination with growth factors. Proliferation quantification was performed using AlamarBlue® or MTS (for 4T1 cells). *n* = 3 independent samples. Inserted are representative western blot analyses showing dose-dependent effects of ManN and mannose at 400 μM (2,4) and 2 mM (3,5) on bFGFR1 or β1 integrin (for 4T1, AML12, NIH3T3 cells, human skeletal muscle cells, human dermal fibroblasts, and human keratinocytes) molecular mass compared to the untreated control (1). β-actin served as loading control. GM: growth media. Proteins were separated on NuPAGE 3–8% Tris-Acetate gel for western blot analysis. For each study, a representative experiment is shown from two independent studies. Asterisks indicate a significant difference compared with control. When statistical analysis was done using a different control, bracket was used between specified groups. Data were means +/− SD of the mean or an average when *n* = 2. Statistical analysis was done by two-tailed, two-sample unequal variance *t* test. *p < 0.05, **p < 0.01. Data are provided as a Source data file.

To extend our observations in EC, we examined a variety of non-EC types from different species. These include NIH3T3 fibroblasts and AML12 liver cells (mouse), ARPE-19 RPE cells (human), and freshly isolated bovine pituitary cells. We also tested several human cell types related to our in vivo models such as dermal fibroblasts and keratinocytes. In addition, we screened four human or mouse cancer cell lines (A673, U87MG, Calu6, and 4T1) (Fig. 6). To examine post-translational modifications of proteins in non-ECs, we used bFGFR1 or β1 integrin to monitor molecular mass change. Similar to BCECs, ManN not mannose could induce molecular mass change in all these non-ECs (Fig. 6 inserts). However, unlike BCECs (Fig. 1b), BRECs (Fig. 1c), hRMVECs (Supplementary Fig. 3e), HUVECs (Supplementary Fig. 3b), and hDMVECs (Supplementary Fig. 3f), no proliferative effects by ManN were observed at μM to mM concentrations, alone or in combination with other growth stimulators (Fig. 6), although efficient ManN uptake and comparable levels of free ManN were detected in all cell types (Supplementary Table II). Cellular toxicity varied among different cell types, with AML12 being the most sensitive one and human RPE cells and human keratinocytes being the least sensitive ones to 5 mM level of ManN (Fig. 6e, h). A recent study reports growth inhibition by 25 mM Mannose in vitro in several tumor lines with low level of PMI (phosphomannose isomerase)^19^. At 5 mM, ManN, but not mannose, showed significant toxicity on 4T1 cells (Fig. 6d), possibly due to a higher PMI level in 4T1 relative to all the reported sensitive tumor lines.

### Similar to ManN, inhibitors of protein N-glycosylation stimulate EC growth

**Fig. 7:**
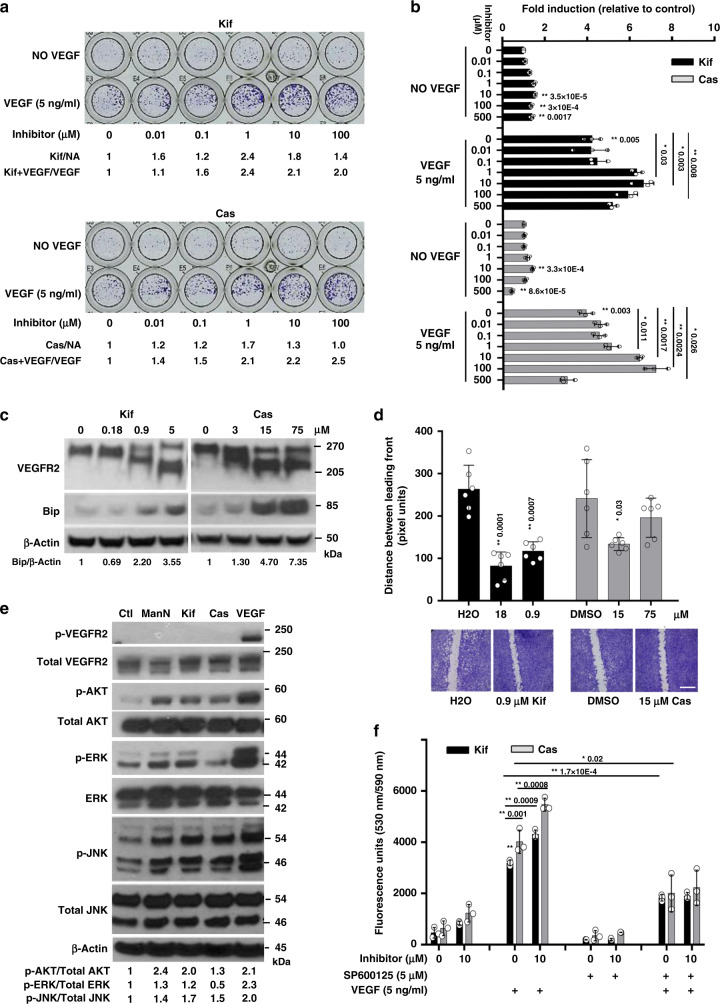
Effects of protein glycosylation inhibitors on BCEC proliferation. **a** Dose-dependent stimulation of BCEC proliferation by various inhibitors of glycosylation. Inhibitors were added at concentrations ranging from 0.01 to 100 μM for 3 days, with or without 5 ng/ml VEGF. At the end of the experiment, cells were fixed and stained with crystal violet. A representative experiment is shown. Kifunesine (Kif), an ERα-1,2-mannosidase I and Golgi α-mannosidase I inhibitor; Castanospermine (Cas), an a-glucosidase inhibitor. Cell-covered areas in various treatment groups were quantified by ImageJ software. **b** Dose-dependent effects of Kif and Cas in promoting BCEC proliferation with or without 5 ng/ml VEGF. *n* = 3 independent samples. **c** Both inhibitors reduced VEGFR2 molecular mass and induced Bip expression in a dose-dependent fashion as assessed by western blot analysis. Proteins from total cell lysates were separated using 3–8% Tris-Acetate gel. BCECs were treated with various inhibitors for 24 h. Quantification of western blots was done by densitometry. β-actin was the loading control. **d** Acceleration of closure of monolayer gaps by Kif and Cas in BCEC scratch assay, with controls for Kif (H_2_O) and Cas (DMSO). Gaps were quantified using AxioVision LE Rel.4.4 software. *n* = 3 independent samples. Scale bar = 400 µm. **e** Activation of AKT and JNK in BCECs by glycosylation inhibitors at 40 μM and VEGF at 10 ng/ml. However, Cas did not activate ERK. Quantification of phosphorylated AKT, JNK, and ERK was done by densitometry analysis relative to total protein. **f** Pre-treatment of BCECs with 5 μM SP600125 for 2 h significantly blocked the effects of both glycosylation inhibitors on BCEC proliferation. *n* = 3 independent samples. A representative experiment is shown from two to four independent studies. Data shown are means +/− SD. Statistical analysis was done by two-tailed, two-sample unequal variance *t* test. *p < 0.05, **p < 0.01. Data are provided as a Source data file.

To determine whether broad changes in protein glycosylation could promote cell proliferation, we tested two well-characterized inhibitors, Kifunensine (Kif) and Castanospermine (Cas)^33–37^. BCEC proliferation was stimulated in a dose-dependent fashion in the absence or in the presence of 5 ng/ml VEGF (Fig. 7a, b). Following treatment with Kif or Cas for 24 h, reduction in VEGFR2 molecular mass on SDS-PAGE was evident (Fig. 7c).

At 40 μM, Kif could significantly activate ERK and AKT in BCECs (Fig. 7e), HUVEC (Supplementary Fig. 10a), and hDMVECs (Supplementary Fig. 10b). Activation of ERK by Cas was less obvious in both BCECs (Fig. 7e) and hDMVECs (Supplementary Fig. 10b). However, both inhibitors were able to activate the JNK pathway in BCECs (Fig. 7e). Blocking JNK activation with 5 μM SP600125 significantly reduced the effects of both glycosylation inhibitors on proliferation of BCECs (Fig. 7f). Figure 7c illustrates a dose-dependent induction of Bip expression when growing BCECs were switched to media without growth factors for 24 h in the presence of Kif or Cas at concentrations which promoted cell proliferation.

We observed that both Kif and Cas had significant activity in the BCEC “scratch” assay, with gaps being closed more rapidly by each molecule over 48 h relative to control (Fig. 7d). The inserted panel of Fig. 7d shows representative images from an assay in which Kif or Cas was used. Quantification analysis indicated that there was significant acceleration of gap closing in a dose-dependent manner compared to controls.

### ManN, in combination with VEGF, stimulates angiogenesis in a skin injury model in mice

**Fig. 8:**
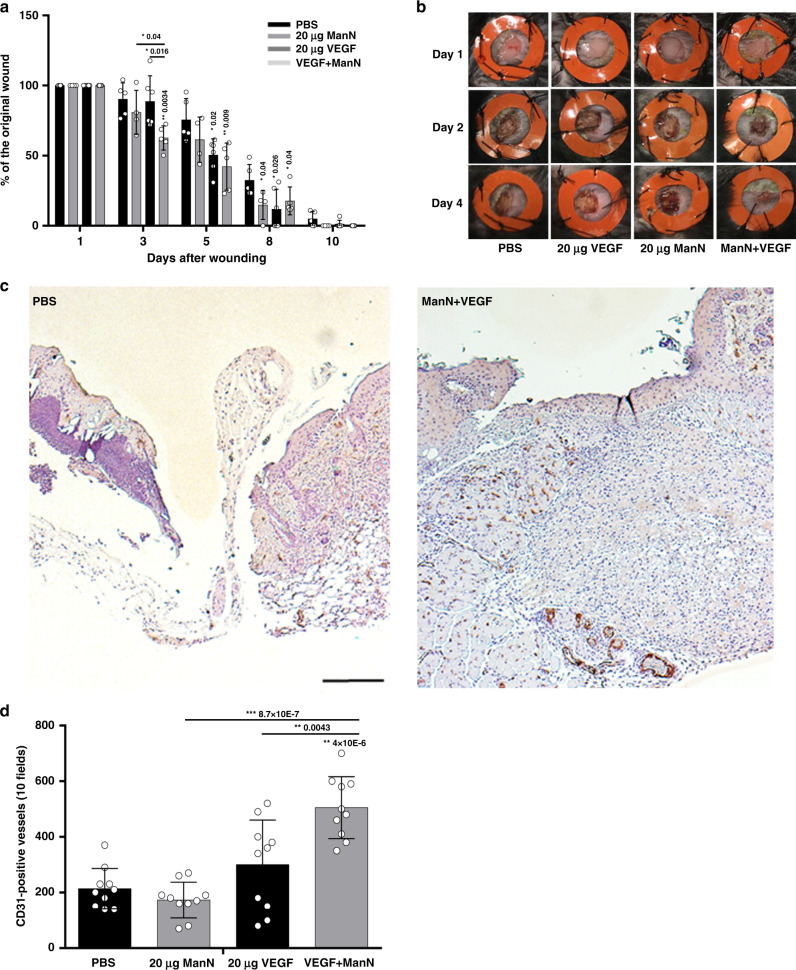
Topical application of ManN and VEGF stimulated angiogenesis and accelerates wound healing. **a** Wounds were made on the dorsal skin of mice by 6 mm punch. VEGF and ManN each was administered daily at 20 μg per wound in 25 μl PBS for the first 4 days, with PBS as control. A 10-day wound healing study with 5 mice in each group. Wound closure rate (%) was quantified by ImageJ software in two independent studies. Asterisks indicated a significant difference compared with the control at each time point. **b** A 4-day wound healing study with images of the wound healing process at day 1, day 2, and day 4. *n* = 5 animals/treatment group. **c** Representative images of immunohistochemical staining for CD31 in PBS control group and in VEGF and ManN combination groups (scale bar = 200 μm). **d** Quantification of CD31-positive blood vessel (red dotted circles) density around the wound areas was counted by eyes under microscopy (×20 magnification). Data are means +/− SD. Statistical significance was further confirmed using Wilcoxon rank-sum test between treatment groups of interest. Asterisks indicated a significant difference compared with the PBS control. For each study, a representative experiment is shown. *n* = 3 animals/treatment group. When statistical analysis was done using a different control, a line was used between specific groups. Statistical analysis was done by two-tailed, two-sample unequal variance *t* test. *p < 0.05, **p < 0.01. Data are provided as a Source data file.

To establish whether the effects of ManN on endothelial cells in vitro translate in angiogenesis stimulation in vivo, we initially investigate ManN  effects in a splinted wound model in mice. In this model, the repair process is entirely dependent on epithelialization, cellular proliferation, and angiogenesis, which closely mirror the biological processes of human wound healing^38^. We tested the effects of ManN and VEGF, alone or in combination. Topical applications of 20 μg of VEGF or 20 μg of ManN daily was done for the first 3 days after wounding. When VEGF and ManN were combined, a significant acceleration of wound closure was observed during the early phase of healing (Fig. 8a). Compared with VEGF or ManN monotherapy, the combination had a significant faster wound closure starting from day 2 (Fig. 8b and Supplementary Fig. 11a). On day 4, an average closure of the wound was 81.5%, 75.6%, 66.9%, and 29.8% in PBS-, ManN-, VEGF-, and combination-treated group, respectively (Supplementary Fig. 11a). We quantified small vessel numbers around the wound area at day 4. A significant increase in CD31-positive vessels was found in the combination group compared to PBS control, VEGF, or ManN alone (Fig. 8c, d).

These data represent proof-of-concept that ManN, in combination with VEGF, promotes angiogenesis in a skin injury model. However, it is noteworthy that in this acute model, wound closure takes place rapidly, without any treatment.

We also sought to determine whether ManN is stable in wound fluid contaminated by bacteria, a common feature of wounds. ManN was added to freshly collected wound fluid from a mouse model of skin infection with *Staphylococcus aureus*, a prevalent cause of skin and soft tissue infections in humans^39^. No significant loss of free ManN was detected following incubation with such wound fluid for up to 24 h at 37 °C (Supplementary Fig. 11c). These findings raise the possibility that ManN may be useful for treatment of infected wounds, possibly in combination with anti-microbials or other agents. Further studies are needed to test this hypothesis.

One of the known properties of VEGF is a rapid induction of vascular permeability following injection in the guinea pig skin^1^. Therefore, we wished to test whether ManN is also able to induce vascular permeability in the same assay. However, no permeability-enhancing effects were elicited by ManN when tested at 1 ng–5 μg, while 25 ng VEGF induced vascular permeability (Supplementary Fig. 11b).

### Angiogenic effects of ManN and Kif in a mouse hindlimb ischemia model

We next sought to determine whether ManN is active in a chronic ischemia model that might more specifically reflect its effects as an endothelial cell mitogen and a pro-angiogenic factor. The hindlimb ischemia model in the mouse seems especially suitable. It is a well-characterized and widely used model to study vascular wound healing, arteriogenesis, and collateral vessel growth. Several variants have been described, depending on which vessel is occluded^40,41^. The variant that we chose consists of ligation and excision of the femoral artery. This results in more severe ischemia compared to simple femoral artery ligation^40^. Occlusion of two vessels produces more severe ischemia, but has the disadvantage of inducing severe pain and distress, as well as frequent ulcerations and necrosis in mice^40^.

**Fig. 9:**
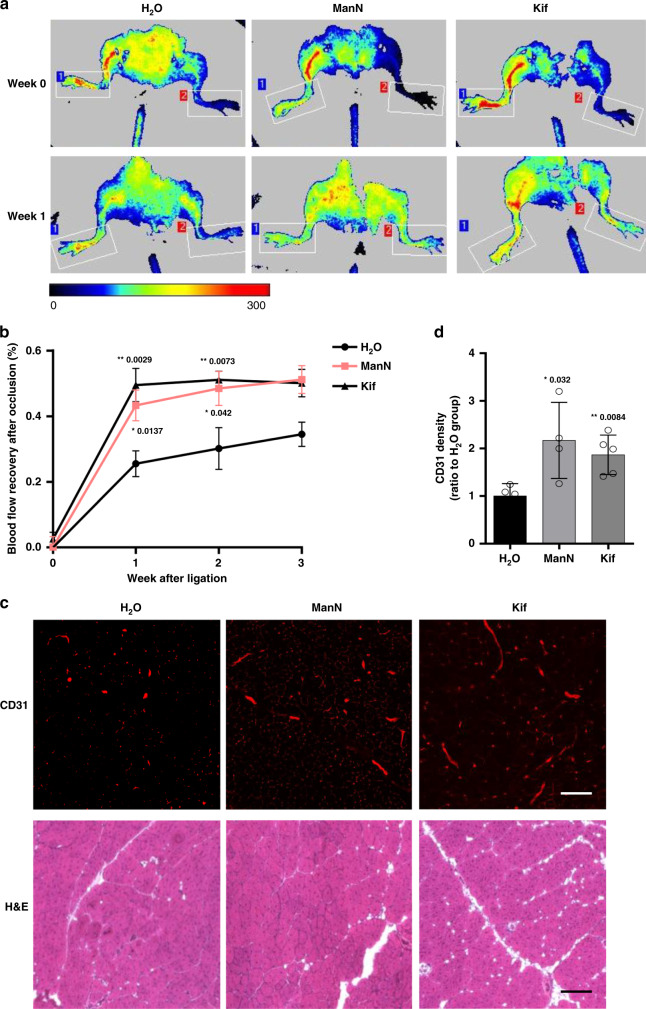
ManN accelerates blood flow recovery in a mouse ischemic hindlimb model. **a** Serial laser Doppler analysis of blood perfusion in hindlimbs of ManN-treated, Kif-treated, and control mice. Different colors were used to indicate blood perfusion in the ischemic limb (ligated; left side) to nonischemic limb (sham; right side). Representative images at week 0 and week 1 were shown. **b** Quantification of blood perfusion ratio between region 2 (ischemic; left limb) and region 1 (nonischemic; right limb), *n* = 8 animals/treatment group. **c** Three weeks after surgery, skeletal muscle tissues were harvested and fixed. CD31 immunostaining on these tissue sections was performed to label the vasculature. H&E staining was also performed. Representative CD31 staining and H&E histological image of ischemic hindlimbs 21 days after surgery were shown. Scale bar = 50 µm. **d** Quantification of vascular density by CD31 immunostaining was performed using ImageJ software, *n* = 8 animals/treatment group, three independent experiments; data are means +/− SEM. Statistical analysis was done by two-tailed, two-sample unequal variance *t* test. *p < 0.05, **p < 0.01. Data are provided as a Source data file.

We tested oral administration of ManN in this femoral artery ligation-excision model. Since Kif has been previously administered intraperitoneally for in vivo studies^42^, we employed this route in our study. Laser Doppler perfusion imaging (LDPI) was used as a non-invasive method to monitor time and extent of the blood flow recovery in the ischemic limb^43^. Serial examination of blood flow was taken with LDPI and increment of the perfusion ratio of ischemic (ligated; left side) to nonischemic (sham; right side) hindlimbs after ligation was used to indicate a recovery of blood flow. Starting immediately after surgery, mice were orally fed with 20% ManN or 1 mg/ml Kif i.p. every other day, as described in “Methods”. One week after surgery, the perfusion ratio in H_2_O-fed group indicated a blood flow recovery of ~25%, a value that is in good agreement with published data with the same type of lesion, in the same strain of mice^40,44^. However, the blood flow recovery in ManN and Kif-treated group was about 40% and 47%, respectively, which demonstrated an accelerated recovery rate of blood flow compared to H_2_O-treated mice (Fig. 9a, b). The blood perfusion ratio continued increasing to ~50% of sham-treatment limbs in 3 weeks after ManN and Kif treatment and was significantly higher than the control group (Fig. 9a, b).

Consistent with the improved blood flow, the ischemic hindlimbs of ManN-treated and Kif-treated group showed an increased blood vessel density compared to the control group, as assessed by CD31 immunostaining of the surrounding muscle tissue 3 weeks post-ligation. Compared with H_2_O-treated control group, blood vessel densities were, respectively, 2.3 and 1.8 times higher in ManN and Kif-treated groups (Fig. 9c, d).

Following oral administration, there was a relatively rapid decline in ManN plasma levels (Supplementary Fig. 12). Plasma free ManN levels reached a peak level of ~100 nmol/ml plasma at 1 h. After 3 h, only about half of that amount was detectable. Two hours after oral feeding of 20% ManN, muscle samples were taken from the ischemic legs. A significant amount of ManN reached the ischemic legs, with 0.17 +/− 0.18 nmol/mg protein of free ManN and 0.91 +/− 0.24 nmol/mg protein of ManN-6p. Interestingly, at least in BCECs, ManN effects on protein mass lasted for at least 8 h in the absence of exogenous ManN (Fig. 4e), suggesting that even a relatively brief exposure may be adequate to elicit pharmacological effects.

### ManN and Kif induce retinal neovascularization

We sought to extend our findings in cultured eye-derived EC to a suitable in vivo model system. The mouse retina has been used extensively over the past decades to study both physiological and pathological angiogenesis^41^. To obtain a detailed description of the retinal vasculature, we processed images from retinal flat mounts for vascular area fraction (ratio of area covered by blood vessels to total retinal area). Using this model, we investigated the effects of ManN in retinal neovascularization. We also tested Kif in this model because it is a water-soluble inhibitor and its mechanism of glycosylation inhibition is well-established^35^. In addition, it shares with ManN the ability to activate ERK, AKT, and stress pathways in BCEC (Fig. 7e).

**Fig. 10:**
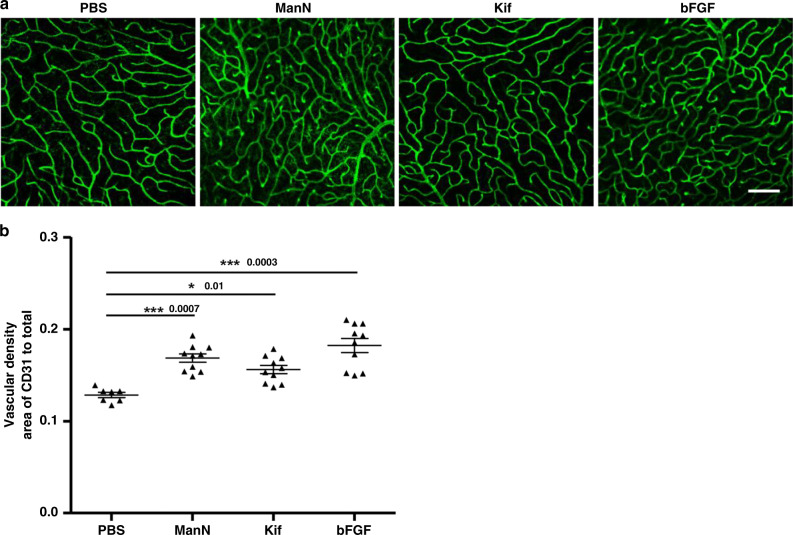
ManN promotes retinal neovascularization in mice. **a** Intravitreal injection of ManN increases blood vessel density. Adult mice were intravitreally injected once 500 ng of ManN, Kif or 200 ng of bFGF. PBS was used as vehicle control. Seven days after injection, PFA-fixed retinas were subjected to CD31 immunofluorescence. Representative images of CD31-positive vessels are shown. *n* = 10 animals/treatment group, 3 independent experiments, scale bar = 50 µm. **b** Vascular density was determined with ImageJ software, *n* = 10 animals/treatment group, 3 independent experiments. Data were means +/− SEM. Statistical analysis was done by two-tailed, two-sample unequal variance *t* test. *p < 0.05, **p < 0.01, ***p < 0.001. Data are provided as a Source data file.

Five hundred nanograms of ManN or Kif was intravitreally injected and the retinal vasculature was examined after 7 days. Intravitreally administration of 200 ng bFGF was used as a positive control in this model. In bFGF, ManN, and Kif-treated group, the density of retinal vessels was increased by about 35%, 30%, and 20%, respectively, compared to PBS group (Fig. 10a, b).

## Discussion

In the present study, we show that ManN is a hexosamine with unique ability to inhibit protein post-translational modifications, activate stress pathways, and show additivity with VEGF in promoting EC proliferation and angiogenesis. We have been able to translate in vitro effects of ManN into efficacy in multiple in vivo models. To our knowledge, effects of ManN on ECs and angiogenesis have not been previously reported. We propose a link between changes in glycosylation patterns in mammalian ECs and angiogenesis by using well-known glycosylation inhibitors together with ManN. We also provide evidence that the effects of ManN on endothelial cells are independent on VEGFR2 activation. ManN was discovered in the 1960s as a bacterial wall component^15^, and accounts for 5–10% of capsular polysaccharides^45^. The related N-acetyl mannosamine is thought to be an intermediate in the biosynthesis of sialic acids^46^. Over the years, multiple effects of ManN on enzymes, growth factor-mediated signaling, protein stability, and cell viability were documented^47–50^. Most of these effects were not unique to ManN and could be elicited by other hexosamines. In addition, they required high concentrations^45,48^. ManN was reported to have antitumor properties^47^, to stimulate osteogenic differentiation^48,51^, and to protect articular cartilage^49^. More recently, ManN was used as an intermediate in modifying various molecules/nanoparticles^52^ and in the synthesis of non-natural ManNAc analogs for the expression of thiols on cell-surface sialic acids to facilitate high-throughput screening^53^. However, to date, no effects of ManN on ECs have been described.

ManN had been previously reported to affect formation of lipid-linked oligosaccharides (LLO) in MDCK cells possibly by inhibiting the a-1,2-mannosyl transferases^24^. Upon ManN treatment, major oligosaccharides associated with the dolichol were Man5GlcNAc2 and Man6GlcNAc2 rather than Glc3Man9GlcNAc2 which was normally found in MDCK cells. In addition, ManN was reported to change protein GPI biosynthesis and hybrid glycans production in the ER^54–56^. However, none of the angiogenic-related proteins we examined are GPI-anchored. The decrease in Man-9 found in our study could be a direct result of inhibiting LLO donor synthesis, i.e., Glc3Man9GlcNAc20PP-Dol formation, which is then transferred from the dolichol donor onto the polypeptide. In our study, Man-5 was significantly increased over 24 h when cells were treated with 40 μM ManN, suggesting that ManN was not affecting α-mannosidases in the ER.

Activation of PI3K-AKT, PLCγ-ERK, and p38 is important for VEGFR2-mediated EC survival, proliferation, and migration. Other cellular metabolic stress sensors, such as AMPK (AMP-activated protein kinase), could also confer stress adaptation and promote EC survival via eNOS^57^. Our data suggest that ERK, AKT, mTOR, AMPKα, eNOS, and ACC activation is a general phenomenon for hexosamines and mannose. However, activation of the JNK/c-Jun and UPR pathways in BCECs was unique to ManN as well as the glycosylation inhibitors. Glycosylation is required for correct protein folding in the ER^26^. Interestingly, a link between LLO inhibition and activation of UPR has been reported^58^. In fact, notwithstanding the complexity of ManN actions, LLO inhibition, followed by UPR activation, seems a plausible explanation for the observed ManN effects.

Our study indicates that ECs are able to cope with acute/minor ER stress resulting from glycosylation inhibition by activating the UPR pathway. UPR detects misfolded proteins accumulated in the ER and initiates a response to maintain cellular homeostasis via induction of Bip, a major ER chaperon protein^29^. BiP binds to hydrophobic patches exposed on nascent or incompletely folded proteins that are often non-glycosylated. Indeed, we found a strong induction of Bip expression specifically by ManN among hexosamines. Similar effects on stress pathway activation were observed using two known glycosylation inhibitors Kif and Cas. Glycosylation inhibition is thought to be a new pharmacological strategy targeting metabolic pathways essential for excessive angiogenesis in various pathological conditions, and glycosylation inhibitors are expected to have anti-angiogenic and anti-metastatic properties^10,59,60^. Glycosylation has been recently shown to be important in cellular stress response and compensatory angiogenesis in response to VEGF-VEGFR2 signaling blockade^61^. Stress-induced O-GlcNAcylation was reported to promote survival in response to DNA damage, ER stress, glucose deprivation, and hypoxia in a variety of cell types^62^. In contrast, our study links glycosylation inhibition to angiogenesis promotion, and while it does not negate the above-mentioned studies, it raises the possibility that inhibiting glycosylation within the tumor microenvironment may, at least in some circumstances, result in stimulation rather than suppression of tumor angiogenesis.

Angiogenesis has the potential of providing a therapeutic benefit to patients with ischemic disorders such as peripheral arterial disease (PAD) or coronary ischemia^4^. This hypothesis led to a series of clinical trials in the past decades, testing angiogenic factors such as VEGF-A, VEGF-C, bFGF, or HGF, delivered by gene therapy or as recombinant proteins. Unfortunately, none of these studies was successful, in spite of promising preclinical studies^5^. Indeed, achieving clinical success may be contingent not only on a better understanding of EC biology but also on identifying novel molecular tools and/or approaches^63^. A renewed interest in these efforts is exemplified by recent trials with a modified RNA encoding VEGF-A in patients with type 2 diabetes^64^.

We validated our in vitro findings showing the ability of ManN and other glycosylation inhibitors to stimulate EC growth in in vivo models. ManN was able to promote angiogenesis in a mouse skin injury model, accompanied by accelerated wound closure. However, studies in chronic models, with healing impairment, will be required to establish whether ManN has therapeutic potential in wound healing.

In addition, ManN was able to stimulate angiogenesis and blood flow recovery in ischemic hindlimbs of mice. Currently, we are testing the hypothesis that combinations of ManN, or other glycosylation inhibitors, with VEGF-A, may have advantages over monotherapy for the treatment of ischemic disorders. The combination might be a promising avenue for PAD. The lack of direct permeability-enhancing effects of ManN may prove valuable, since it is expected to result in less edematous tissues. In this context, damage to lung endothelium is a central pathogenic event in the respiratory failure associated with a variety of infections, including SARS-CoV-2^65^. It is tempting to speculate that an endothelial cell mitogen like ManN, devoid of permeabilizing effects, may help protect and stabilize blood vessels and thus limit tissue damage.

The finding that intravitreal administration of ManN enhanced retinal neovascularization may suggest therapeutic applications in ocular diseases. For example, 10–15% of patients with intermediate AMD progress to the neovascular form, while the remaining patients may develop geographic atrophy (GA)^1^. Previous studies have shown that loss of choroid capillaries is frequently detected in GA, which raises the possibility that regeneration/protection of choroid capillaries may be a novel strategy for GA treatment^66^. Additional studies are warranted to determine whether ManN can promote choriocapillaris growth and/or survival, in addition to retinal angiogenesis.

## Methods

### Small molecule library

MSMLS™ (Mass Spectrometry Metabolite Library of Standards) (IROA TECHNOLOGIES, Bolton, MA; now Sigma) is a collection of 619 high-quality small molecules (purity > 95%) that span a broad spectrum of primary metabolites, including carboxylic acids, amino acids, biogenic amines, polyamines, nucleotides, coenzymes, vitamins, lipids, etc. Plates were spun at 300 × *g* after reconstitution, according to the instructions of the manufacturer.

### Chemical compounds

D-Mannosamine hydrochloride was obtained from Sigma (M4670) or Spectrum Chemical MFG Corp (M3220). 1-Amino-1-deoxy-D-Fructose hydrochloride (D-isoglucosamine) (803278), D-(+)-Galactosamine (1287722), D-(+)-Glucosamine (1294207), N-acetyl-Mannosamine (A8176), N-acetyl-galactosamine (A2795), N-Acetyl-Glucosamine (A8625), Meglumine (M9179), Muramic acid (M2503), N-Acetylneuraminic acid (A2388), D-(+)-Glucose (D9434), D-(+)-Mannose (1375182), Meglumine (M9179), Tunicamycin from *Streptomyces* sp. (T7765) and SP600125 (S5567) were obtained from Sigma. Hypure cell culture grade water used to dissolve compounds (endotoxin < 0.005 EU/ml) was obtained from Hyclone. Axitinib was obtained from Santa Cruz (SC-217679). Tauroursodeoxycholic acid (TUDCA) was from Calbiochem (1180-95-6) and 4-phenylbutyric acid (4-PBA) (P21005), Castanospermine (Cas, C3784), Kifunensine (K1140), and DMSO (D2650) were from Sigma. DMSO (D2650) was used as a solvent for Cas.

### Antibodies

Antibodies used in the present study were from Cell signaling Technology Inc. (Danvers, MA) unless otherwise specified. Total: VEGFR2 (2479), ERK (4695), p38 (9212), JNK (9252), mTOR (2983), AKT (4691), CREB (9104), CHOP (2895), ACC (3676), ATF-6 (65880), Bip (3183), AMPKα (5832), FGFR1 (9740), eNOS (9586), VE-Cadherin (2500), c-Met (3127 or 3148), Neuropilin (3725), CD31 (3528), c-Jun (9165). Phosphor-antibodies: VEGFR2 (Tyr1175, 2478, or 3770), ERK1/2 (Thr 202/Tyr 204, 4376), p38 (Thr 180/Tyr 182, 4511), JNK (Thr 183/Tyr 185, 9251), mTOR (Ser 2448, 5536), AKT (Ser 473, 4060), CREB (Ser 133, 9191), ACC (Ser 79, 3661), eNOS (Ser1177, 9571), AMPKα (Thr 172, 50081), c-Jun (Ser73, 9164), β1 integrin (4706 & 34971), αv integrin (4711), JNK1 (3708), JNK2 (4672), JNK3 (2305). Anti-β-actin was from Sigma.

### Cells

Primary human umbilical vein endothelial cells (HUVEC, passage 4–10) were obtained from Lonza (C2519AS, Lot# 234871) and cultured on 0.1% gelatin-coated plates in endothelial cell growth media (EGM) containing 2% FBS, BBE (Bovine Brain Extract), heparin, human EGF, hydrocortisone, ascorbic acid, GA-1000 (Gentamycin, Amphotericin B), and VEGF. Bovine retinal microvascular endothelial cells (BRECs, #BRMVEC-3) and bovine choroidal microvascular endothelial cells (BCECs, #BCME-4), both from VEC Technologies (Renssellaer, NY), were maintained in fibronectin-coated plates (1 µg/cm^2^). The growth medium was low-glucose DMEM, supplemented with 10% bovine calf serum (BCS), 5 ng/ml bFGF and 10 ng/ml human VEGF_165_. Cells were maintained at 37 °C in a humidified atmosphere with 5% CO_2_. bFGF (233-FB) and VEGF_165_ (293-VE) were purchased from R&D Systems. Human retinal microvascular endothelial cells (passage < 15) were from Cell Systems Corporation (Kirkland, WA). They were grown on 0.1% Gelatin-coated plates in Medium 131 containing 5% fetal bovine serum, hydrocortisone (1 µg/ml), human fibroblast growth factor (3 ng/ml), heparin (10 µg/ml), human epidermal growth factor (1 ng/ml), and dibutyryl cyclic AMP (0.08 mM) (MVGS, S-005-25, Gibco Invitrogen). The human RPE cell line ARPE-19 was from the ATCC. Cells were gently lifted in 0.025% trypsin and plated in RtEGM media (Clonetics) containing 2% FBS, L-glutamine, human bFGF, GA-1000). Once cells attached to plates, serum-free RtEGM media was used to maintain the culture for best result. ARPE-19 was obtained from ATCC (CRL-2302) and cultured according to the company’s instruction. NIH3T3 cells were obtained from ATCC (CRL-1658). Human adult dermal MVECs (CC-2543) were cultured in EGM-2MV (CC-4147, Lonza). Keratinocytes (ATCC, PCS-200-011) were cultured in dermal cell basal media (PCS-200-030) plus keratinocyte growth kit (PCS-200-040). Human primary dermal fibroblasts (ATCC PCS-201-012) were cultured in fibroblast basal medium (ATCC, PCS-201-030) plus growth kit (ATCC, PCS-201-040). Growth stimulators used in the assay included human EGF (R&D Systems, 236-EG), murine TGFα (R&D Systems, 410-MT), KGF (Sigma, K1757), or 10% FBS growth media. 4T1 cells were obtained from the ATCC (CRL-2539) and cultured in RPMI-1640 with 10% FBS (Omega Scientific, Tarzana, CA) and antibiotics. A673 (CRL-1598), A549 (CCL-185), and U87MG (HTB-14) cells were from ATCC and cultured in high glucose DMEM containing 10% FBS. FBS (S12550) was purchased from R&D Systems. BCS (SH30073.03) was obtained from Hyclone. All cell lines used in the study are negative for mycoplasma contamination by various vendors.

### Cell proliferation assays

Proliferation assays with BCECs and BRECs were performed^13^. Log-phase growing BCECs or BRECs (passage < 10) were trypsinized, re-suspended, and seeded in 96-well plates (no coating) in low-glucose DMEM supplemented with 10% bovine calf serum, 2 mM glutamine, and antibiotics (growth medium), at a density of 1200–1500 cells per well in 200 µl volume. All reagents were added at the indicated final concentrations. After 3–6 days, cells were incubated with AlamarBlue® for 4 h. Fluorescence was measured at 530 nm excitation wavelength and 590 nm emission wavelength. The experiments were repeated at least three times. To create a hypoxic condition, cells were placed in a hypoxia incubator with a mixture of gas consisting of 1% O_2_, 5% CO_2_, and 94% N_2_. On each 96-well plate, untreated and VEGF-treated (10 ng/ml) wells were included to monitor plate-to-plate variations. Twenty-percent methanol or 0.05% DMSO served as negative controls. 0.05% DMSO served as negative controls when Cas was tested in these cells. Human RMVECs and human adult DMVECs were split into Gelatin-coated 96 wells (2000 cells per well) in low-glucose DMEM containing 10% FBS. 1200 cells/well was set up for proliferation assay in low-glucose media containing 0.5% FBS. Data were collected at day 4 or 5. HUVEC (p7-10) were grown on gelatin-coated plate until it reached 70–80% confluency.

On the day of the assay, cells were dissociated with 0.05% trypsin, which was neutralized with 0.5% FBS-containing EBM. Cells were briefly spun and then re-suspended in 0.5% FBS-media. Cells were counted and plated in 96-well, 1000 cells/well. Triplicate wells were used for each treatment. Data were collected at day 3, and then cells were fixed in 4% paraformaldehyde for 15 min before adding crystal violet. Cell-covered areas were quantified after taking pictures by Image J software.

Proliferation assays with fibroblasts were done in low-glucose DMEM containing 1% FBS, with or without 10 ng/ml bFGF, or 100 ng/ml human EGF and the assay was ended at day 3. ARPE-19 cells were gently lifted with 0.025% trypsin and plated in RtEGM media (Clonetics) containing 2% FBS, L-glutamine, human bFGF, and GA-1000. Once cells were attached to plates, serum-free RtEGM media was used to maintain the cultures. For proliferation assays, 1500 human RPE cells were plated into 96-well plates in low-glucose DMEM containing 1% FBS. A673, U87MG, Calu6, and AML12 cells were grown until confluent and were then harvested and re-suspended in appropriate assay media. For proliferation assays, cells were plated at the density of 1000–2000 cells/well in low-glucose DMEM containing 5% FBS or otherwise stated. Bovine pituitary cells (pituitary folliculostellate cells) were isolated as previously described^67^. For proliferation assays with human epidermal keratinocytes, human DMVECs, and human dermal fibroblast cells, 1000 cells/well were plated in low-glucose DMEM containing 1% FBS with or without various growth factors. The assay ended at day 3 for bovine pituitary cells and at day 4 for all the other cell types. For 4T1, 1000 cells were plated in RPMI-1640 with 2% basement membrane extract (BME) and 2% FBS on BME-coated 96 wells and treated 4 h later^68^. Four days later, tumor cell growth was measured by the MTS assay (Promega, Madison, WI), a colorimetric assay that measures metabolic activity of viable cells. Recombinant human transferrin was obtained from EMD Millipore (Temecula, CA). Recombinant mouse apo-transferrin was obtained from Sigma.

### SiRNA knowckdown

BCECs were plated in 6-well culture plates at a density of 1.5 × 10^5^ cells/well and cultured overnight. Two milliliters of antibiotics-free culture medium was used to replace the old medium. siRNAs, including siNegative (Ambion, AM4611), siRNA against JNK1#2 (Invitrogen, NM_001192974.2_siRNA_266), JNK1#4 (Invitrogen, NM_001192974.2_siRNA_485), siRNA against JNK2#2 (Invitrogen, XM_005208371.4_siRNA_1240), and JNK2#4 (Invitrogen, XM_005208371.4_siRNA_696), were mixed with Lipofectamine RNAiMAX reagent (ThermoFisher Scientific, 13778150) in Opti-MEM™ I Reduced Serum Medium (Gibco, 31985062) according to manufacturer’s instructions. Briefly, a mix containing 25 pmol of siRNA, 7.5 μl of RNAiMAX reagent, and 125 μl of Opti-MEM medium was used to transfect cells in each well, to a final siRNA concentration of 12.5 nM. A mix of RNAiMAX and Opti-MEM was used as no siRNA control. Cells were incubated with siRNAs. Eight hours later, the siRNA-containing medium was replaced with fresh medium. Twenty-four and/or 48 h after transfection with siRNAs, cells were used for proliferation assays and protein extraction.

### PNGase F treatment

Glycerol-free PNGase F was obtained from New England Biolabs (Ipswich, MA). Briefly, BCECs were lysed with NP-40 containing proteinase inhibitors (Thermo Scientific, Waltham, MA). Lysates were cleared at 4 °C at 5000 × *g* for 25 min. Total protein content was measured using Pierce BCA protein assay kit (Thermo Scientific). Twenty milligrams of protein was mixed with 10X denaturing buffer and H_2_O to a total volume of 10 ml. Glycoproteins were denatured at 100 °C for 10 min, followed by adding Glycobuffer and PNGase F. The reaction was carried out at 37 °C for 2 h.

### Western blots

Cells were allowed to reach ~80% confluency in 12-well plates. Cells were pre-treated with ManN, Kif, or Cas for various time durations, with or without the subsequent addition of VEGF, with H_2_O as the solvent control for ManN. At various time points, plates were taken out of the incubator and kept on ice. Cell monolayers were first washed once with ice-cold PBS before lysis with 250 μl of Pierce RIPA buffer (ThermoFisher Scientific, Rockford, IL) or use 50 mM Tris–HCl (pH 7.6), 150 mM NaCl, 10% Glycerol, 1% NP-40 containing protease/Phosphatase inhibitor cocktail (100X) (Cell Signaling, #5872). Lysates were collected and mixed with 4X Bolt LDS Sample Buffer (Novex, Carlsbad, CA) in the presence of Halt protease inhibitors and phosphatase inhibitor cocktail (ThermoFisher scientific, #NP0007). The samples were subjected to SDS-PAGE (Bolt 4–12% Bis-Tris Plus, Invitrogen) using Bolt MES SDS running buffer or NuPAGE 3–8% Tris-Acetate gel using Tris-Acetate SDS running buffer (Novex). HUVECs (passage 6–8) were plated in EBM-2 basal medium (Lonza) with 0.2% FBS. Following overnight culture, cells were serum-starved in EBM-2 medium for 4 h prior to treatment with 50 ng/ml of VEGF_165_ or vehicle controls for various lengths. Equal amounts of protein lysates were analyzed by SDS-PAGE and blotted with the indicated antibodies. Proteins were transferred using Tris-Glycine buffer with 20% Methanol (Proteonomics grade) (Apex BioResearch Products). Membranes were first incubated with 5% milk in TBST, pH 7.6 (TEKnova, Hollister, CA), followed by blotting with primary and secondary antibodies. ECL anti-rabbit IgG, horseradish peroxidase linked whole antibody from donkey or sheep anti-mouse were obtained from GE Healthcare (UK limited). SuperSignal West Dura Extended Duration substrate was from ThermoFisher Scientific. In some cases, the same PVDF membranes were stripped by 8-min incubation in the Restore Plus Western Blot Stripping Buffer (ThermoFisher Scientific) to show total specific protein expression, followed by second stripping for β-actin expression.

### Migration assays

HUVECs (passage 6–8) were cultured and serum-starved as described in “Western blots”. Ten thousand cells in 150 µl of EBM-2 medium were then added to the upper chamber of 8 µm pore size cell culture inserts (Falcon) coated with 0.1% gelatin. The lower compartment was filled with 600 µl EBM-2 medium containing various agents. The plates were incubated at 37 °C to allow migration. After 4 h, cells were fixed with 4% PFA for 20 min and then stained with crystal violet (Sigma-Aldrich) for 20 min at RT. Migrated cells on the bottom side of the insert membrane were quantified by counting whole area of the insert at ×40 magnification. The experiments were carried out in triplicate and repeated three times. BCEC migration was set up similarly, except that wells were coated with FN, cells were suspended in 1% serum media, and migration time was 18–24 h.

### Scratch assay

BCECs (passage 6–10) and HUVEC (passage 6–8) were used in this assay. Cells were grown until about 80% confluency in 6-well plates, washed twice with PBS, and then starved in serum-free DMEM (low Glucose, Hyclone) for 5 h before making a “scratch” using 1 ml tip. Cell monolayers were briefly washed once with serum-free media, followed by various treatments in media containing 1% FBS. Forty-eight hours later, the assay was stopped by adding 2 ml 4% paraformaldehyde. Twenty minutes later, fixed cells were stained with 1 ml Crystal Violet (Sigma). Plates were washed gently under the running tap water and air-dried before taking pictures. Images were acquired by ZEISS Discovery V8 SteREO microscopy equipped with PixeLINK Megapixel FireWire camera. Quantification of wound closure was done using AxioVision LE Rel.4.4 software. Six images were taken for each sample and six measurements (in pixel) were made on each image using AxioVision LE Rel.4.4 software.

### N-Glycan, monosaccharide, sialic acid, O-glycan analysis

As soon as BCECs reached about 80% confluency, they were washed twice with phosphate-buffered saline (PBS, Sigma) and then harvested by scraping. The cells were pelleted by centrifugation at 300 × *g* for 3 min and washed once with cold PBS. Cells were homogenized and total protein was measured. All subsequent analysis was based on known protein amount.

N-linked glycans were removed from glycoprotein samples using PNGase-F kit (New England BioLabs, P0705S). Briefly, 300 µg of protein sample was reconstituted in 180 µl UltraPure™ water. Twenty microliters of 10X denaturing buffer was added and boiled using 100 °C water bath for 14 min. Samples were cooled down to room temperature and centrifuged at 2700 × *g* for 1 min. Subsequently, 50 μl 10X NP-40 was added and samples were kept at room temperature for 30 min with vortexing at 5 min interval, followed by adding 25 μL of 10X reaction buffer and mixing thoroughly. Five microliters of PNGase F (2500 U) was then added to the samples and mixed gently. Samples were incubated at 37 °C for 16 h. Released N-glycans were purified using solid-phase extraction method. Briefly, N-glycans were purified by passing the reaction mixture sequentially over pre-conditioned Sep-Pak C18 1cc cartridge (Waters) and HyperSep PGC (poly graphitized charcoal) cartridge (25 mg, 1 ml Thermo Scientific). The cartridge was washed with 4 ml of water and the PGC alone was washed with additional 1 ml of water. N-glycans bound to PGC were eluted using 30% acetonitrile containing 0.1% TFA in water. Finally, purified N-glycans were lyophilized and labeled with 2-AB. Briefly, samples were dissolved in 10 μl solution of 0.44 M 2-AB (2-Amino benzamide) in 35% acetic acid in DMSO containing 1 M sodium cyanoborohydride. The samples were incubated at 65 °C for 2.5 h. The 2-AB labeled glycans were purified using GlycoClean S cartridge (GLYKO) following their glycan clean-up protocol. Excess reagent was removed from the samples using Glycoclean S-cartridge (Prozyme) and labeled glycans were dried using Speed Vac® and stored at −20 °C. Profiling of 2-AB labeled glycans was obtained using Dionex CarboPac^®^ PA1 (4 × 250 mm) anion exchange column along with a guard column (4 × 50 mm) at flow rate of 1 ml/min. Glycans were separated in 100 mM sodium hydroxide with a sodium acetate gradient of 0–250 mM in 0–75 min. The data were collected using the Dionex ICS-3000 HPLC system with Ultimate 3000 fluorescence detector (Dionex) set at *λ*_ex_ 330 nm at *λ*_em_ 420 nm with sensitivity 7. The data were processed using Chromeleon™ software (Thermo Scientific).

Monosaccharide composition analysis was done using HPAEC-PAD^69^ (Thermo-Dionex ICS-3000) and nmole amount of each monosaccharide present in 25 μg of protein was calculated. Samples were hydrolyzed using 2N trifluoroacetic acid (TFA) at 100 °C for 4 h. Followed by removal of acid using dry nitrogen flush. To ensure complete removal of acid, samples were co-evaporated twice with 100 μl of 50% isopropyl alcohol (IPA). Finally, the samples were dissolved in Milli-Q water and injected on HPAEC-PAD. Monosaccharide profile was done using Dionex CarboPac^™^ PA1 column (250 mm × 4 mm; with 50 mm × 4 mm guard column). An isocratic solvent mixture of 19 mM sodium hydroxide with 0.95 mM sodium acetate was used at a flow rate of 1 ml per minute for 25 min. Data were acquired using manufacture supplied standard Quad waveform for carbohydrates. All neutral and amino sugars were identified and quantified by comparing with authentic monosaccharide standard mixture consisting of L-fucose, D-galactosamine, D-glucosamine, D-galactose, D-glucose, and D-mannose^70^.

Mild acid hydrolysis was used to release sialic acid. Briefly, samples were treated with 2 M acetic acid at 80 °C for 3 h followed by removal of excess acid using speed vacuum. Sialic acid was then tagged with DMB reagent and analysis was done using RP-UPLC-FL (Waters Acquity UPLC) system. Known amount of standard Neu5Ac was used to quantify amount of sialic acid in samples.

For O-glycan analysis, homogenized cell samples were treated with 50 mM NaOH in presence of 1 M NaBH_4_ for 16 h at 45 °C. The reaction mixture was neutralized using ice-cold 30% acetic acid slowly. The neutratized reaction mixture was then passed over Dowex 50-X cation exchange resin to remove sodium ion and lyophilized. Excess boric acid generated during neutralization was then removed by co-evaporation using acidified methanol and methanol, respectively. Finally, the O-glycan was purified by passing over C18 cartridge. Dried and purified O-glycan was then methylated and used for O-glycan analysis after permethylation. Permethylated samples were then dissolved in absolute methanol and mixed with SDHB (Super-DHB) MALDI matrix in 1:1 v/v ratio and spotted on maldi plate. Mass spectral data were acquired using Bruker AutoFlex mass spectrometer at positive, reflectron mode. The mass spectral data were analyzed and annotated using GlycoWork Bench software and masses matched with the proposed structures were annotated. The mono-isotopic ion intensities are taken for calculation.

To measure cellular uptake of ManN and subsequent conversion to ManN-6P, BCECs were grown in 60-mm dishes to a density of ~6 × 10^5^ cells per dish. ManN was added to cultures at the final concentration of 400 μM. Cells were then incubated for 2 h. Monolayers were washed three times with PBS at room temperature and lifted by a cell scraper on ice in 10 ml PBS. Cell pellets were obtained by centrifugation at 400 × *g* for 5 min and stored at −80 °C for further use. Cell pellets were suspended in 200 µl of Ultrapure ice-cold water in presence of 1 µl of protease inhibitor. Cells were sonicated for 1 min with 30 s pulses and vortexed to form homogeneous solution. 2.5 µl of the homogenate was used for protein estimation using BCA-assay method in triplicate. A standard curve of BSA at concentration between 0 and 800 µg/ml was done to quantitate the total protein amount. The cell homogenate was filtered through pre-washed 3 K filters and the filtrate was dried using speed-vac. The dry sample was reconstituted in 100 μl of ultrapure water and sample with 200 μg equivalent of protein was injected onto HPAEC-PAD. A known amount (1 nmol) of ManN, Glucose, Mannose, and ManN-6P standards were used to quantify the sugars present in the samples. All standards, except ManNH_2_-6P, were obtained from Sigma-Aldrich. ManNH_2_-6P was from Omicron Biochemicals, Inc (South Bend, IN). The amounts of monosaccharides present in different cells are presented as nmol/mg of total protein amount. All analyses were performed in a Thermo-Dionex ICS system using a CarboPac-PA-1 column in 100 mM NaOH and 250 mM NaOAc as HPLC running buffer.

### Biotinylation of surface proteins

BCECs were plated in 10 cm cell culture dishes 3 days prior to the cell-surface protein isolation. Cells were washed three times with Dulbecco’s PBS with CaCl_2_ and MgCl_2_, followed by a 30 min incubation with EZ-Link Sulfo-NHS-SS-Biotin (Pierce, Rockford, IL, USA; 0.5 mg/ml in Dulbecco) on ice. Cells were washed twice with Dulbecco and the non-reacted biotin was blocked with 20 mM glycine for 15 min. To prevent the reduction of the disulfide bridge in the biotin molecule during the cell lysis process, a 100 μM oxidized glutathione (Sigma-Aldrich, St. Louis, MO) was added in the last wash solution. For cell lysis 500 μl of lysis buffer (2% NP-40, 1% Triton X-100, 10% glycerol, 100 μM oxidized glutathione, EDTA free protease inhibitor tablet (Roche, Mannheim, Germany) in PBS was added to the cells. Lysed cell extracts were scraped off the plates and transferred to an Eppendorf tube followed by incubation on ice on a shaker for 30 min. The cell extracts were incubated with 30 U of DNase (22 °C 50 min, Roche, Mannheim, Germany) and centrifuged for 20 min (20,800 × *g*, at 4 °C) to pellet the insoluble material. The protein concentration of the supernatants was determined. Equal amounts of protein (~2 mg) from each extract were used for cell-surface protein isolation. The supernatant was pre-cleared using biotin agarose beads (Pierce ImmunoPure® Immobilized D-biotin, Thermo Scientific, 20221) and pre-cleared solution was used for the cell-surface protein isolation using streptavidin beads. Beads were washed four times with the lysis buffer, four times with 300 mM NaCl in lysis buffer, and twice with 50 mM Tris–HCl, pH 7.8. Proteins were eluted twice with an elution buffer (50 mM DTT in 50 mM Tris–HCl, pH 7.8) at 30 °C, followed by pooling of the elutes. Three biological replicates and one non-biotinylated control were used in the study.

### Gene expression analysis by real-time Q PCR

RNA was prepared using the RNeasy Mini Kit (Qiagen). Fifty nanograms of total RNA per reaction was used for the real-time PCR (Taqman) analysis. Reactions were set up in MicroAmp Fast Optical 96-well reaction plate, seal with MicroAmp optical Adhesion film and run on ViiA7 Real-time PCR system (Applied Biosystems) and the absolute quantification with standard curve was used with Sequence Detection System (SDS) software. The expression level of each gene was further quantified relative to the housekeeping gene *RPL19* in the same sample. Taqman primers and probe mixes were obtained from ThermoFisher Scientific. Bovine *VEGF-A* (Bt03213282), bovine *RPL19* (Bt03229687) and bovine specific *VEGFR2* (Bt03258877), *GLUT1* (Bt03215313), and *GLUT4* (Bt03215316).

### α-Mannosidase, α- and β-glucosidase activity assays

α-mannosidase activity was measured using substrate *p*-nitrophenyl α-mannopyranoside (1 mM). Enzyme from Jack Bean (M7257) (final concentration of 0.077 U) was incubated at 37 °C in a final volume of 50 μl of 50 mM potassium phosphate buffer, pH 7.5. α-glucosidase was assayed with substrate *p*-nitrophenyl α-glucoside (7 mM). Enzyme from *Saccharomyces* *cerevisiae* type 1 (Sigma, G5003) (final concentration of 0.1 U) was incubated at 37 °C in a final volume of 50 μl of PBS, pH 7.5. β-Glucosidase was assayed with substrate 4-nitrophenyl β-D-glucopyranoside (Roche). Enzyme from almond (Sigma, G0395) (final concentration of 0.002 U) was incubated at 37 °C in a final volume of 50 μl of PBS, pH 7.5 containing 1% SDS. The incubation was stopped by addition of an equal volume of acid-based stop solution (R&D Systems, 895032). Enzymatic activity was measured at 405 nm. α-glucosidase from *Saccharomyces cerevisiae* type I (G5003) with *p*-nitrophenyl α-D-glucopyranoside (Sigma, N1377) as the substrate.

### Measurement of ManN in wound fluid from *S. aureus* infected mice

To test ManN stability in wound fluid collected from mice with skin infection as described below, 1.5 μl of 5% ManN solution was added to each 200 μl wound fluid which was first diluted 1:1 (v/v) with PBS. At each time point, samples were taken from 37 °C incubator and stored at −80 °C. Plasma proteins were precipitated by adding ice-cold acetonitrile to plasma: acetonitrile 1:3 (v/v) ratio. Samples were kept over ice for 1 h and then centrifuged at 12,000 × *g* for 10 min at 7 °C to form a pellet. Supernatants were transferred to other tubed, dried down on a Speed Vac® and then reconstituted in UltraPure™ distilled water and filtered through pre-washed Nanosep 3 K Omega filters (Pall Corporation). The filtrate was dried down on Speed Vac®. The dry samples were dissolved in 100 μl of water and 2 µl plasma or wound fluid sample was subjected to HPLC analysis. Neutral and amino sugars were separated on a Dionex CarboPac^™^ PA1 column 4 mm × 250 mm with 4 mm × 50 mm guard column. An isocratic gradient of 19 mM sodium hydroxide with 0.95 mM sodium acetate was used at a flow rate of 1 ml/min with 20 min run. Data were collected using the Dionex ICS-3000 HPLC system with pulsed amperometric detector using standard Quad waveform. ManN was identified and quantified by comparison with monosaccharide standard using Thermo Scientific™ Chromeleon™ software. No ManN samples served as negative controls.

### Skin wound healing model

All animal experimental procedures were approved by the Institutional Animal Care and Use Committee (IACUC) of the University of California San Diego and conducted in an ethical fashion and in accordance with the guidelines of the Animal Care Program (ACP).

The model has been previously described^38^. Briefly, C57BL/6 female mice (8–10 weeks old) were obtained from Jackson labs (Sacramento, CA). A fresh, full-thickness punch wound (4-mm diameter) using a punch (Acu Punch, Acuderm Inc., Ft. Lauderdale, FL) splinted with a sterile neoprene ring (6-mm outer diameter and 4-mm inner diameter), fastened with 5–6 sutures (4–0 nylon) under the influence of Isoflurane was created on the back of the animal in a Class II Biological Safety Cabinet. For all surgical procedures, sterile technique was followed. Buprenorphine was given subcutaneously prior to awakening from anesthesia for anticipated pain. Mice were monitored until fully awake and were housed individually to minimize damage/biting/fighting to the surgical site. Recombinant human VEGF was a gift from Roche-Genentech (Telbermin, recombinant human VEGF_165_). Treatment agents were prepared in PBS, sterile-filtered and 25 μl solution was applied daily directly to a wound bed for the first 4–5 days under the influence of Isoflurane, followed by daily observation. Wound closure was monitored by regular imaging and the wound area was quantified using ImageJ (National Institutes of Health, Bethesda, MD, USA).

At day 4 after wounding, wounds were excised with a 2-mm rim of surrounding tissue and placed in 10% formalin for a maximum of 24 h. The wounds were then bisected down the center, and 5-μm paraffin sections were processed for Hematoxylin and Eosin (H&E) and Masson’s Trichrome staining. Epithelial gap was measured histomorphometrically using AxioVision LE Rel.4.4 software. The skin tissues were fixed in 10% formalin for 24 h. Paraffin embedding and sectioning were performed by the UCSD, Moores Cancer Center Histology Core. The 5-μm paraffin sections were deparaffinized and rehydrated before heat-induced antigen retrieval was performed in 10 mM citrate buffer (pH 6.0). Immunostaining was performed as previously described^20^. Anti-CD31 (SZ31, rat IgG2a) (Dianova, Warburgstrasse 45, 20354 Hamburg, Germany) was used at 2 μg/ml. Small vessels stained positive for CD31 were counted microscopically on 10 fields (×20) taken around the wound.

### Vascular permeability assay

Vascular permeability was assessed using a modified Miles assay^14^. Hairless male guinea pigs (Crl: HA-Hrhr/IAF, 75 days old, 450–500 g, Charles River Laboratories) were anesthetized by intraperitoneal (i.p.) administration of xylazine (5 mg/kg) and ketamine (75 mg/kg). The animals then received an intravenous injection (penile vein) of 1 ml of 1% Evans blue dye. After 15 min, intradermal injections (0.05 ml/per site) of different doses of ManN were administrated into the area of trunk posterior to the shoulder. All reagents were diluted in PBS for intradermal administration. Twenty-five nanograms of VEGF_165_ per site was used as positive control. Thirty minutes after the intradermal injections, animals were euthanized by i.p. injection of pentobarbital (200 mg/kg). Skin tissues were dissected from the connective tissues and photographed.

### Murine skin infection model

The skin infection model employed in the present study was previously described^39^. Briefly, mid-log phase of *Staphylococcus aureus* sub-cultured from overnight cultures in Todd Hewitt broth were used in this study. Six- to eight-week-old C57BL/6 mice were obtained from Charles River Laboratories. Mice were shaved and depilated by Nair cream before infection. 5 × 10^7^ CFU of *S. aureus* was intradermally injected into the left groins of mice. After 3 days, abscesses were surgically removed and homogenized on ice. Fluid was collected and spun at 14,000 rpm. Cleared supernatant was diluted with PBS 1:1 for further use. Animals were housed in clean cages and experimental procedures hereafter were carried out under pathogen-free conditions. The presence of bacteria in the wound fluid was confirmed using Todd Hewitt Broth (THB) plates.

### Hindlimb ischemia model and evaluation of blood flow

C57BL/6 male mice (6–8 weeks old) were subjected to unilateral hindlimb surgery under anesthesia with ketamine/xylazine cocktail^41,43^. Briefly, the left femoral artery was separated from the vein and nerve, ligated proximally, and excised. The right hindlimb served as control. Blood flow was measured by using a laser Doppler perfusion imager (PeriScan PSI; Perimed). Ischemic and nonischemic limb perfusion was measured before and after surgery and 1, 2, and 3 weeks later. After surgery, mice were randomly allocated to different groups (8 mice for each group). Two hundred microliters of 20% ManN was orally administrated every other day from 3rd day after surgery. Two hundred microliters of 1 mg/ml Kif was administrated through i.p. injection every other day. H_2_O was used as a vehicle control. The final blood flow values were expressed as the ratio of ischemic to nonischemic hindlimb perfusion from the same animal. Quantification of blood vessel area was carried out as described^41,43^.

### Retinal neovascularization

Assessment of retinal angiogenesis following intravitreal administration was done as described^41^. Briefly, 6–8-week-old C57BL/6 male mice were randomly allocated to different groups and anesthetized with ketamine/xylazine cocktail. The indicated amounts of ManN, Kif, or bFGF (R&D Systems, AF-233-NA) in 1 μl of PBS and PBS vehicle control were injected intravitreally with a 33-gauge Hamilton syringe. Seven days after injection, animals were euthanized. Eyes were then enucleated and fixed in 4% paraformaldehyde (PFA) for 30 min. Retinas were separated and stained with anti-CD31 immunofluorescence (IF) to evidence the vasculature. Evaluations were performed by an investigator blinded to the treatment. For CD31 IF, rat anti-mouse antibody (BD Biosciences, CAT# 550274) was diluted 1:100 and incubated overnight at 4 °C. After 4-h incubation with the Alexa Fluor-488-conjugated anti-rat antibody (Life Technologies, A11006), whole mounts were imaged via the 488-nm channel using A1R Confocal STORM super-resolution system (Nikon). Quantification of vascular density in choroids and retina was carried out by ImageJ. Each experiment was repeated three times with similar results, and each treatment group consists of five individual samples.

### Statistical significance and reproducibility

Statistical parameters including the *n* values, are indicated in the figure legends. The sample size was determined to ensure adequate power, as recommended by the Biostatistics and Bioinformatics Department, Moores Cancer Center. We used two-tailed, two-sample unequal variance *t* test. Statistical significance was further confirmed using Wilcoxon rank-sum test between treatment groups of interest for some of the in vitro data sets as the method does not need the normal assumption on variables. Statistical inference was based on the *p*-value of each comparison using R function “wilcox.test”. We used linear mixed-effects (LME) model to investigate the wound area (in percentage), between three treatment groups (ManN, VEGF, VEGF + ManN) and PBS group. Two LME models were fitted. In the first LME model, the controlled group was considered as the reference group. We included day effect (considered days as categorical variable instead of continuous variable), and its interaction with treatment as fixed effects, and we included subject id as the random effect to involve correlations among measurements on different days for the same subject. There’s no difference among different groups at baseline. In the second LME model, we relevel group VEGF + ManN as the reference group in order to investigate comparisons between single-treatment groups and combination treatment. For each LME model, we explored different treatment effects and their corresponding *p* values on day 3, day 5, and day 8 with respect to the reference group, respectively. Data are considered significant when *p* < 0.05. Significant *p* values are represented in the figures as follows: ***p < 0.001, **p < 0.01, *p < 0.05. For each experiment, a representative experimental result is shown from two to five independent studies.

### Reporting summary

Further information on research design is available in the Nature Research Reporting Summary linked to this article.

## Supplementary information

Supplementary Information

Reporting Summary

## Source data

Source Data

## Data Availability

All relevant materials are available from the authors. No genomic or microarray data sets were generated in this study. Source data are provided with this paper.
