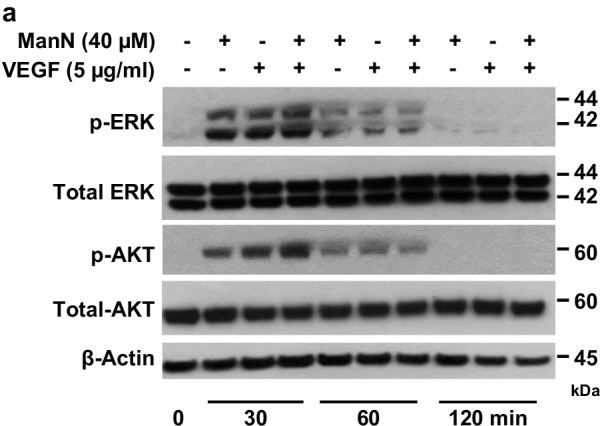# Author Correction: Inhibition of protein glycosylation is a novel pro-angiogenic strategy that acts via activation of stress pathways

**DOI:** 10.1038/s41467-025-67407-y

**Published:** 2025-12-08

**Authors:** Cuiling Zhong, Pin Li, Sulabha Argade, Lixian Liu, Anastasia Chillá, Wei Liang, Hong Xin, Brian Eliceiri, Biswa Choudhury, Napoleone Ferrara

**Affiliations:** 1 Department of Pathology, University of California, San Diego, La Jolla, CA USA; 2 Department of GlycoAnalytics Core, University of California, San Diego, La Jolla, CA USA; 3 Department of Surgery, University of California, San Diego, La Jolla, CA USA

Correction to: *Nature Communications* 10.1038/s41467-020-20108-0, published online 10 December 2020

In the version of the article initially published, the “Total-AKT” image in Fig. 2a was incorrect due to an error in figure preparation. Figure 2a has now been corrected in the HTML and PDF versions of the article, as shown below.

Original Fig. 2a
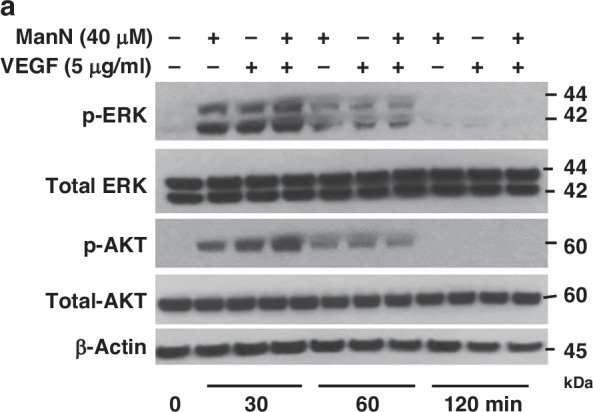


Corrected Fig. 2a