# Supplementary material for: Inhibition of protein glycosylation is a novel pro-angiogenic strategy that acts via activation of stress pathways
Source: Nat Commun. 2020 Dec 10;11:6330. doi: 10.1038/s41467-020-20108-0 (PMC7730427; doi:10.1038/s41467-020-20108-0)
Supplement: Supplementary file 1 — Supplementary Information [file 41467_2020_20108_MOESM1_ESM.pdf]

Supplementary Information

Supplementary Figure 1- 12

Supplementary Table 1-3

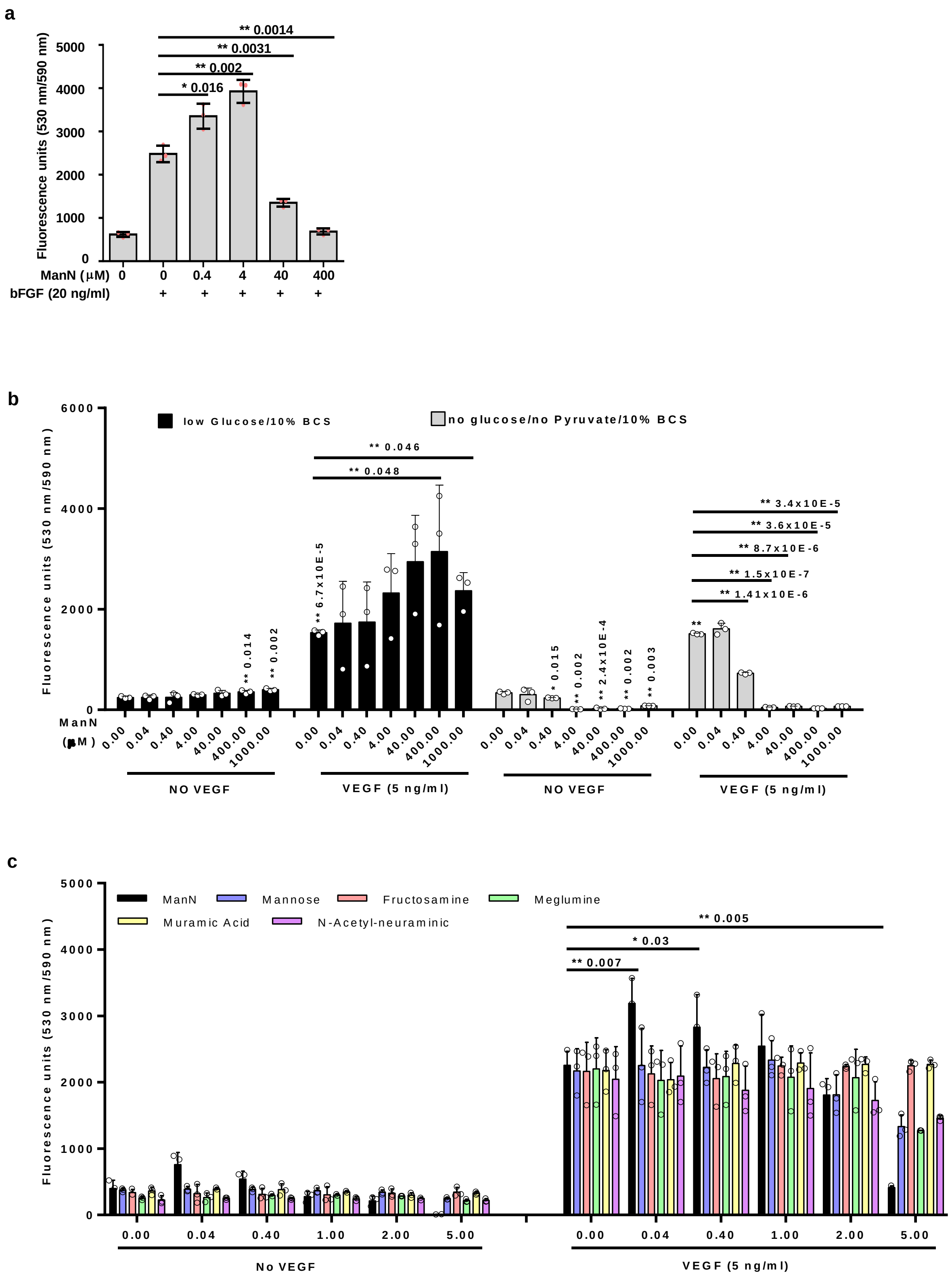
a
** 0.0014
** 0.0031
** 0.002
* 0.016
Fluorescence units (530 nm/590 nm)
ManN (μM) 0 0 0.4 4 40 400
bFGF (20 ng/ml) + + + + +
b
low Glucose/10% BCS
no glucose/no Pyruvate/10% BCS
** 0.046
** 0.048
** 6.7x10E-5
** 0.014
** 0.002
* 0.015
** 0.002
** 2.4x10E-4
** 0.002
** 0.003
** 3.4x10E-5
** 3.6x10E-5
** 8.7x10E-6
** 1.5x10E-7
** 1.41x10E-6
Fluorescence units (530 nm/590 nm)
ManN (μM)
NO VEGF
VEGF (5 ng/ml)
NO VEGF
VEGF (5 ng/ml)
c
ManN
Mannose
Fructosamine
Meglumine
Muramic Acid
N-Acetyl-neuraminic
** 0.005
* 0.03
** 0.007
Fluorescence units (530 nm/590 nm)
No VEGF
VEGF (5 ng/ml)

Supplementary Fig 1. ManN, but not structurally-related molecules, stimulates endothelial cell proliferation. (a) Additive effects of ManN and bFGF on BCEC proliferation. Bell-shaped effects of ManN on BCEC proliferation. BCECs were treated with ManN ranging from 0.4-400 µM for 5-6 days, with or without 20 ng/ml bFGF. At the end of the experiment, proliferation was quantified using AlamarBlue®. (b) Additive effects of VEGF and ManN on BCEC proliferation are dependent on glycolysis. Proliferation assays were carried out in low glucose DMEM without growth factors or in DMEM without glucose and pyruvate. Asterisks indicate a significant difference compared with no treatment control. Statistical analysis was also done to compare VEGF alone and VEGF plus ManN treatment groups for cells grown in two different assay media. (c) BCECs were treated with various agents at 0.04 µM-5 mM in the absence or presence of 5 ng/ml VEGF. n=3 independent samples. For each study, a representative experiment is shown from 2 independent studies. Data are means +/- SD. Statistical analysis was done by 2-tailed, two-sample unequal variance *t* test. * $p<0.05$, ** $p<0.01$. Data are provided as a Source Data file.

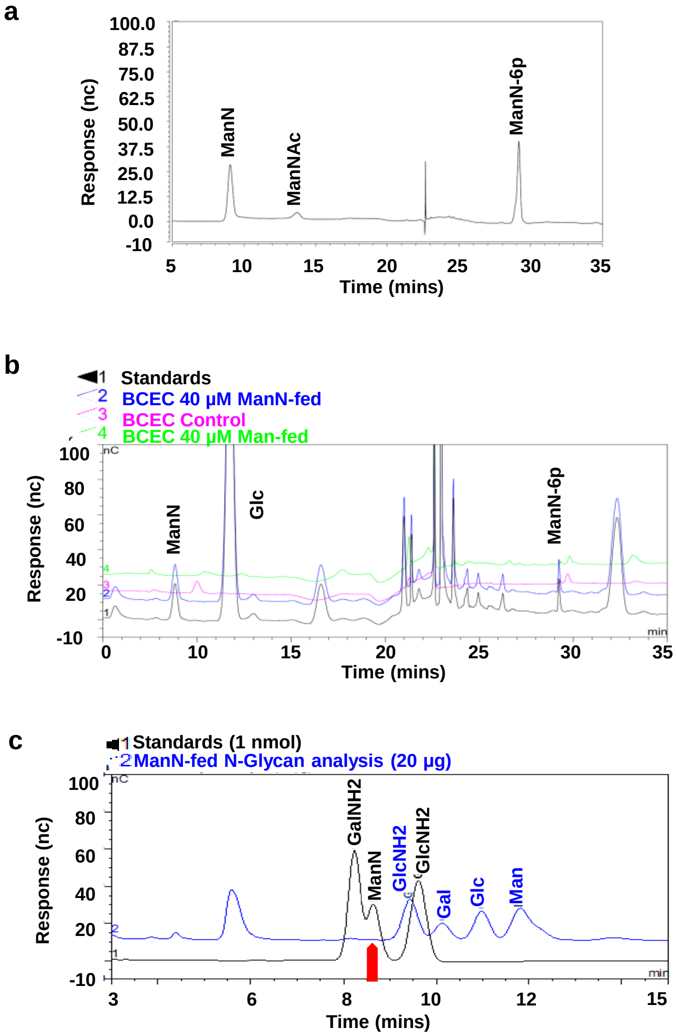

Supplementary Fig 2. N-glycan and monosaccharide analysis. (a) Chromatogram illustrating the separation of ManN, ManNAc and ManN-6-phosphate (ManN-6p) on a Dionex CarboPac™ PA1 HPLC column as described in Methods. (b) Chromatogram of monosaccharide composition assays. BCECs were untreated (Control) or treated with 40 μM mannose or ManN for 24 hrs. N-glycans hydrolyzed to monosaccharide are profiled using HPAEC-PAD. Peaks labeled were Glc (glucose), ManN and ManN-6p. (c) ManN (indicated by red arrow) is not incorporated in N-glycans. N-glycans isolated from cells treated with 40 μM ManN for 24 hrs, as shown from a

representative chromatogram. Peaks labeled were monosaccharides detected in ManN-treated samples: Man (mannose); Glc (glucose); Gal (galactose) and GlcNH2 (glucosamine). Standards for ManN, ManN-6p, GlcNH2 and GalNH2 (galactosamine) were loaded at 1 nmol. nC: nano-Coulomb.

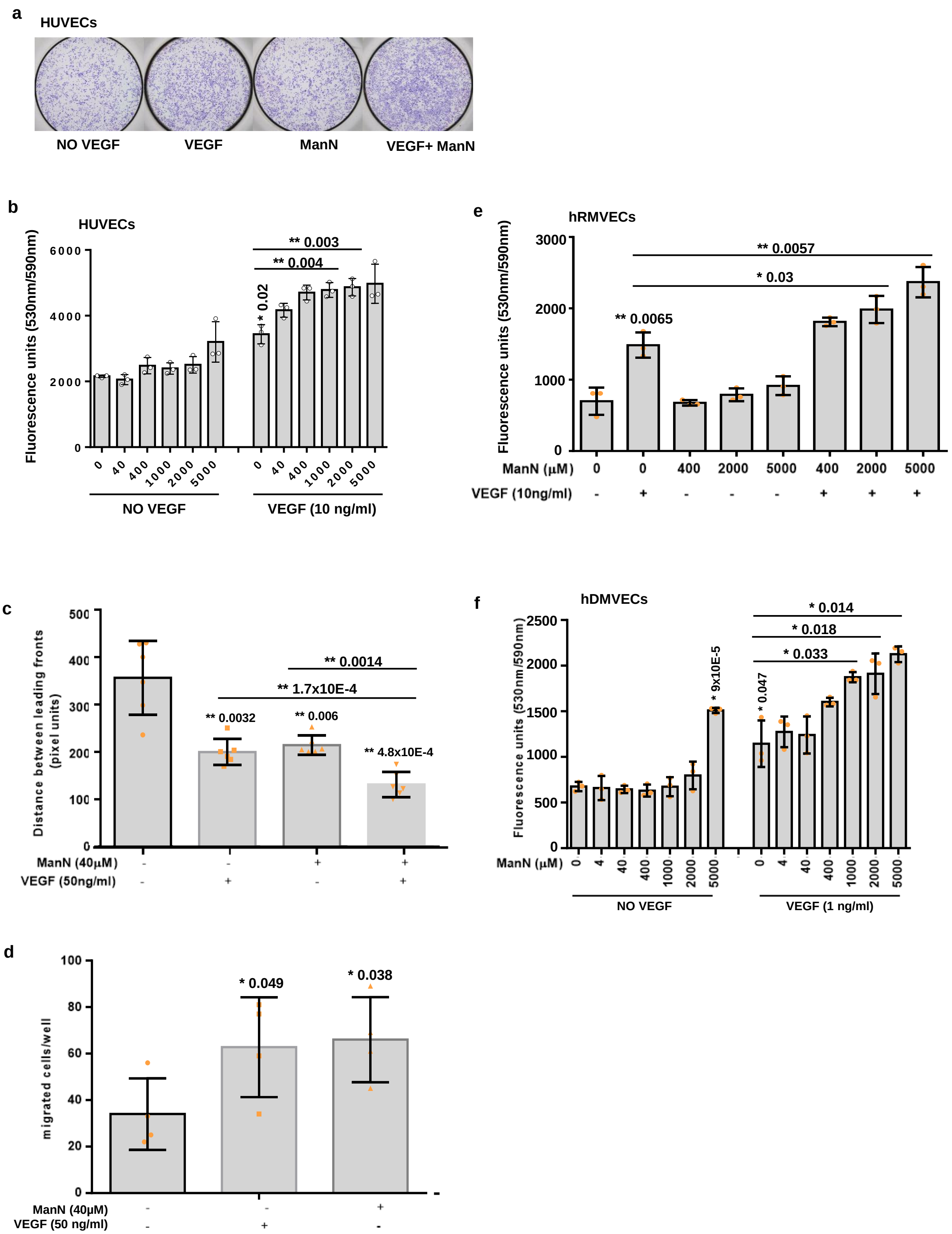
a
HUVECs
NO VEGF
VEGF
ManN
VEGF+ ManN
b
HUVECs
Fluorescence units (530nm/590nm)
6000
4000
2000
0
** 0.003
** 0.004
* 0.02
0
40
400
1000
2000
5000
NO VEGF
VEGF (10 ng/ml)
e
hRMVECs
Fluorescence units (530nm/590nm)
3000
2000
1000
0
** 0.0057
* 0.03
** 0.0065
ManN (µM)
VEGF (10ng/ml)
c
Distance between leading fronts (pixel units)
500
400
300
200
100
0
** 0.0014
** 1.7x10E-4
** 0.0032
** 0.006
** 4.8x10E-4
ManN (40µM)
VEGF (50ng/ml)
f
hDMVECs
Fluorescence units (530nm/590nm)
2500
2000
1500
1000
500
0
* 0.014
* 0.018
* 0.033
* 9x10E-5
* 0.047
ManN (µM)
0
4
40
400
1000
2000
5000
NO VEGF
VEGF (1 ng/ml)
d
migrated cells/well
100
80
60
40
20
0
* 0.049
* 0.038
ManN (40µM)
VEGF (50 ng/ml)

Supplementary Fig 3. ManN has mitogenic effects in human endothelial cells. (a) Dose-dependent effects of ManN on HUVEC proliferation. HUVECs (passage< 8, 1200 cells/well) were plated in 96-well plates in EBM containing 0.5% FBS. Cells were treated with ManN at concentrations ranging from 40 μM-5 mM for 3-4 days, with or without 10 ng/ml VEGF. At the end of the study, cells were fixed and stained with crystal violet. Each dose was tested in triplicate. A representative experiment is shown testing VEGF at 10 ng/ml and ManN at 40 μM. (Bb Quantification of HUVEC proliferation. Each dose group was tested in triplicate, with H2O as control. Cell numbers were quantified by adding AlamarBlue®. Fluorescence was measured at 530 nm/590 nm. (c) HUVEC confluent monolayers grown in 6-well plates were scratched with 1ml pipet tip, washed and then treated for 40 hrs in EBM-2 media containing 1% FBS. Each group was tested in triplicate wells. Images were taken and gaps between leading wound front were quantified using AxioVision LE Rel.4.4 software. Representative images from crystal violet staining are shown. (d) Effects of ManN on HUVEC transwell migration, with 20 ng/ml VEGF as a positive control. Transwells were first coated with gelatin and HUVECs, with or without various treatments, were allowed to migrate for 20 hrs in EBM-2 media containing 1% FBS. Each dose was tested in quadruplicate. Dose-dependent effects of ManN on hRMVEC (e) and hDMVEC (f) proliferation in the presence or absence of 1 or 10 ng/ml VEGF. n=3 independent samples. Asterisks indicate a significant difference compared with control. When statistical analysis was done using a different control, a line was used between specific groups. Data were means +/- SD. Statistical analysis was done by 2-tailed, two-sample unequal variance *t* test. * $p<0.05$, ** $p<0.01$. Data are provided as a Source Data file.

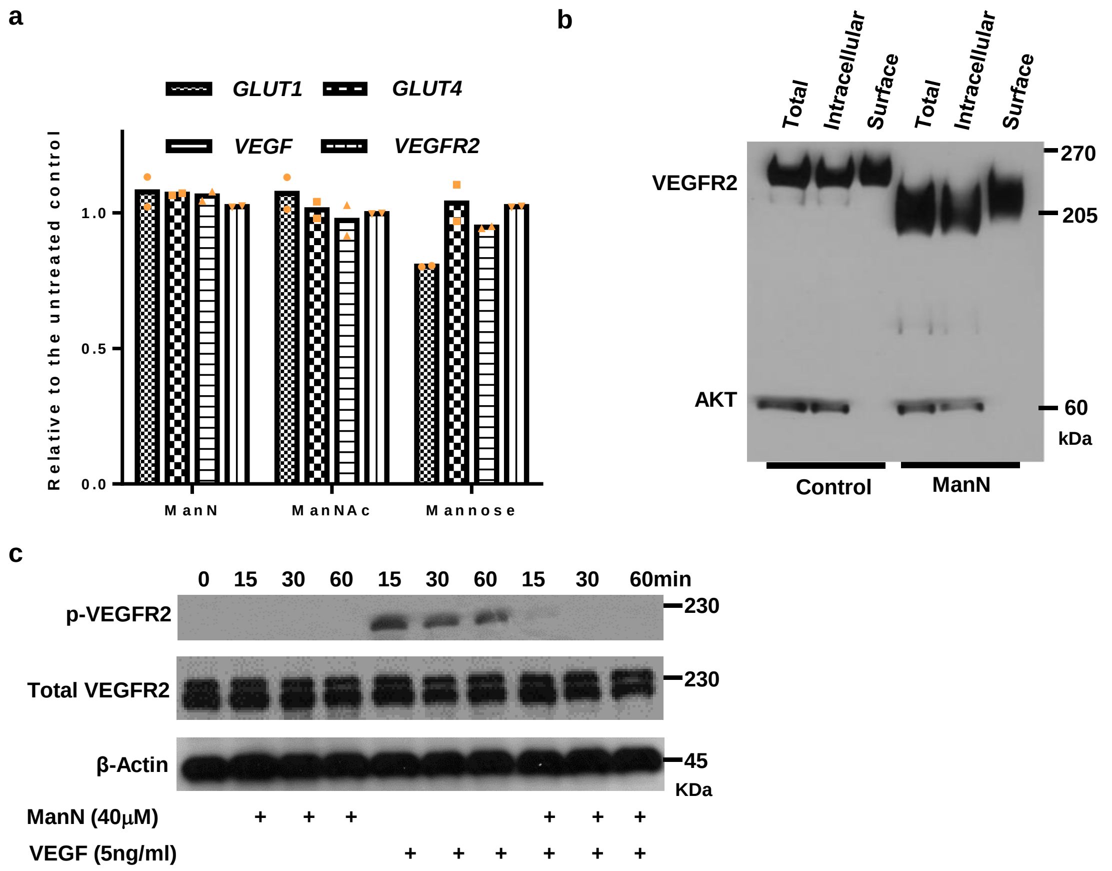

Supplementary Fig 4. Effects of ManN on VEGFR2 transcription, surface expression and signaling. (a) No significant increases in *VEGF, VEGFR2, Glut-1* and *Glut-4* transcription upon ManN treatment in BCECs. BCECs were treated with 40 μM of ManN, ManNAc and Mannose for 4 hrs. Gene expression analysis was done by real-time Q-PCR. An average is shown. n=2 independent samples. (b) Surface VEGFR2 expression. BCECs, untreated or treated with 400 μM ManN, were first biotinylated, lysed and then subjected to western blot analysis for VEGFR2. AKT served as a control. (c) Time course of VEGFR2 activation. Serum-starved BCECs were stimulated for various time durations with 40 μM ManN or 5 ng/ml VEGF. The same amounts of total protein were loaded at each point. Total and phosphorylated VEGFR2 (Tyr 1175) were examined. Data are provided as a Source Data file.

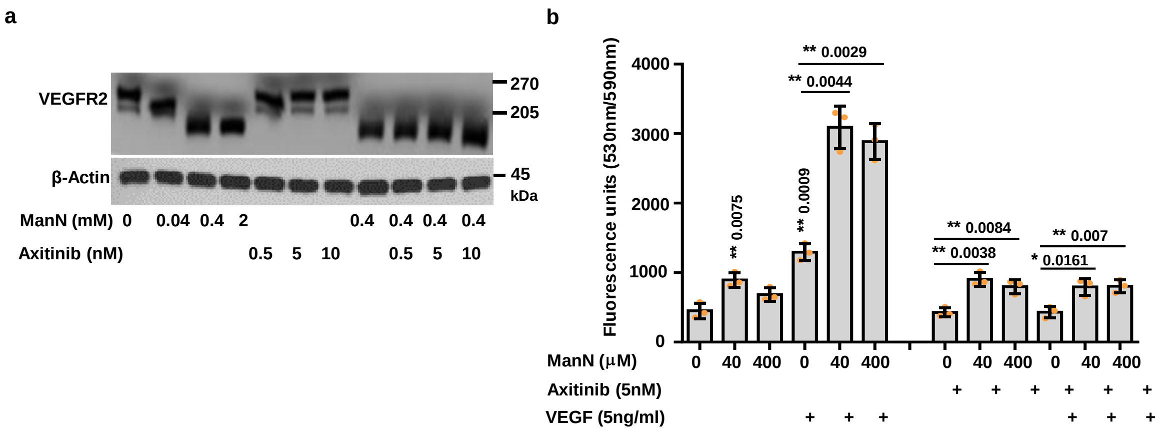

Supplementary Fig 5. Effects of ManN on BCEC proliferation and glycosylation are VEGFR2-independent. (a) BCECs were treated with ManN at 0.04-2 mM, axitinib at 0.5-10 nM or a combination of ManN at 0.4 mM together with various concentrations of axitinib for 24 hrs. VEGFR2 western was performed. Axitinib, tested up to 10 nM, did not reverse the effects of ManN on VEGFR2 molecular mass. (b) In the presence of 5 nM axitinib, additivity between ManN and VEGF was abolished, while the effects of ManN alone on BCEC proliferation were not affected. n=3 independent samples. Data were means +/- SD. Statistical analysis was done by 2-tailed, two-sample unequal variance *t* test. * $p<0.05$, ** $p<0.01$. A representative experiment is shown from 2 independent studies. Data are provided as a Source Data file.

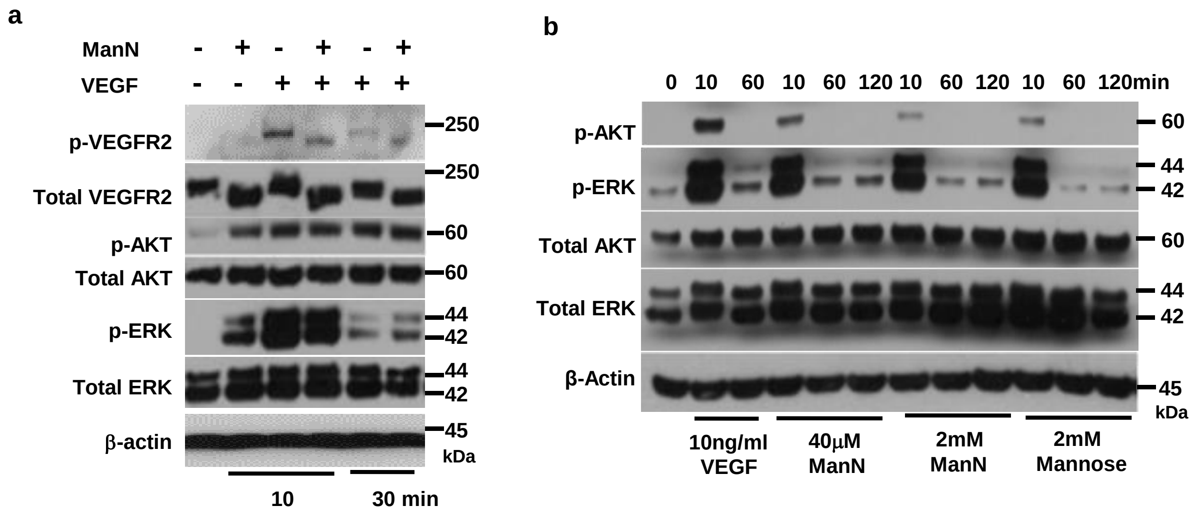

Supplementary Fig 6. ManN stimulates ERK and AKT phosphorylation in HUVECs. (a) Cells were pre-treated with 40 μM ManN for 24 hrs, followed by stimulation with 10 ng/ml VEGF for 10 or 30 mins. (b) Time-course activation of ERK and AKT in HUVECs by 40 μM and 2 mM ManN or 2 mM mannose. β-actin served as the loading control for all western blot analyses. A representative experiment is shown from 2 independent studies. Data are provided as a Source Data file.

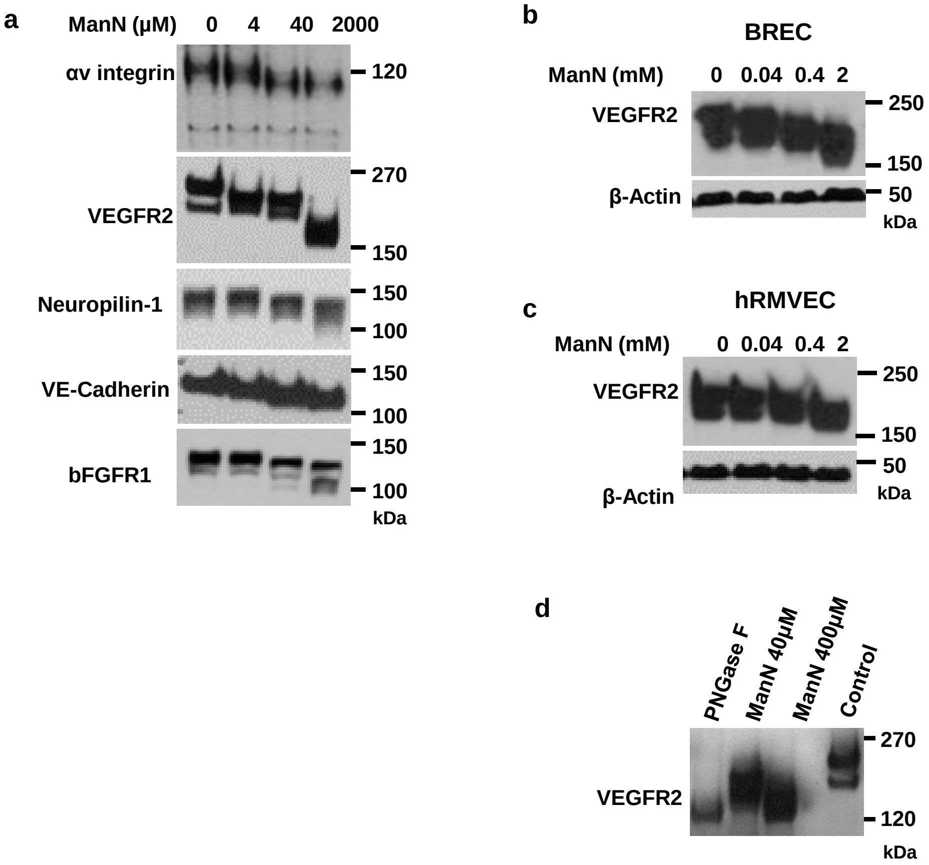

Supplementary Fig 7. ManN administration induces molecular mass changes in multiple cell signaling and adhesion molecules. (a) Cells were treated with ManN at various concentrations for 24 hrs. Proteins were separated either on 4-12 % Bis-Tris gel (for αv integrin, neuropilin-1, VE-cadherin and bFGFR1) or on 3-8 % Tris-Acetate gel (for VEGFR2) and western blot analyses were then performed. Dose-dependent shifts of VEGFR2 by ManN are shown in BRECs (b) and hRMVECs (c). β-actin served as the loading control. (d) BCECs were treated with PNGase F for 2 hrs or ManN at 40 μM and 400 μM for 24 hrs at 37ºC. VEGFR2 western blot analysis was performed. A representative experiment is shown from 2 independent studies. Data are provided as a Source Data file.

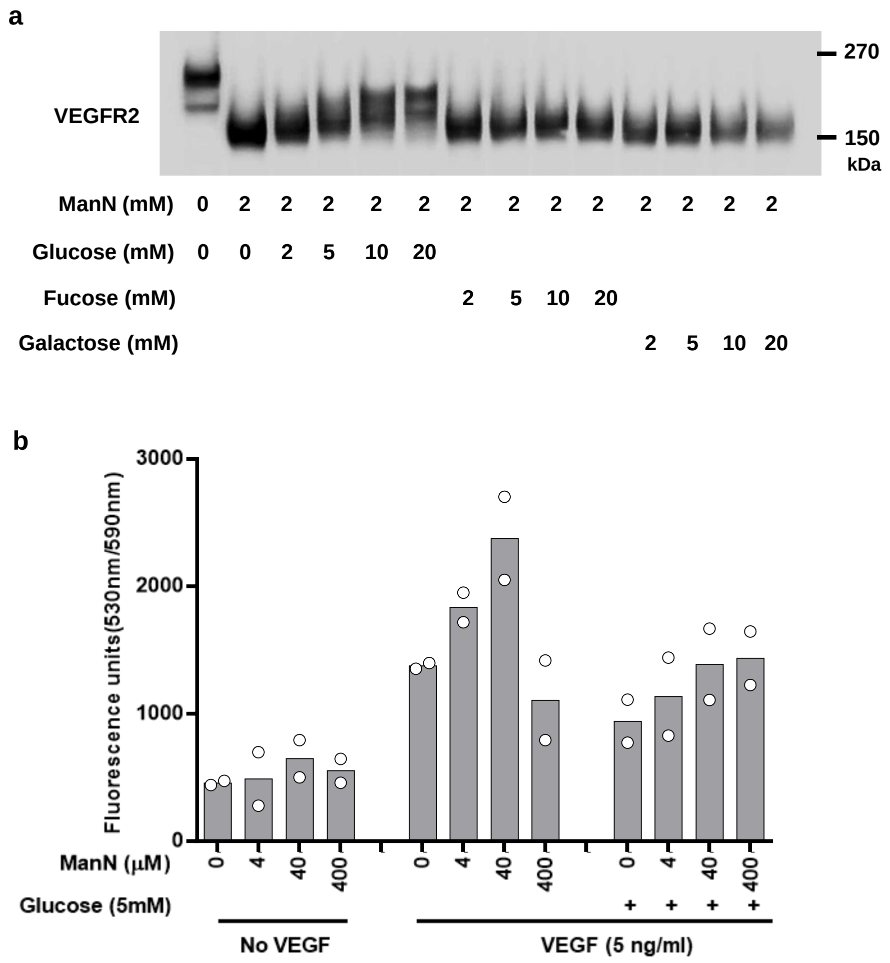

Supplementary Fig 8. Glucose, but not fucose or galactose, reversed the effects of ManN on VEGFR2 molecular mass change. (a) BCECs were treated with ManN at 2 mM for 24 hrs in the absence or presence of glucose, fucose or galactose at 2-20 mM. VEGFR2 western blot analysis was performed. (b) BCECs were allowed to attach before adding ManN at 4-400 μM. Two hours later, cells were treated with 5 mM glucose, with or without 5 ng/ml VEGF. Six days later, cell proliferation was quantified using AlamarBlue®. An average was shown from two independent samples. Data are provided as a Source Data file.

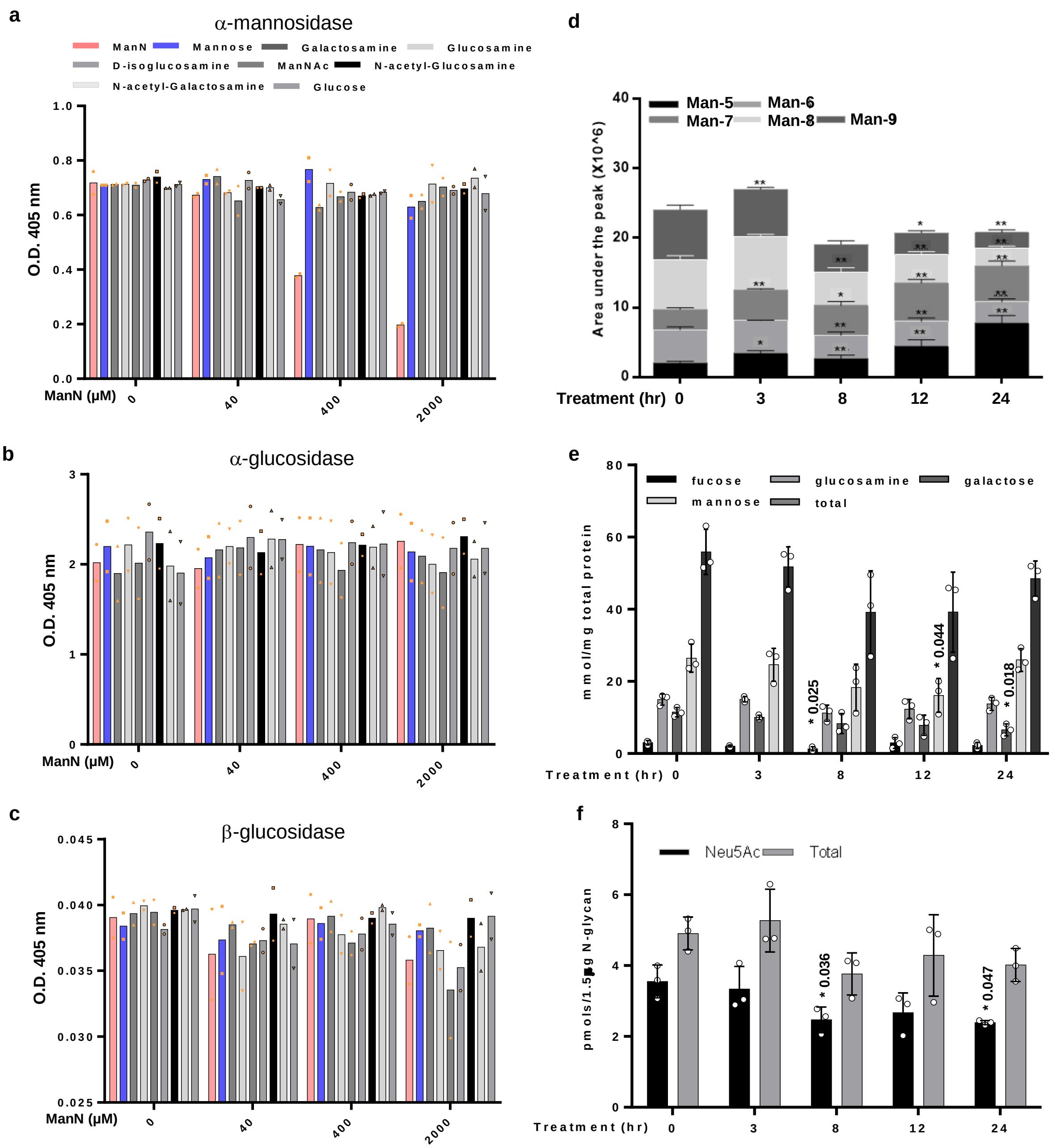

Supplementary Fig 9. Effects of ManN on N-glycosylation enzymes, oligomannoses and monosaccharides in N-glycans. (a-c) Each enzyme was pre-incubated with various concentrations of ManN for 30 mins, before adding the corresponding substrate. (a) α-Mannosidase was inhibited by ManN at 400 μM, among the hexosamines tested. α-glucosidase

(b) and β-glucosidase (c) were not inhibited by ManN tested up to 2 mM. An average is shown. n=2 independent samples. (d) 40 μM ManN was used to treat BCECs grown in 10 cm dishes and cell pellets were collected at each time point for analysis. N-linked glycans were isolated by enzymatic cleavage followed by purification. N-glycans from 40 μg of total glycoprotein were tagged with 2-AB (2-Amino benzamide) and profiling was done using UPLC-FL and BEH-amide column. Areas of oligomannose N-glycans from various samples were quantified. Man-5 to Man-9 represents Man5GlcNAc2 to Man9GlcNAc2. (e) Analysis of monosaccharides in N-glycans. (f) Analysis of sialic acid in N-glycans. n=3 independent samples. A representative experiment is shown from 2 independent studies. Data are means +/- SD. Asterisks indicated a significant difference compared with the control. Statistical analysis was done by 2-tailed, two-sample unequal variance *t* test. * $p<0.05$, ** $p<0.01$. Data are provided as a Source Data file.

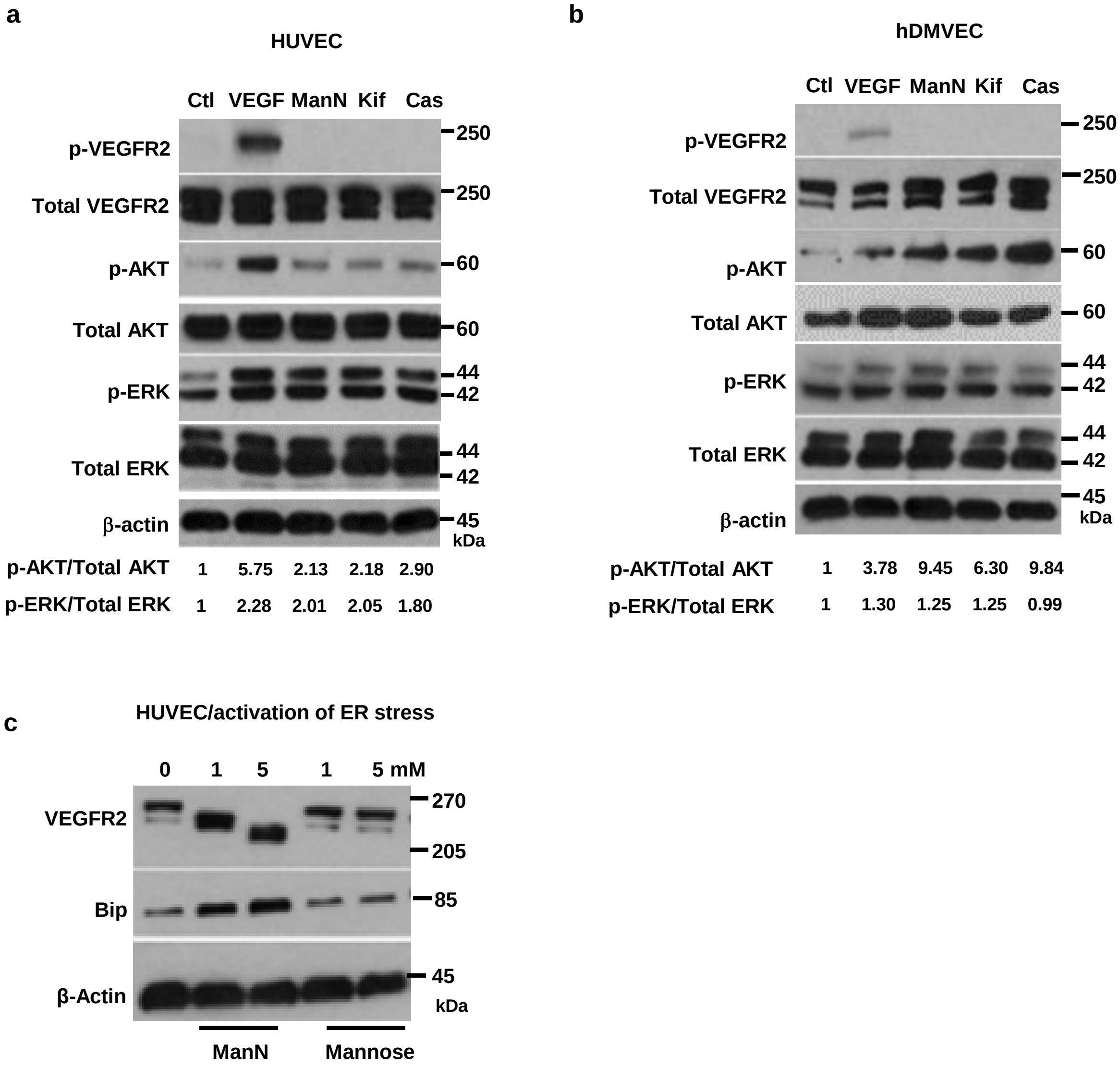

Supplementary Fig 10. ManN induces Bip expression and ERK and AKT phosphorylation in human endothelial cells. Serum-starved HUVEC (a) and hDMVECs (b) were stimulated with the glycosylation inhibitors Kif, Cas and ManN at 40 µM or with VEGF at 10 ng/ml for 30 mins (HUVEC) or 15 mins (hDMVECs). Cell lysates were collected and subjected to western blot analysis for phosphorylated VEGFR2 (Tyr1175), AKT (Ser473), ERK (Thr 202/Tyr 204), as well as total VEGFR2, AKT and ERK. Quantification of phosphorylated AKT and ERK was done by densitometry analysis relative to total protein. For each study, a representative experiment is shown.

(c) Induction of Bip expression in HUVEC by 2mM ManN, but not by mannose. β-actin served as the loading control. A representative experiment is shown from 2 independent studies. Data are provided as a Source Data file.

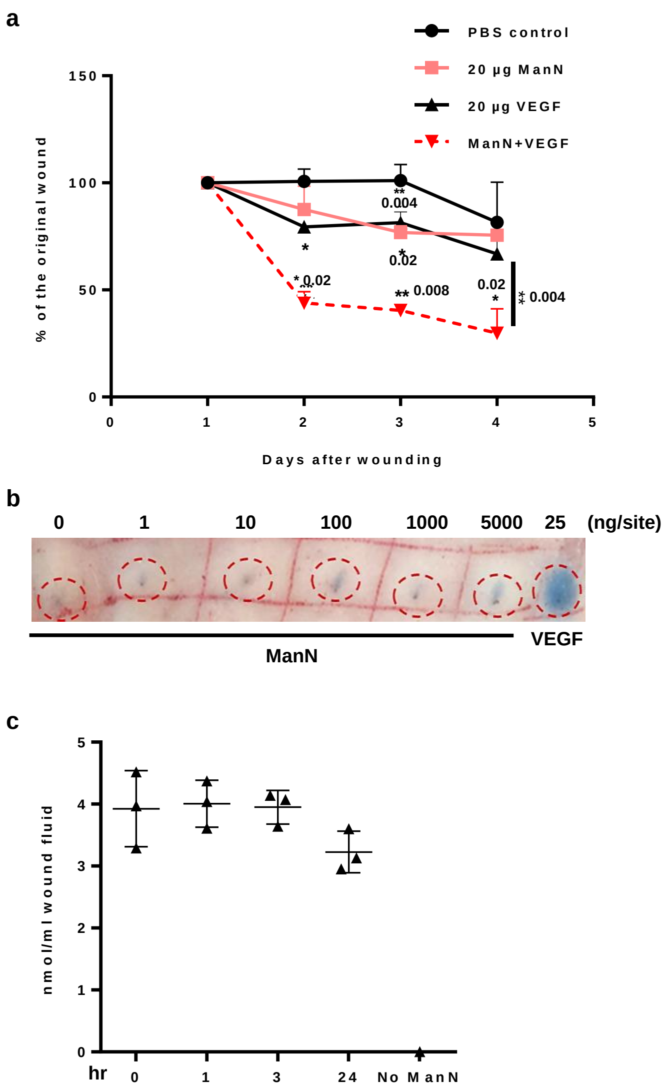

Supplementary Fig 11. Effects of ManN and VEGF in a mouse splinted wound healing model. (a) A significant stimulation of wound healing in ManN and VEGF combinatorial treatment group from a 4-day study. n=3 animals/treatment group. (b) ManN does not induce vascular permeability in the Miles assay. A representative image of guinea pig skin with the indicated treatments. VEGF, 25 ng/site, served as positive control. Red dotted circles indicate each injection site on the skin. n=2 independent samples. A representative experiment is shown

from 2 independent studies. (c) Stability of ManN in wound fluid from a skin infection model with *Staphylococcus aureus*. 1.5 µl of 5 % ManN solution was added to each 200 µl aliquot of wound fluid diluted 1:1 in PBS for various times. No ManN samples served as negative controls. A representative experiment is shown from 2 independent studies. Data are provided as a Source Data file.

a

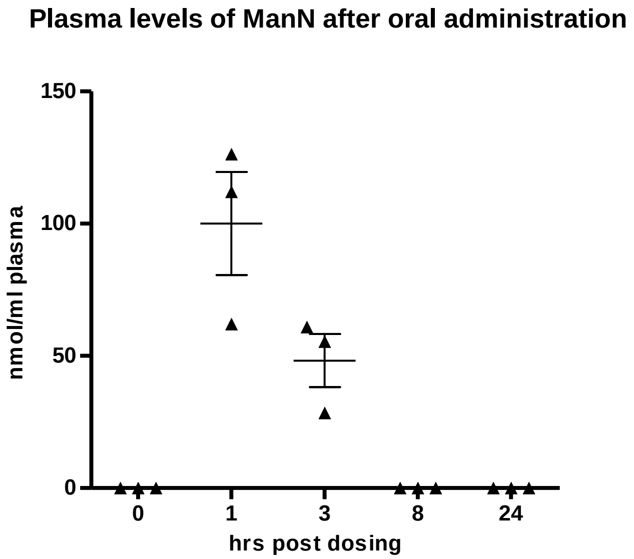

Supplementary Fig 12. Plasma levels of ManN following oral administration in C57BL/6 mice. 200 µl of 20% ManN was orally administrated. Plasma was collected at 1-24 hrs post dosing. Free ManN in wound fluid and plasma was measured using the Dionex ICS-3000 HPLC system with pulsed amperometric detector with standard Quad waveforms as described in Methods. ManN was identified and quantified by comparison with monosaccharide standards using ThermoFisher Scientific™ Chromeleon™ software. Samples in which ManN was not added (no ManN) were the negative controls. Asterisks denote significant difference compared with control. Data are means +/- SD from 3 independent samples. Statistical analysis was done by 2-tailed, two-sample unequal variance *t* test. * $p<0.05$, ** $p<0.01$. Data are provided as a Source Data file.

Supplementary Table 1 MALDI mass spectrum showing mono-isotopic ion intensities representing different classes of O-glycans (Mean or Mean +/- SD)

**Time course of 40 μM ManN treatment (hr)**	**Ion intensity (arbitrary units)**			
	**895** 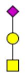	**983** 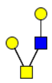	**1187** 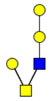	**1256** 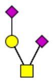
0 (n=2)	164	555	147	113
3 (n=2)	66	291	131	94
12 (n=3)	79+/-57	282+/-105	115+/-55	74+/-20

Supplementary Table 1. O-glycosylation analysis in BCECs treated with ManN by MALDI-TOF-MS. Cells were treated with 40 μM ManN for different time durations. Cell pellets were collected at 0, 3 and 12 hrs. Relative abundance of four O-glycans with ion intensity at *m/z* of 895, 983, 1187 and 1256 is shown. Data are means +/- SD from 2-3 independent samples or an average from 2 independent samples. Statistical analysis was done by 2-tailed, two-sample unequal variance *t* test. Data are provided as a Source Data file.

Supplementary Table 2 Cellular ManN uptake (Mean or Mean +/- SD)

**Cell Types**	**ManN**	**ManN-6p (nmol/mg protein)**	**Glucose**
A673 (n=2)	0.48	2.52	4.30
U87MG (n=4)	0.65+/-0.21	7.03+/-1.48	9.69+/-1.65
Calu6 (n=3)	1.01+/-0.39	1.72+/-1.24	3.67+/-4.16
4T1 (n=4)	0.74+/-0.09	3.91+/-1.66	8.03+/-1.8
hDMVEC (n=2)	0.99	2.72	30.9
HUVEC (n=2)	0.77	1.11	10.77
BCEC (n=2)	0.66	1.74	2.07
Bovine Pituitary (n=3)	0.58+/-0.05	2.02+/-0.15	2.37+/-0.98
AML12 (n=2)	1.20	1.47	21.84
Human dermal Fibroblast (n=3)	0.36+/-0.04	1.38+/-0.68	0.89+/-0.04
Human Keratinocyte (n=2)	1.58	0.69	2.30
Human RPE (n=2)	0.36	1.04	5.25

Supplementary Table 2. Cellular uptake of ManN by various cell types. Approximately 80% confluent cell cultures, grown on 60 mm dishes, were treated with 400 μM ManN for 2 hrs. Cell monolayers were extensively washed with PBS. Cell pellets were collected and subjected to HPAEC-PAD analysis. 1 nmol of ManN, Glucose and ManN-6p standards were used to quantify free ManN, ManN-6p and glucose present in the samples. The amount of monosaccharides present within different cells is presented as nmole/mg of total protein. All analyses were performed using Thermo-Dionex ICS system. Data are means +/- SD from 2-3 independent samples or an average from 2 independent biological samples. Data are provided as a Source Data file.

**Name**	**Padded amplicon**	**Catalog number**
From Thermo Fisher Scientific		
Bovine *VEGF-A*	GAGCCGCGCCGGCCCCGGCCAGGCCTCCGAAACCATGAACT<br>TTCTGCTCTCTTGGGTACATTGGAGCCTT<br>GCCTTGCTGCTCTACCTTCACCATGCCAAGTGGTCCCAGGCT<br>GCACCCATGGCAGAAGGAGGGCAGAAAC<br>CCCACGAAGTGGTGAAGTTCATGGATGTCTACCAGCGCAGC<br>TTCTGCCGTCCCATCGAGACCCTGGTGGA<br>CATCTTCCAGGAGTACCCAGATGAGATTGAGTTCATTTTCAA<br>GCCGTCCTGTGTGCCCCTGATGCGGTGC<br>GGGGGCTGCTGTAATGACGAA	Bt03213282
Bovine *VEGFR2*	TTTCTGGGCTTTGCGCCCGGCGCGAGGTGCGGGATGGAGA<br>GCAAGGCGCTGCTGGCCCTTGCTCTGTGGC<br>TCTGCGTGGAGACCCGGGCTGCCTCTGTGGGTTTTTCTAGT<br>GTTTCCCTTGATCCCCCCAGGCTCAGCAT<br>CCAAAAAGACATACTTAGAGTTATGGCTAACACAACGCTTC<br>AGATTACTTGCAGGGGTCAGAGGGACTTG<br>CAGTGGCTCTGGCCCAACAATCAGAGCAGCTCTGAGAAAAG<br>AGTGGAGGTCACA	Bt03258877
Bovine *GLUT1*	GAATTCGGGCATCCAGACAGTGGGCCGCCGGTCTCAGCAAC<br>TAGCTCGCTAGGCAGAGGGAGGCCAAGAG<br>AGTCGCAGACGGAGTCTCGAGACCGAAGCCGAAGCGGGAT<br>CCACAGAGCGCAGCCTGCCCGCGCACCCGG<br>TGCCGCGAAAGCCTCCAGCGCAGCGCGGCCATGGAGCCCA<br>CCAGCAAGAAGCTGACAGGCCGCCT	Bt03215313
Bovine *GLUT4*	GGGCCAGATTCCCGGGAGCTGCTGTCTGGCTCCTGGGCTTG<br>TGGCTGCGGATCTCGCCAGACTCGCACTC<br>CTCACGAAGCTCCGGGACCCCCATCACCCCCGCGTCCTCCGC<br>CCACCAGGCTGGGGTCGCAGACCCCCCA<br>GTCGCCTCCCCCTCGTCTACTGCAGCTCCTGCGTCTCCAGTTC<br>CTAAGACAAGATGCCGTCGGGCTTCCA ACAGATCGG	Bt03215316
Bovine *RPL19*	AAGCTCTTTCCTTTCGCCGCTGCGGCCGCGGCCATGAGTATG<br>CTCAGGCTTCAGAAGAGGCTTGCCTCCA<br>GTGTCCTTCGCTGTGGCAAGAAGAAAGTCTGGTTGGACCCC<br>AATGAGACCAATGAAATCGCCAATGCCAA<br>CTCCCGCCAGCAGATCCGGAAGCTGATCAAAGATGGGCTGA<br>TCATCCGGAAGCCTGTGACTGTCCATTCC<br>CGGGCTCGATGCCGGAAAAACA	Bt03229687

Supplementary Table 3. Primers
