# Supplementary material for: Inhibition of protein glycosylation is a novel pro-angiogenic strategy that acts via activation of stress pathways
Source: Nat Commun. 2020 Dec 10;11:6330. doi: 10.1038/s41467-020-20108-0 (PMC7730427; doi:10.1038/s41467-020-20108-0)
Supplement: Supplementary file 2 — Reporting Summary [file 41467_2020_20108_MOESM2_ESM.pdf]

nature research

Corresponding author(s): Napoleone Ferrara NCOMMS-19-26641A

Last updated by author(s): 10/02/2020

Reporting Summary

Nature Research wishes to improve the reproducibility of the work that we publish. This form provides structure for consistency and transparency in reporting. For further information on Nature Research policies, see Authors & Referees and the Editorial Policy Checklist .

Statistics

For all statistical analyses, confirm that the following items are present in the figure legend, table legend, main text, or Methods section.

n/a	Confirmed	
☐	☒	The exact sample size (*n*) for each experimental group/condition, given as a discrete number and unit of measurement
☒	☐	A statement on whether measurements were taken from distinct samples or whether the same sample was measured repeatedly
☐	☒	The statistical test(s) used AND whether they are one- or two-sided<br>*Only common tests should be described solely by name; describe more complex techniques in the Methods section.*
☐	☒	A description of all covariates tested
☐	☒	A description of any assumptions or corrections, such as tests of normality and adjustment for multiple comparisons
☐	☒	A full description of the statistical parameters including central tendency (e.g. means) or other basic estimates (e.g. regression coefficient) AND variation (e.g. standard deviation) or associated estimates of uncertainty (e.g. confidence intervals)
☐	☒	For null hypothesis testing, the test statistic (e.g. *F*, *t*, *r*) with confidence intervals, effect sizes, degrees of freedom and *P* value noted<br>*Give P values as exact values whenever suitable.*
☒	☐	For Bayesian analysis, information on the choice of priors and Markov chain Monte Carlo settings
☒	☐	For hierarchical and complex designs, identification of the appropriate level for tests and full reporting of outcomes
☒	☐	Estimates of effect sizes (e.g. Cohen's *d*, Pearson's *r*), indicating how they were calculated

Our web collection on statistics for biologists contains articles on many of the points above.

Software and code

Policy information about availability of computer code

Data collection	N/A
Data analysis	N/A

For manuscripts utilizing custom algorithms or software that are central to the research but not yet described in published literature, software must be made available to editors/reviewers. We strongly encourage code deposition in a community repository (e.g. GitHub). See the Nature Research guidelines for submitting code & software for further information.

Data

Policy information about availability of data

All manuscripts must include a data availability statement. This statement should provide the following information, where applicable:

- Accession codes, unique identifiers, or web links for publicly available datasets
- A list of figures that have associated raw data
- A description of any restrictions on data availability

All data supporting the findings are included in this published article (and its Supplementary files), and all relevant materials are available from the authors. No genomic or microarray sets were generated in this study.

Field-specific reporting

Please select the one below that is the best fit for your research. If you are not sure, read the appropriate sections before making your selection.

☒ Life sciences ☐ Behavioural & social sciences ☐ Ecological, evolutionary & environmental sciences

Life sciences study design

All studies must disclose on these points even when the disclosure is negative.

Sample size	For cell-based assays, three wells or three independently-treated samples were used.<br>Wound healing model was conducted in 5 animals/treatment group.<br>Permeability model was conducted on two animals, as published in Cell. 2016, 167(1):275-284
Data exclusions	N/A
Replication	2-3
Randomization	Mice were allocated randomly to different groups.
Blinding	Some key observations were conducted by more than one person in the lab.

Reporting for specific materials, systems and methods

We require information from authors about some types of materials, experimental systems and methods used in many studies. Here, indicate whether each material, system or method listed is relevant to your study. If you are not sure if a list item applies to your research, read the appropriate section before selecting a response.

Materials & experimental systems

n/a	Involved in the study	
☐	☒	Antibodies
☐	☒	Eukaryotic cell lines
☒	☐	Palaeontology
☐	☒	Animals and other organisms
☒	☐	Human research participants
☒	☐	Clinical data

Methods

n/a	Involved in the study	
☒	☐	ChIP-seq
☒	☐	Flow cytometry
☒	☐	MRI-based neuroimaging

Antibodies

Antibodies used	Antibodies were from Cell Signaling and Sigma.
Validation	All antibodies were from reputable sources and have been extensively validated in the literatures. In our hands, they all interacted with proteins of the expected molecular mass and functional activation.

Eukaryotic cell lines

Policy information about cell lines

Cell line source(s)	Bovine retinal microvascular endothelial cells (BRECs) and bovine choroidal microvascular endothelial cells (BCECs) were from VEC technologies; Human retinal microvascular endothelial cells (passage <15) was from Cell Systems Corporation; Normal human retinal pigment epithelial cells were obtained from Lonza; alll other cell lines were obtained from ATCC.
Authentication	All cell lines are from Lonza, ATCC or VEC technologies
Mycoplasma contamination	No contamination of mycoplasma in any of the cell line we used.
Commonly misidentified lines<br>(See ICLAC register)	N/A

Animals and other organisms

Policy information about studies involving animals; ARRIVE guidelines recommended for reporting animal research

Laboratory animals	C57BL/6 male or female mice 6–10 week of age and HA-Hrhr/IAF male guinea pigs 450-500g
Wild animals	N/A
Field-collected samples	All mouse procedures were approved by the University of California, San Diego Institutional Animal Care and Use Committee.

Ethics oversight

All mouse procedures were approved by the University of California, San Diego Institutional Animal Care and Use Committee.

Note that full information on the approval of the study protocol must also be provided in the manuscript.
