# Supplementary material for: Inhibition of protein glycosylation is a novel pro-angiogenic strategy that acts via activation of stress pathways
Source: Nat Commun. 2020 Dec 10;11:6330. doi: 10.1038/s41467-020-20108-0 (PMC7730427; doi:10.1038/s41467-020-20108-0)

Fig.2a

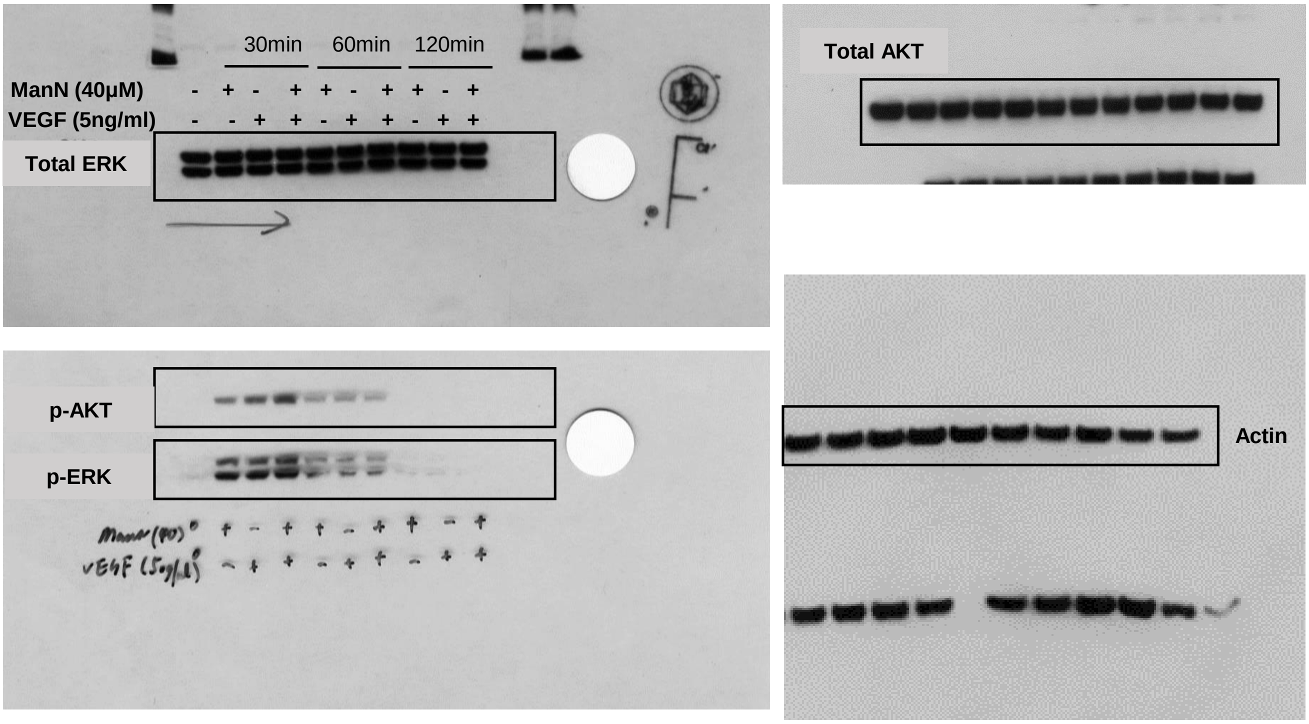
30min
60min
120min
ManN (40μM) - + - + + - + + - +
VEGF (5ng/ml) - - + + - + + - + +
Total ERK
p-AKT
p-ERK
Total AKT
Actin

Fig.2b

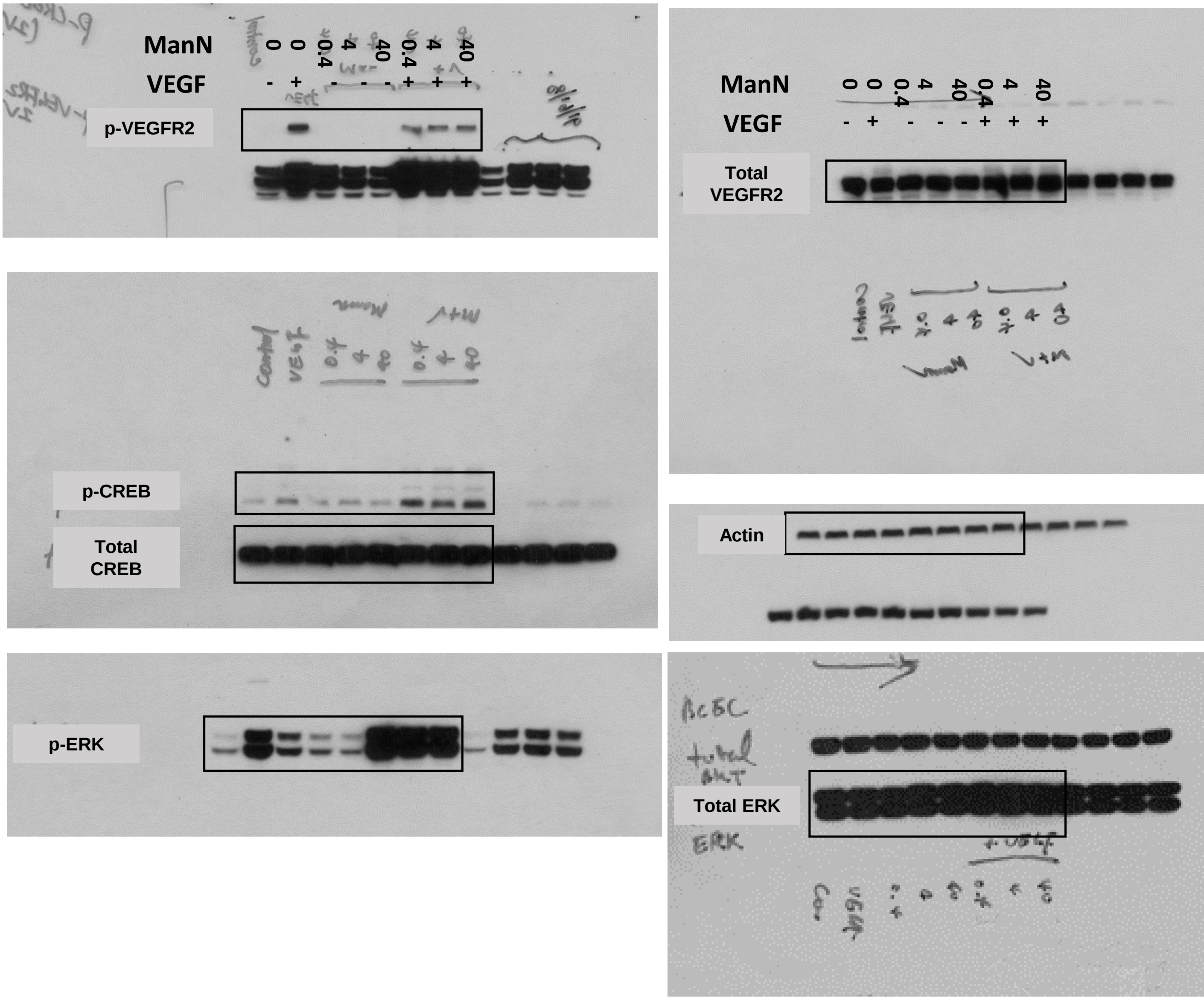
ManN
0 0 0.4 4 40 0.4 4 40
VEGF
- + - - - + + +
p-VEGFR2
Total VEGFR2
p-CREB
Total CREB
Actin
p-ERK
Total ERK

Fig.2c

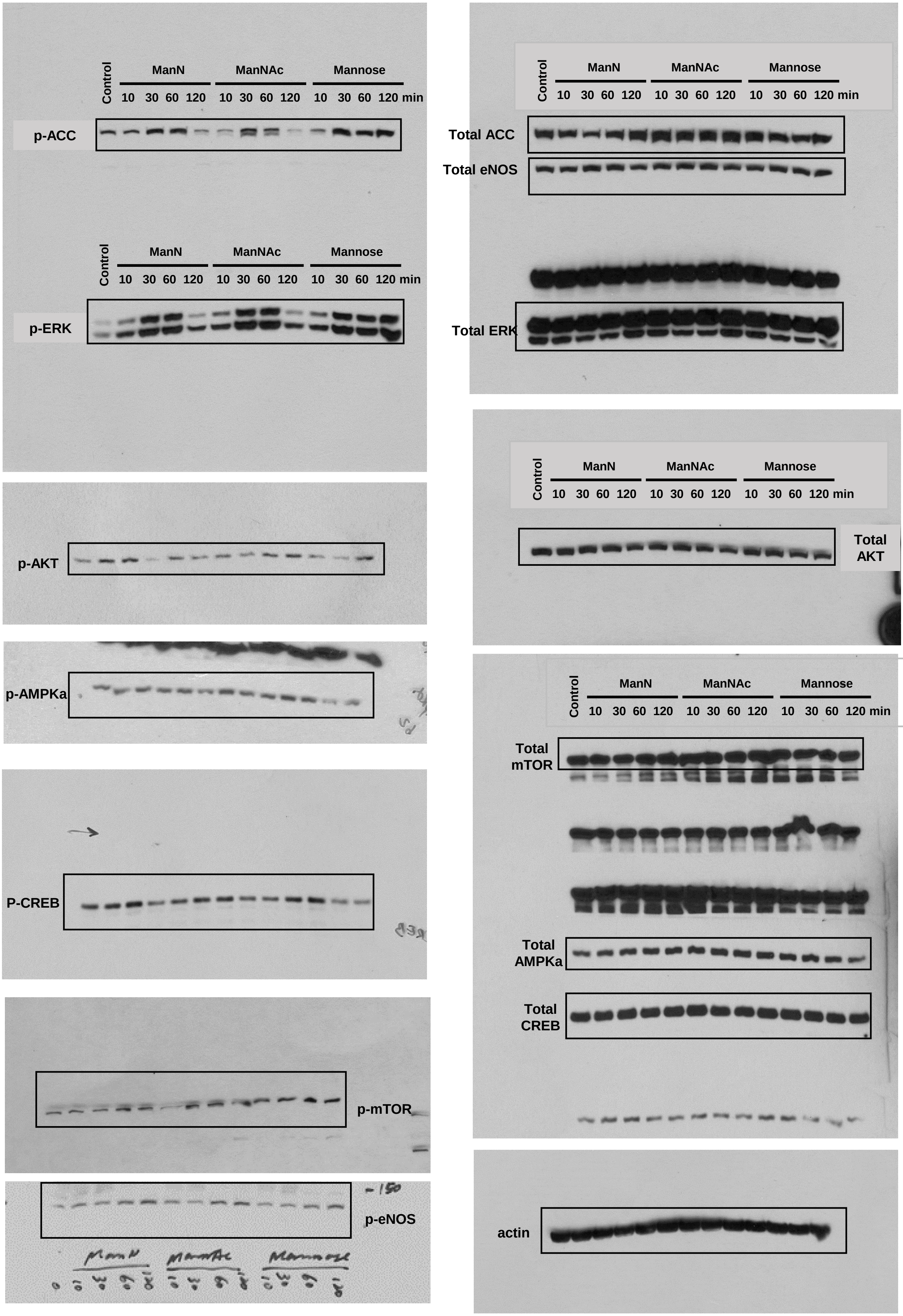

Control
ManN
ManNAc
Mannose
10 30 60 120 10 30 60 120 10 30 60 120 min
p-ACC
p-ERK
p-AKT
p-AMPKa
P-CREB
p-mTOR
p-eNOS
Total ACC
Total eNOS
Total ERK
Total AKT
Total mTOR
Total AMPKa
Total CREB
actin

Fig.3a

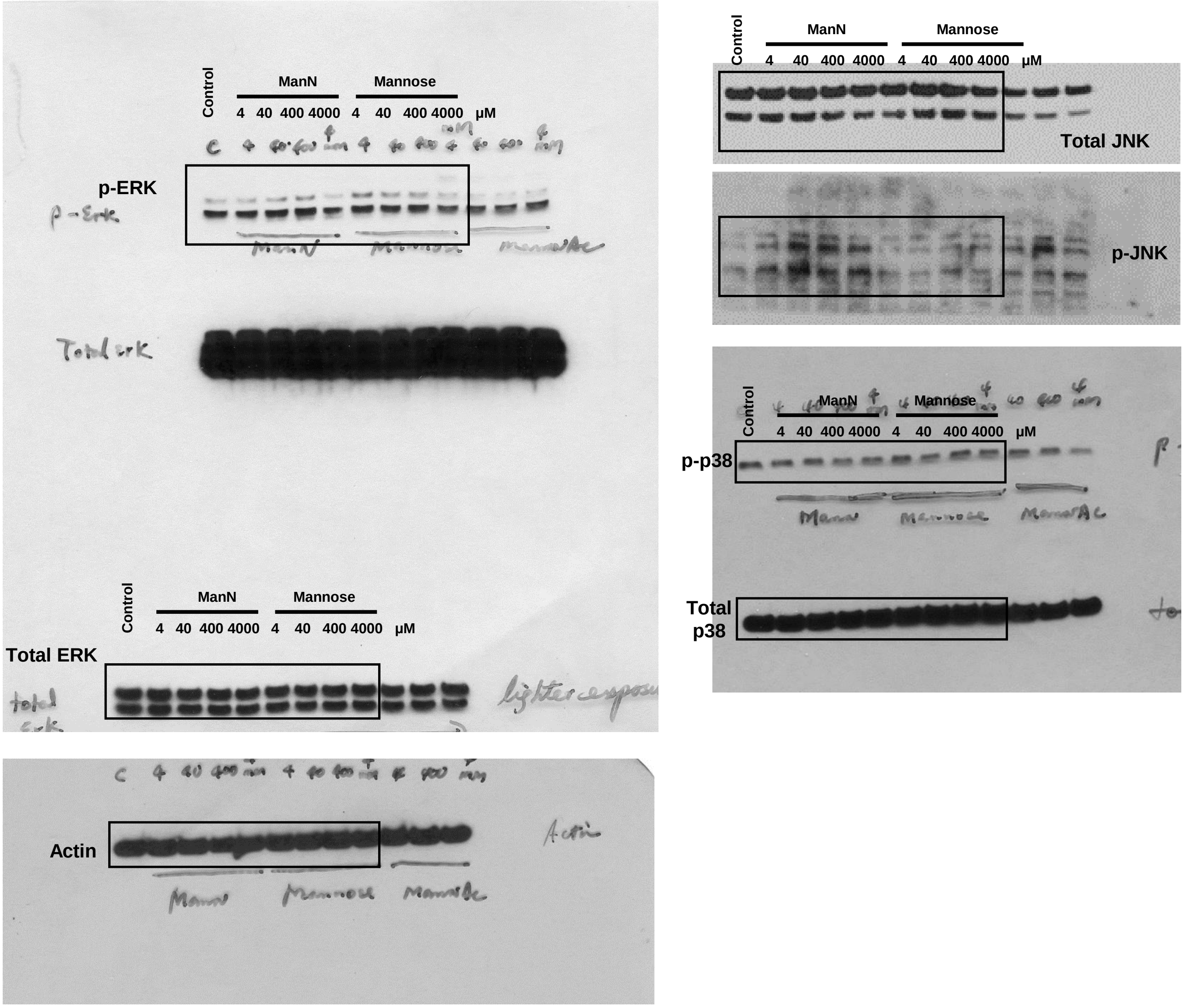
Control
ManN
Mannose
4 40 400 4000 4 40 400 4000 µM
p-ERK
Total ERK
Total JNK
p-JNK
p-p38
Total p38
Actin

Fig.3b

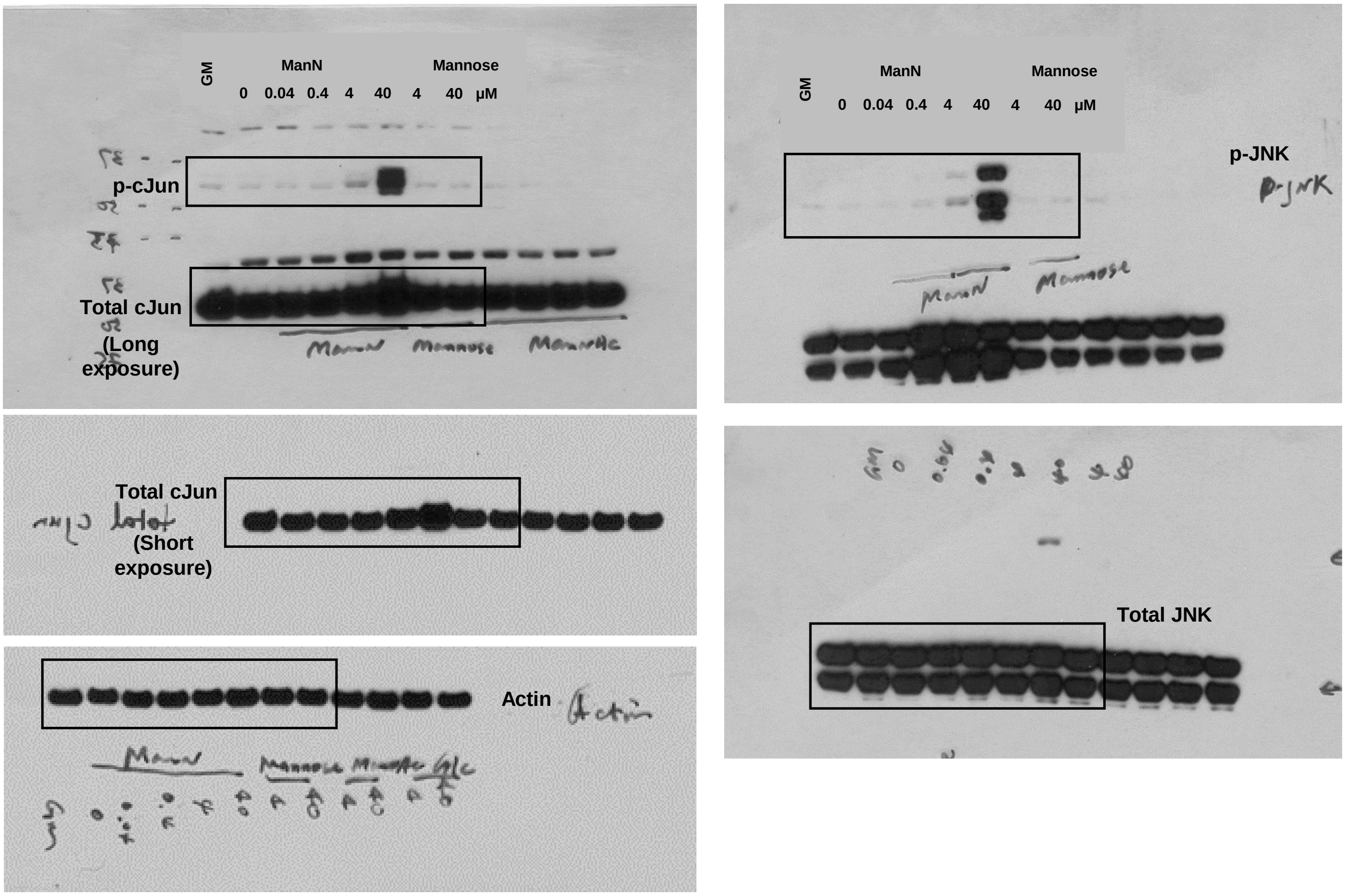
GM
ManN
Mannose
0 0.04 0.4 4 40 4 40 μM
p-cJun
Total cJun
(Long exposure)
Total cJun
(Short exposure)
Actin
p-JNK
Total JNK

Fig.3d

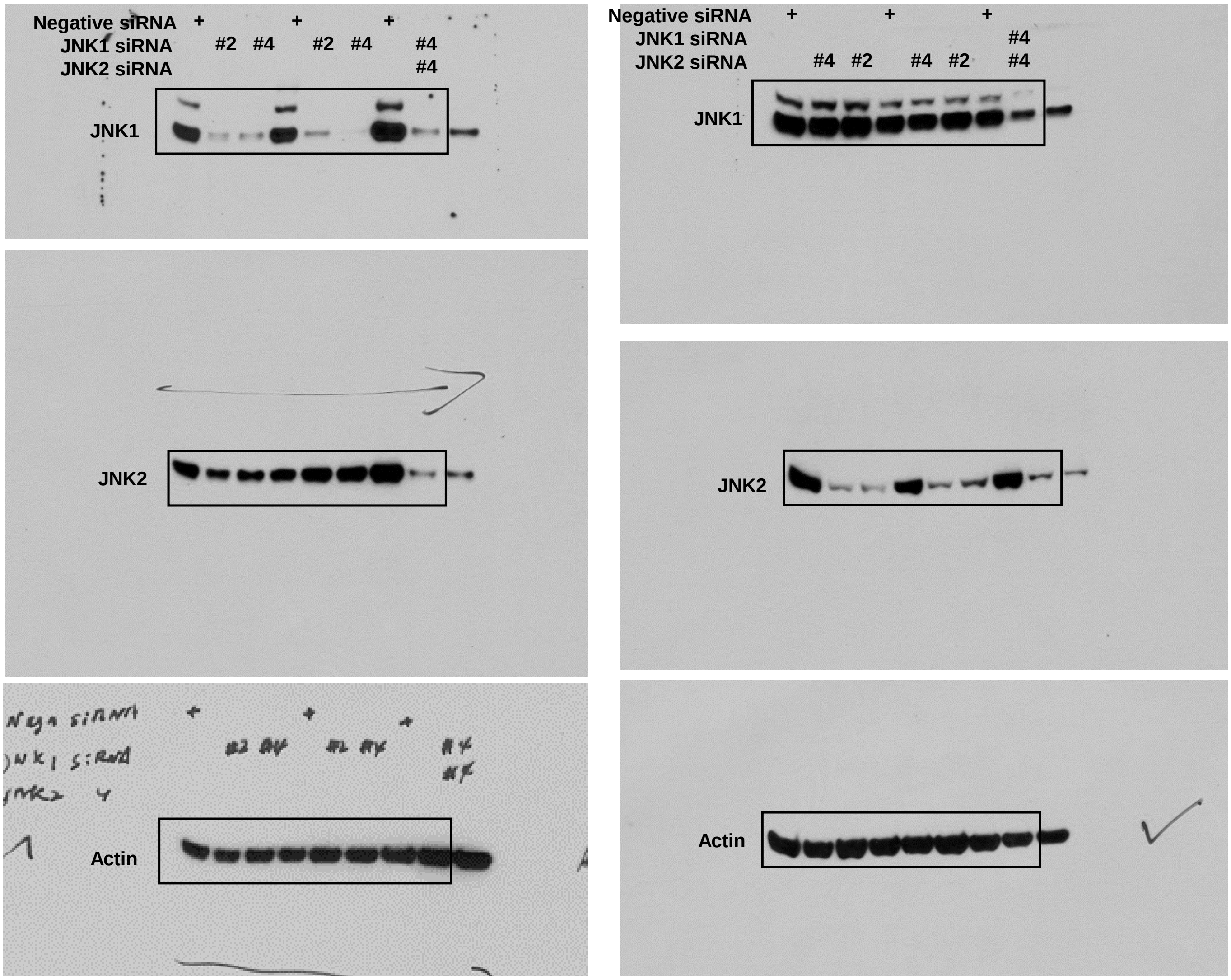
Negative siRNA + + +
JNK1 siRNA #2 #4 #2 #4 #4
JNK2 siRNA #4
JNK1
JNK2
Actin
Negative siRNA + + +
JNK1 siRNA #4
JNK2 siRNA #4 #2 #4 #2 #4
JNK1
JNK2
Actin

Fig.4a

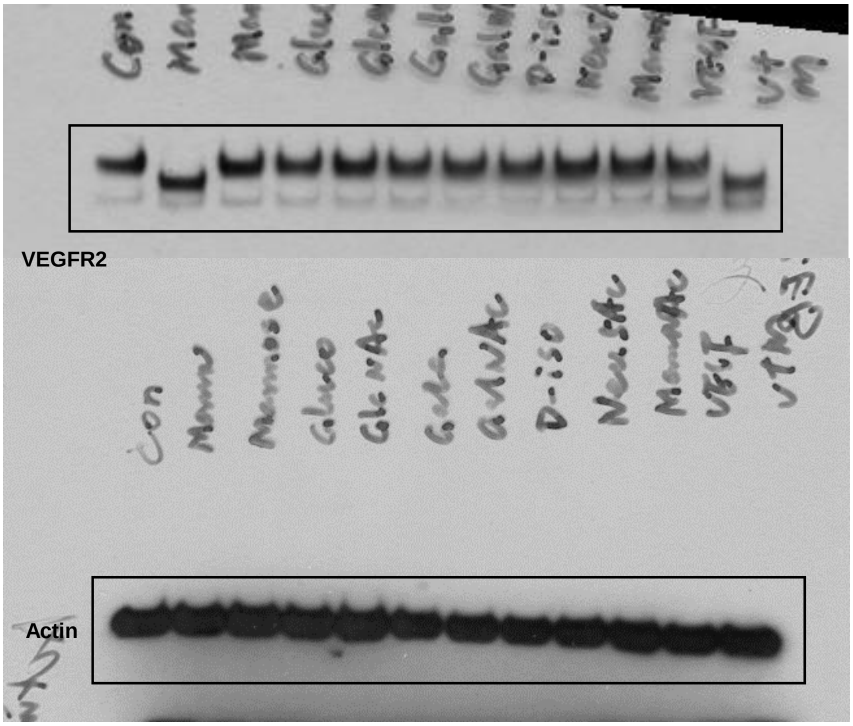

Fig.4b

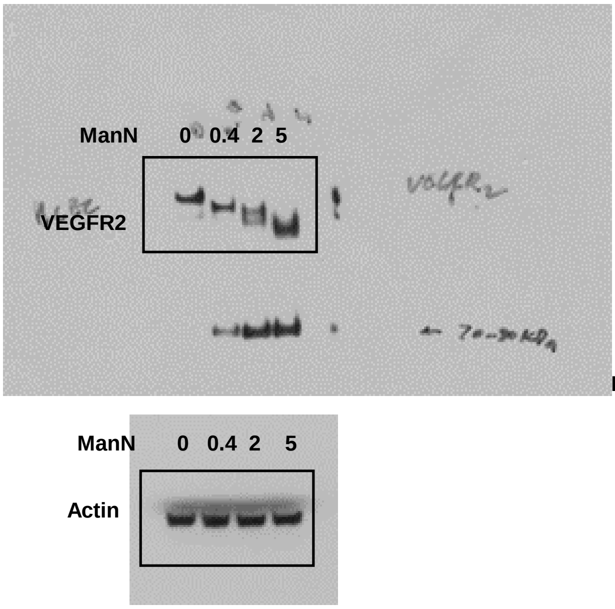

Fig.4c

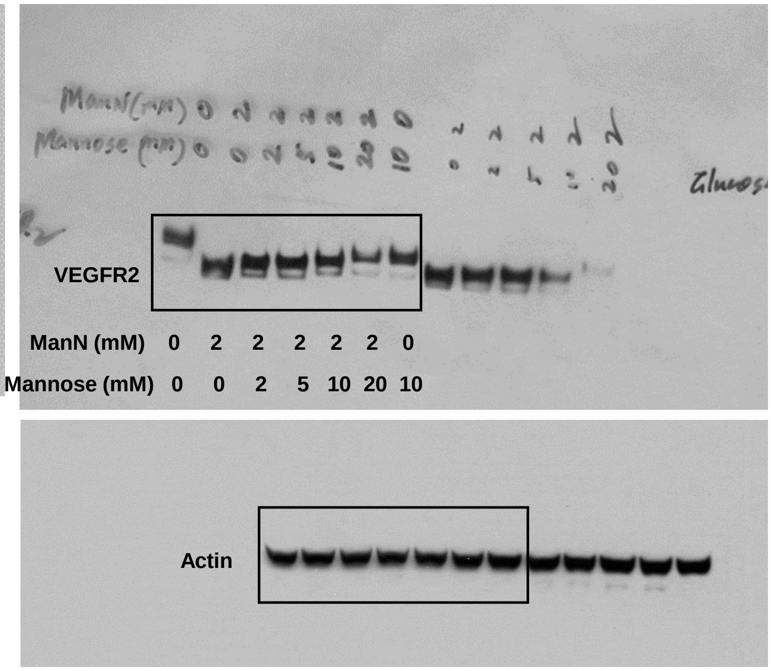

Fig.4e

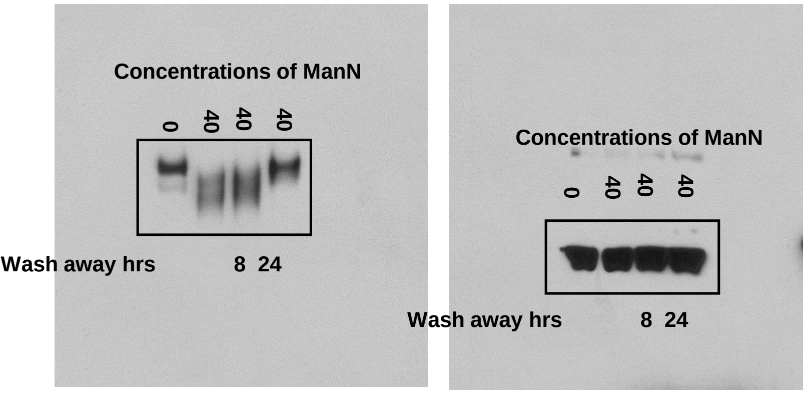

Fig.4f

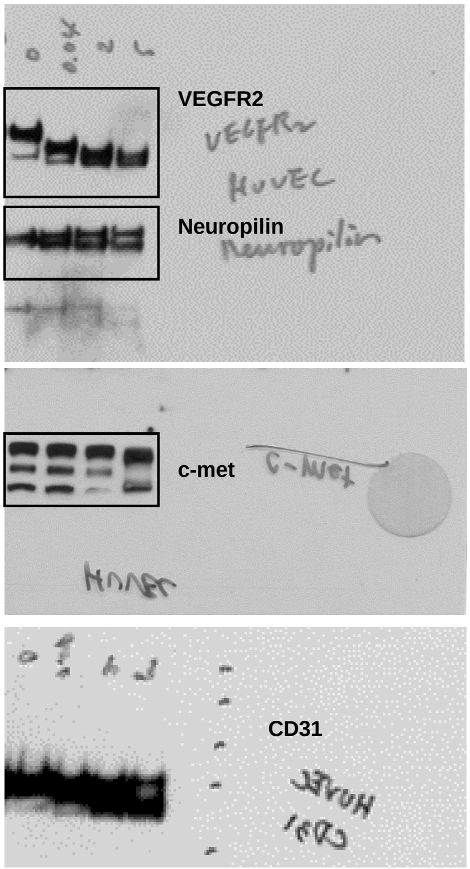
VEGFR2
Neuropilin
c-met
CD31

Fig.4g

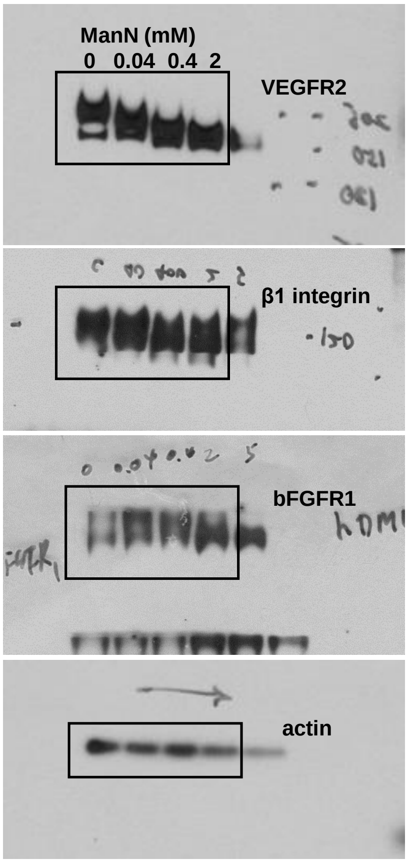
ManN (mM)
0 0.04 0.4 2
VEGFR2
β1 integrin
bFGFR1
actin

Fig.5a

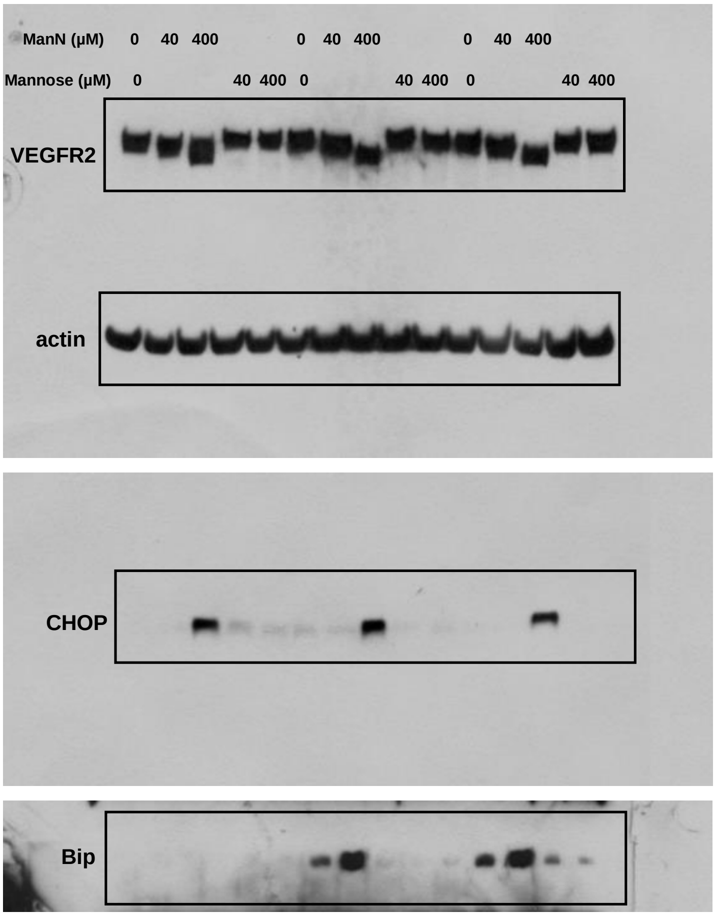

Fig.5b

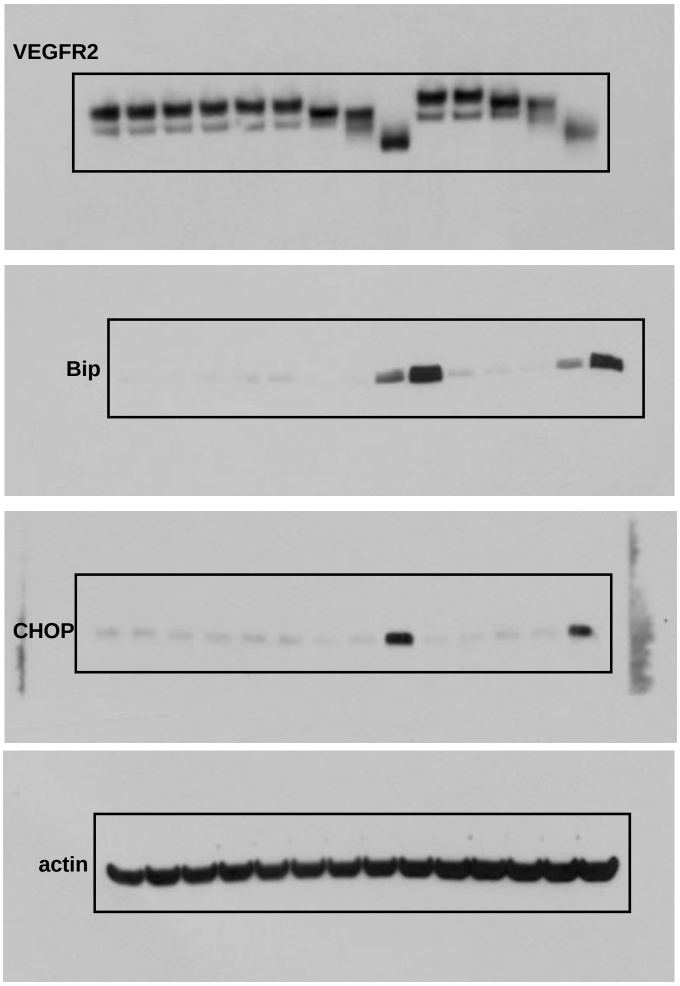

Fig.5c

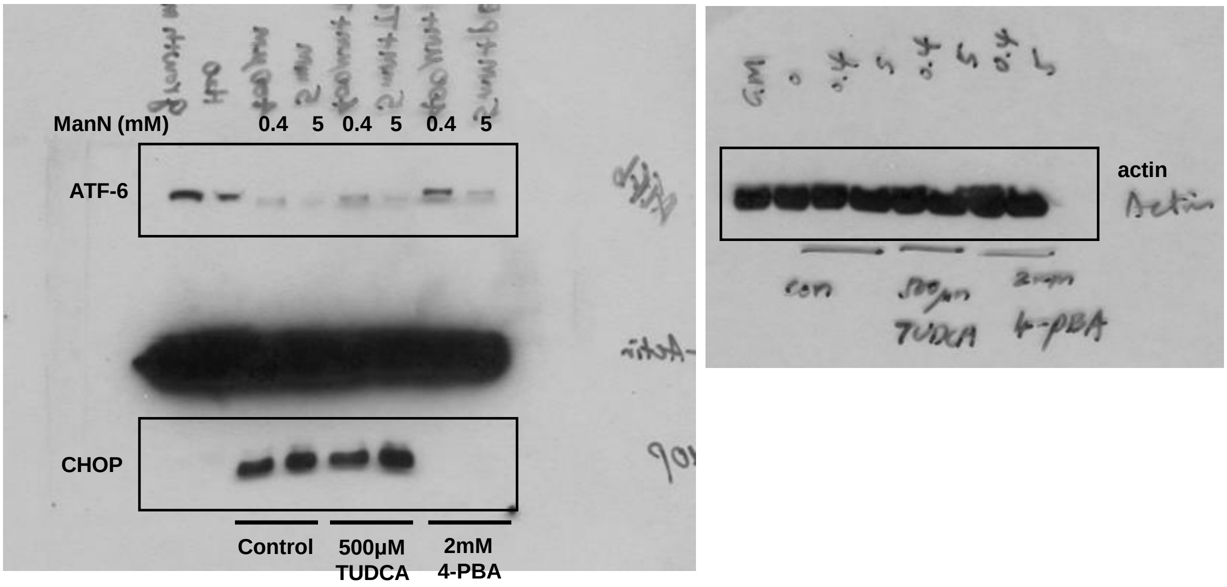

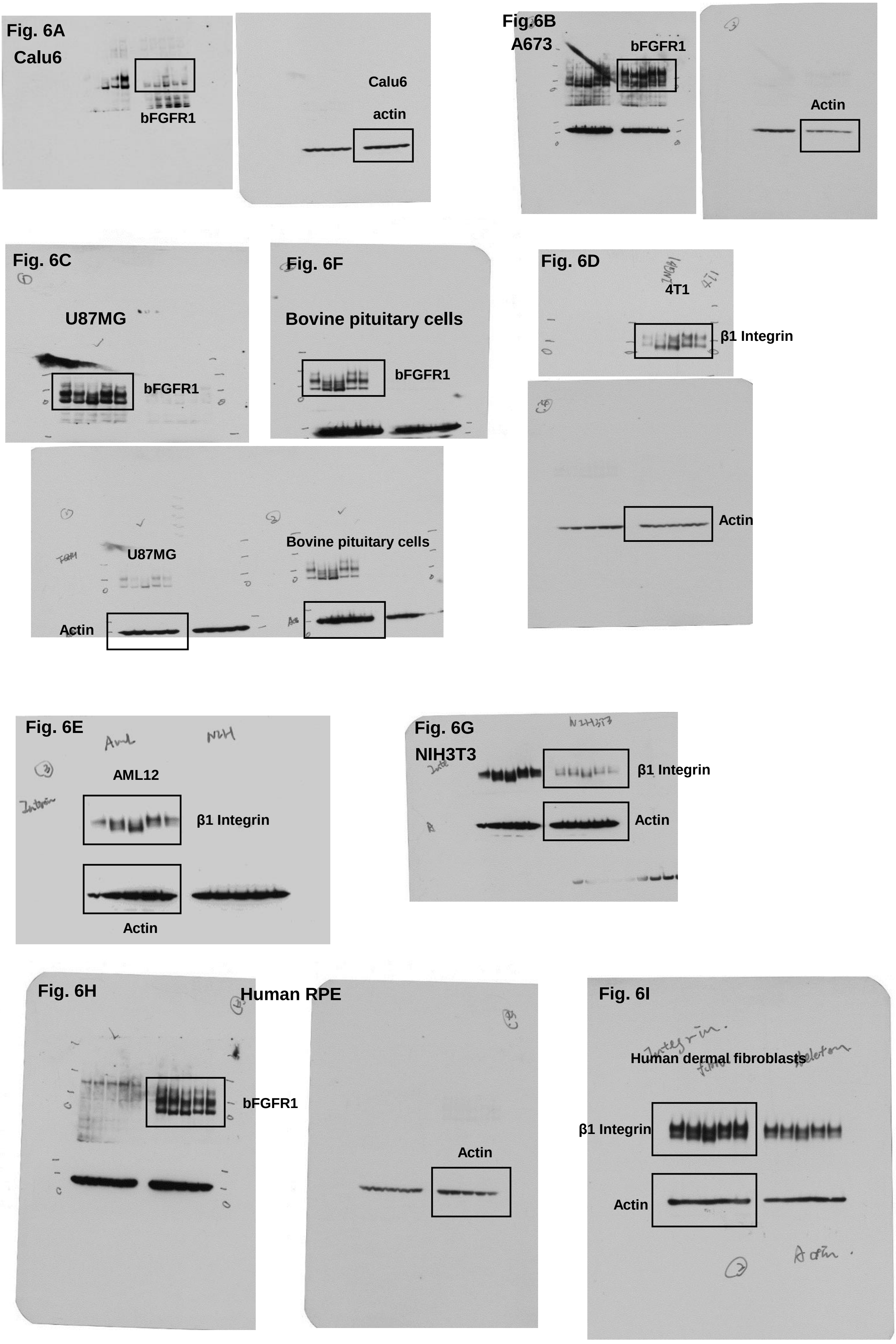
Fig. 6A
Calu6
bFGFR1
Calu6
actin
Fig.6B
A673
bFGFR1
Actin
Fig. 6C
U87MG
bFGFR1
Fig. 6F
Bovine pituitary cells
bFGFR1
Fig. 6D
4T1
β1 Integrin
U87MG
Bovine pituitary cells
Actin
Actin
Fig. 6E
AML12
β1 Integrin
Actin
Fig. 6G
NIH3T3
β1 Integrin
Actin
Fig. 6H
Human RPE
bFGFR1
Actin
Fig. 6I
Human dermal fibroblasts
β1 Integrin
Actin

Fig. 7c

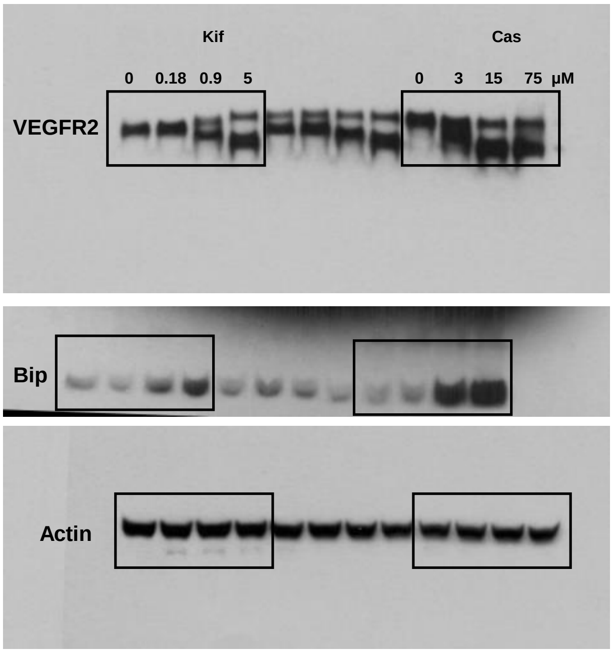

Fig. 7e

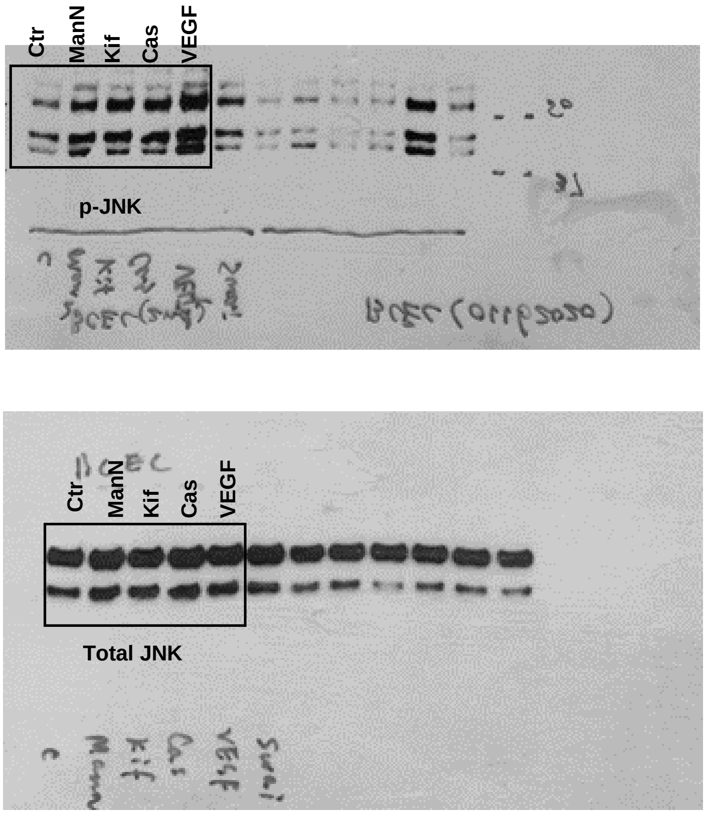

Fig. 7e

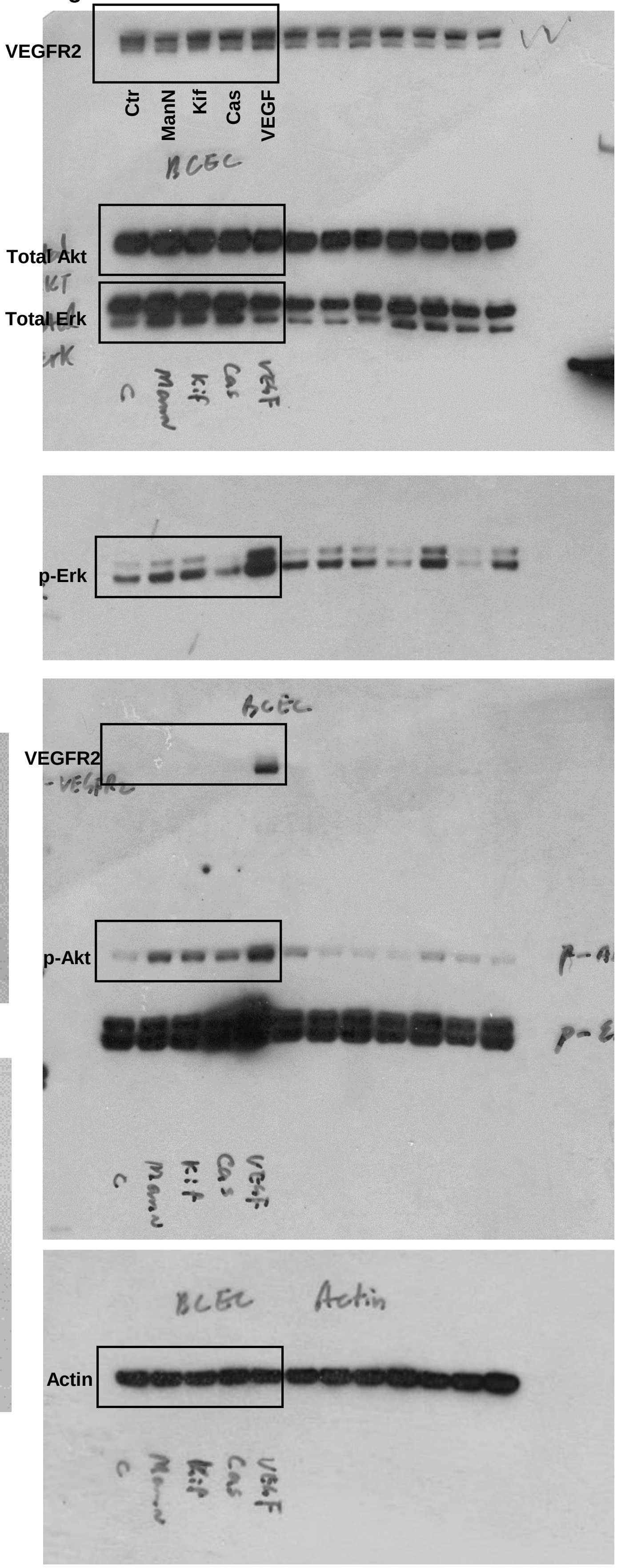

SFig. 4b

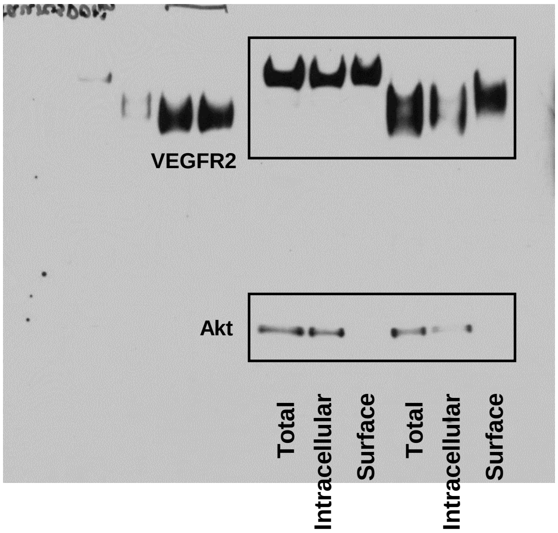

SFig. 4c

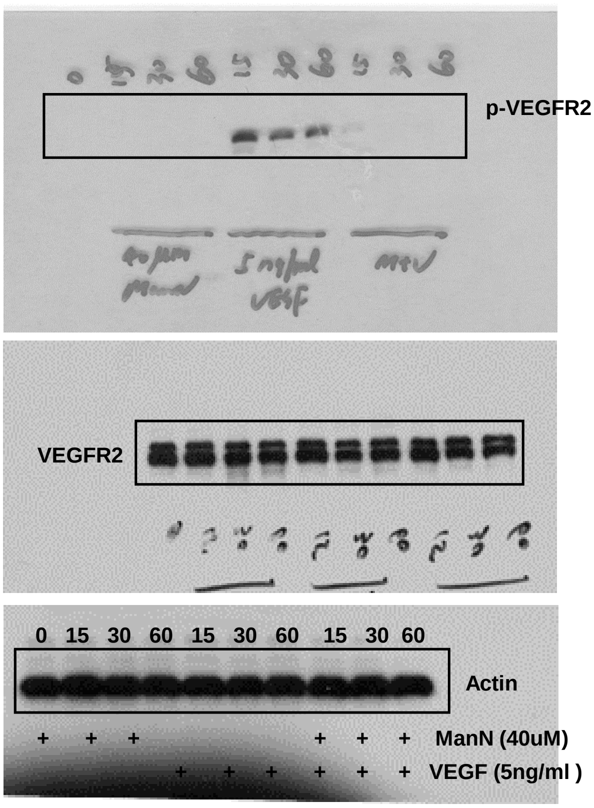

SFig. 5a

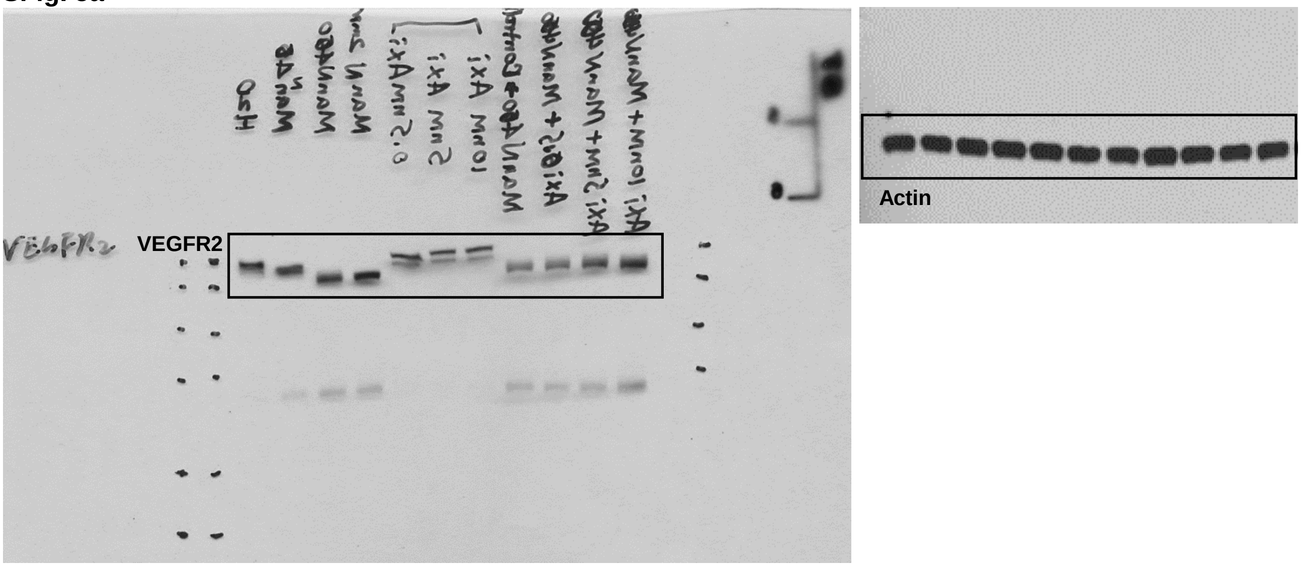

SFig. 6a

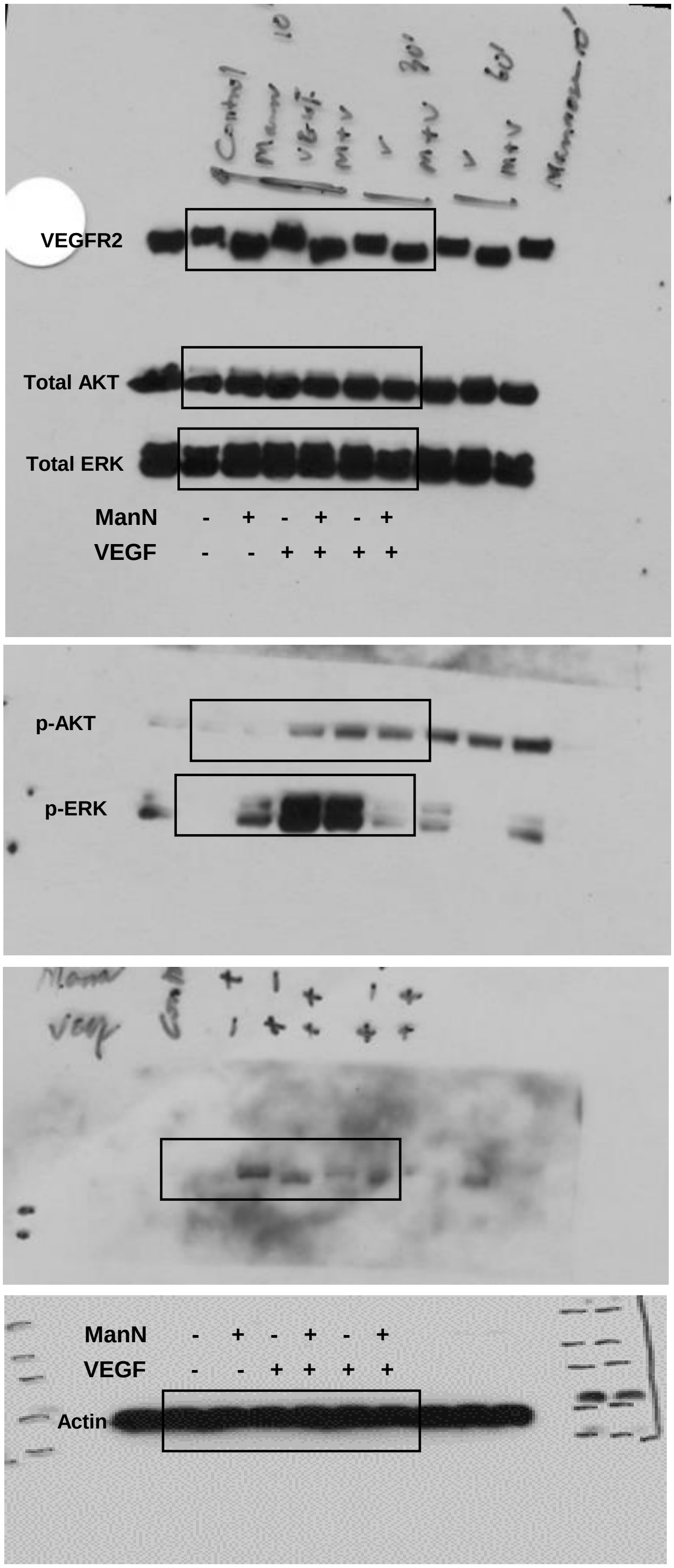

SFig. 6b

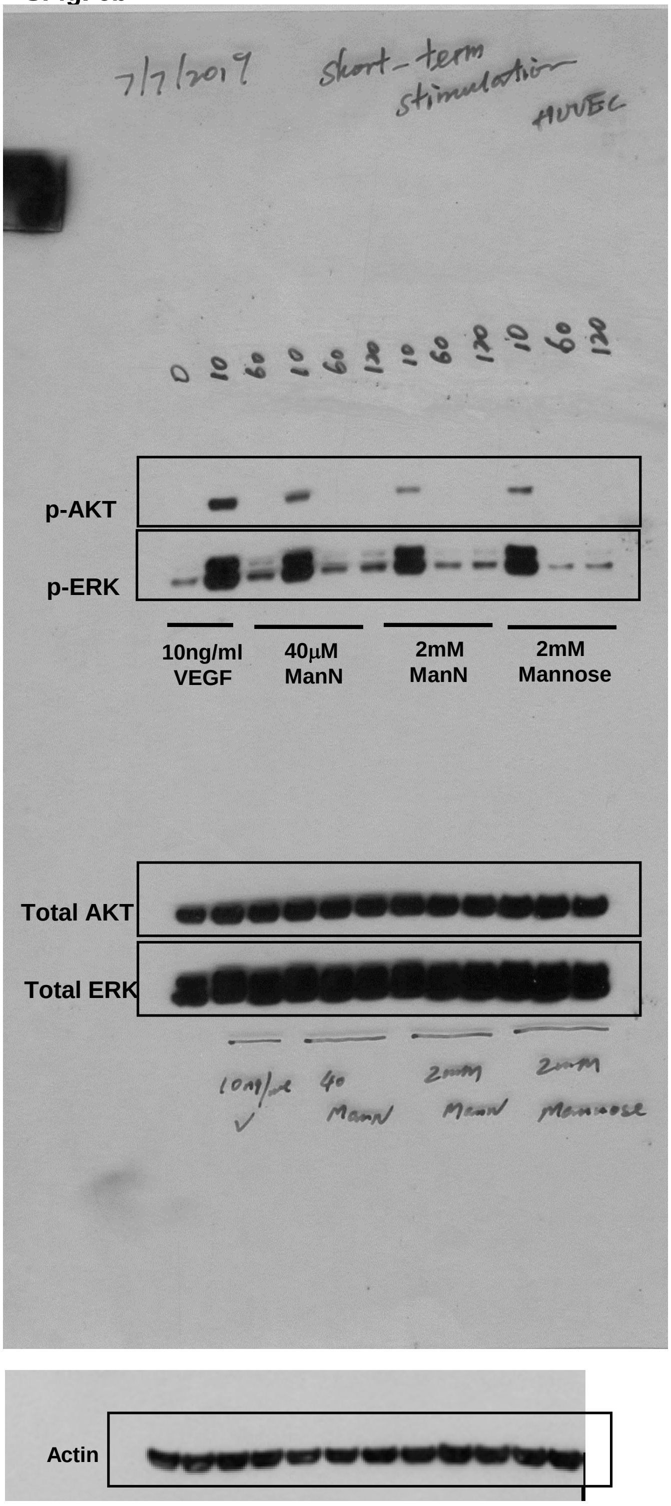

SFig. 7a

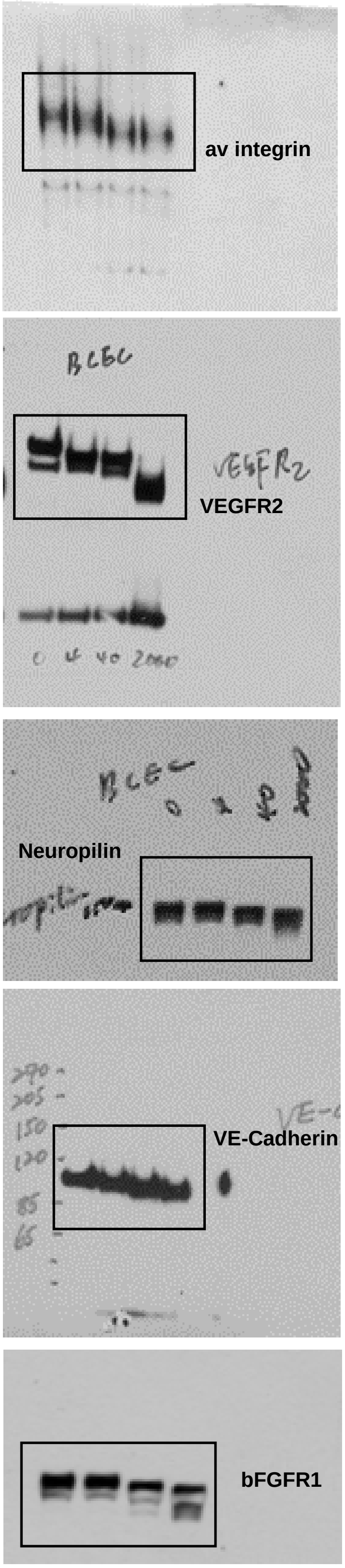

SFig. 7b & c

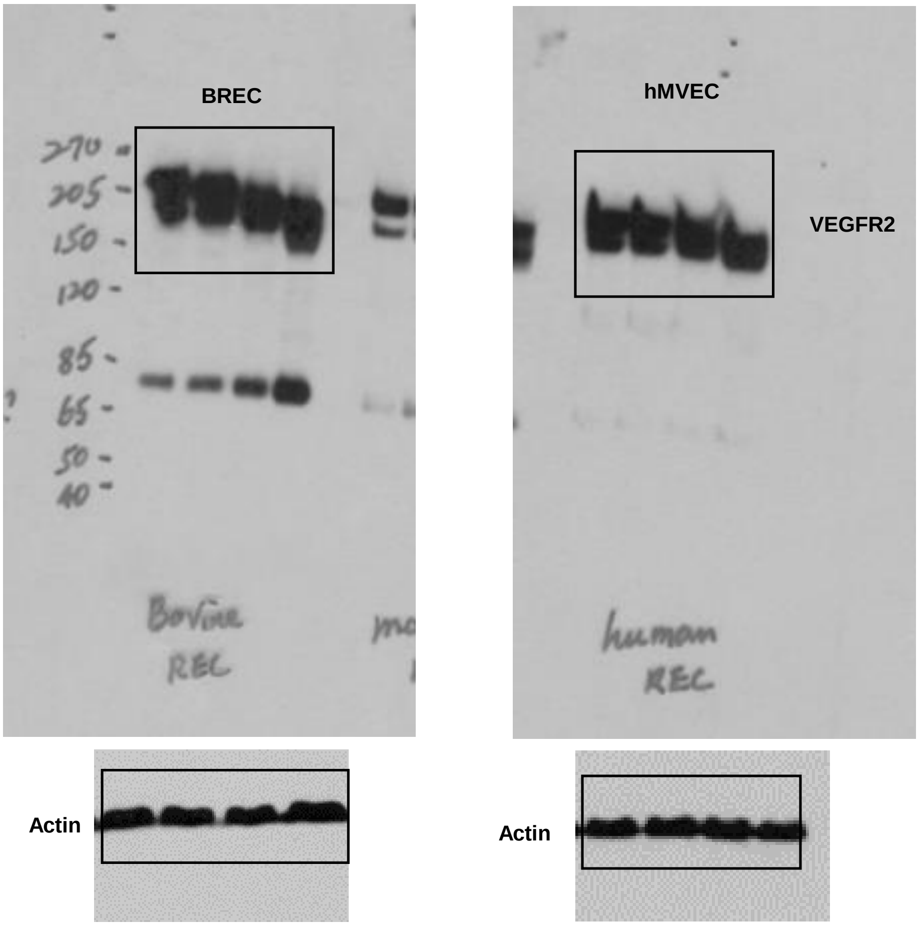

SFig. 7d

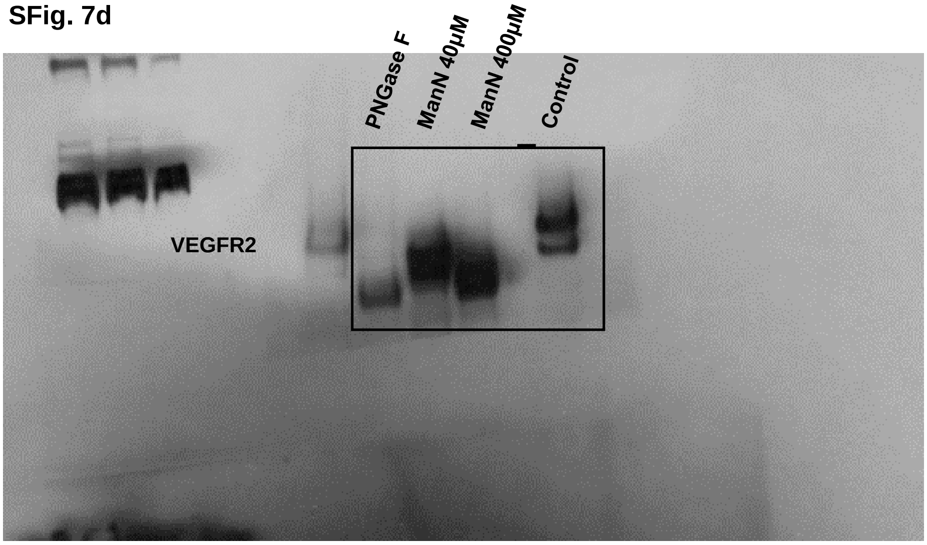

Sfig.8a

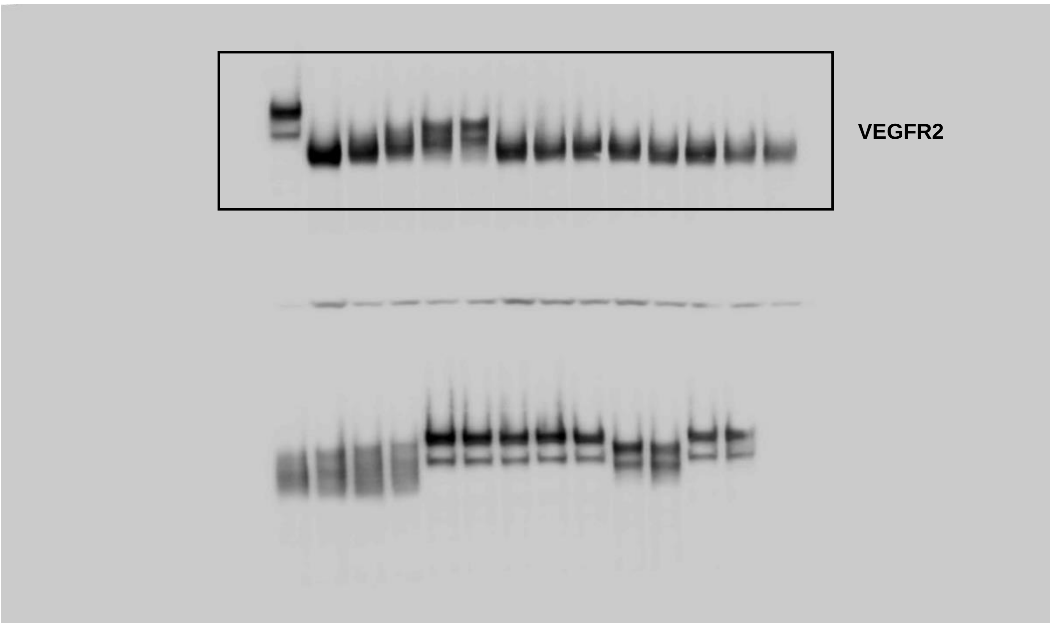

Sfig.10a&b

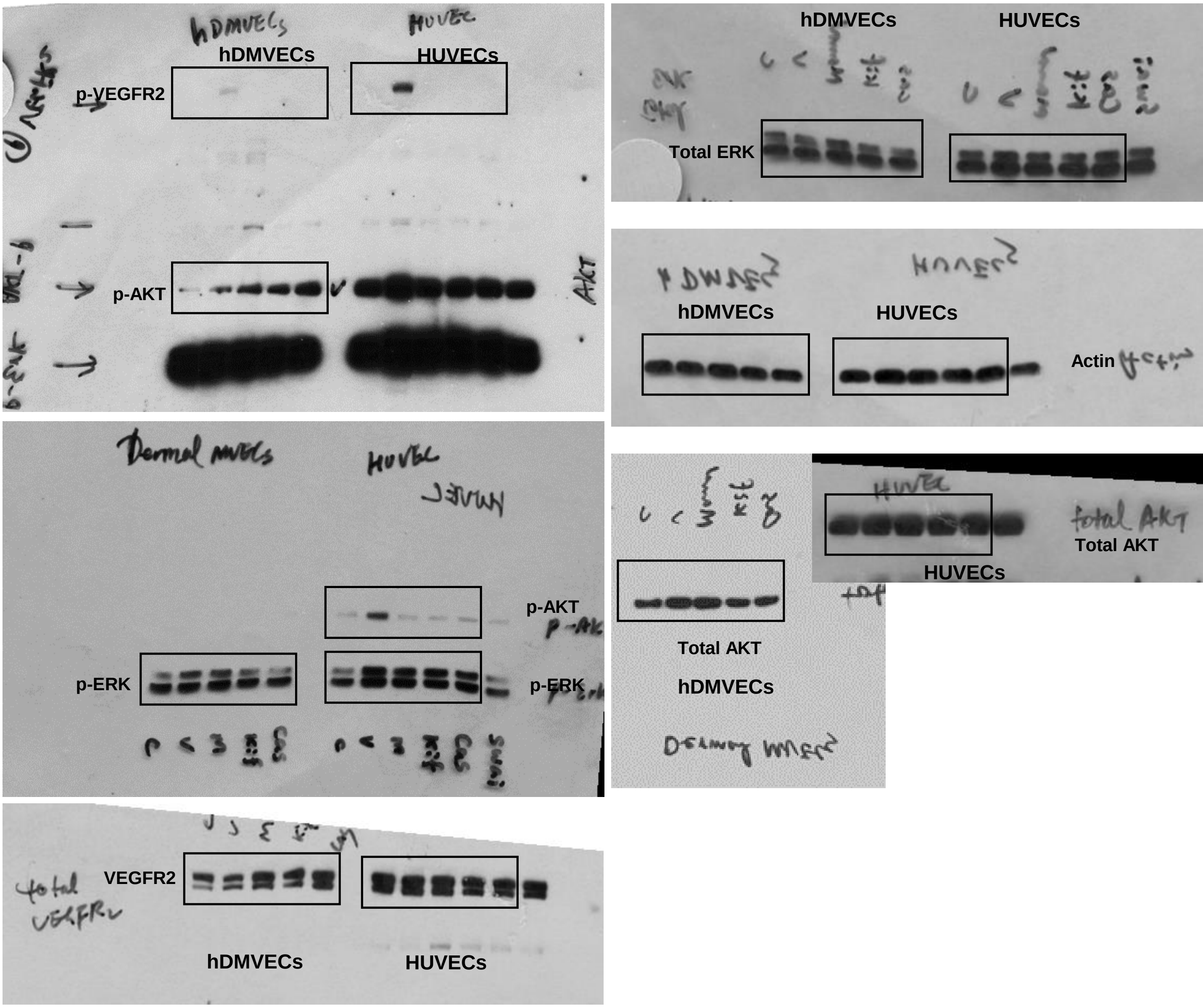
hDMVECs
HUVECs
p-VEGFR2
p-AKT
hDMVECs
HUVECs
Total ERK
hDMVECs
HUVECs
Actin
p-AKT
p-ERK
p-ERK
Total AKT
HUVECs
Total AKT
hDMVECs
VEGFR2
hDMVECs
HUVECs

Sfig.10c

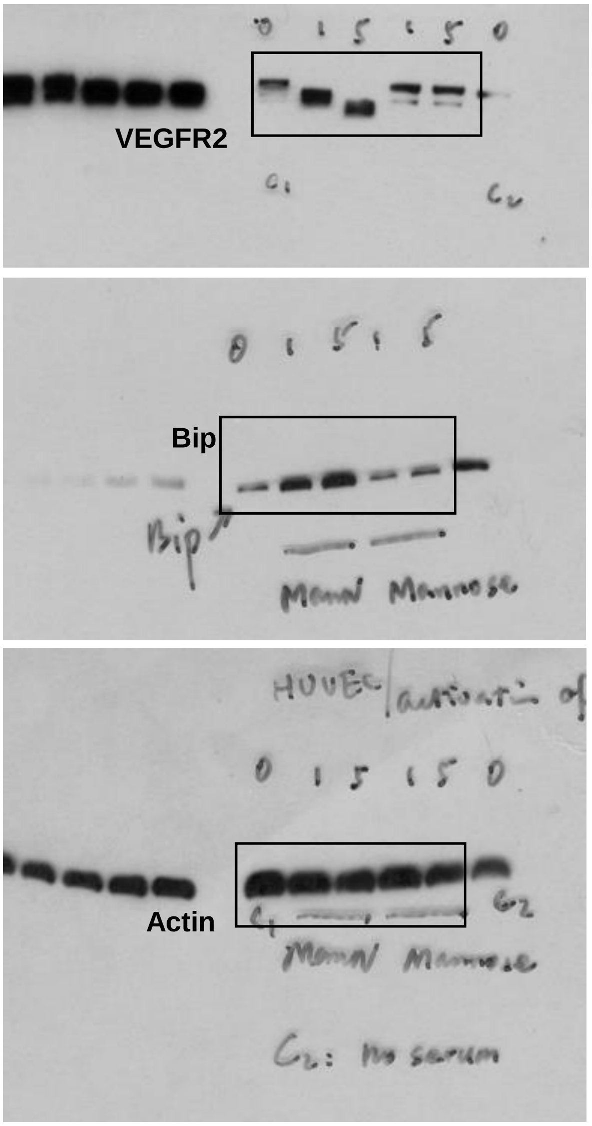

Supplement: Supplementary file 3 — Source Data [file 41467_2020_20108_MOESM3_ESM.zip › Zhong et al source data file-western.pdf]
